# CHICKN: extraction of peptide chromatographic elution profiles from large scale mass spectrometry data by means of Wasserstein compressive hierarchical cluster analysis

**DOI:** 10.1186/s12859-021-03969-0

**Published:** 2021-02-12

**Authors:** Olga Permiakova, Romain Guibert, Alexandra Kraut, Thomas Fortin, Anne-Marie Hesse, Thomas Burger

**Affiliations:** 1 Univ. Grenoble Alpes, CEA, Inserm, BGE U1038, 38000 Grenoble, France; 2 Univ. Grenoble Alpes, CNRS, CEA, Inserm, BGE U1038, 38000 Grenoble, France

**Keywords:** Large-scale cluster analysis, Liquid chromatography, Mass spectrometry, Proteomics, Wasserstein kernel, Optimal transport

## Abstract

**Background:**

The clustering of data produced by liquid chromatography coupled to mass spectrometry analyses (LC-MS data) has recently gained interest to extract meaningful chemical or biological patterns. However, recent instrumental pipelines deliver data which size, dimensionality and expected number of clusters are too large to be processed by classical machine learning algorithms, so that most of the state-of-the-art relies on single pass linkage-based algorithms.

**Results:**

We propose a clustering algorithm that solves the powerful but computationally demanding kernel *k*-means objective function in a scalable way. As a result, it can process LC-MS data in an acceptable time on a multicore machine. To do so, we combine three essential features: a compressive data representation, Nyström approximation and a hierarchical strategy. In addition, we propose new kernels based on optimal transport, which interprets as intuitive similarity measures between chromatographic elution profiles.

**Conclusions:**

Our method, referred to as CHICKN, is evaluated on proteomics data produced in our lab, as well as on benchmark data coming from the literature. From a computational viewpoint, it is particularly efficient on raw LC-MS data. From a data analysis viewpoint, it provides clusters which differ from those resulting from state-of-the-art methods, while achieving similar performances. This highlights the complementarity of differently principle algorithms to extract the best from complex LC-MS data.

## Background

Liquid chromatography coupled to mass spectrometry (LC-MS) constitute a technological pipeline that has become ubiquitous in various omics investigations, such as proteomics, lipidomics and metabolomics. Over the past decade, the MS throughput has continuously improved, leading to unprecedented data volume production. To date, processing these gigabytes of low level MS signals has become a challenge on its own, for a trade-off between contradictory objectives is sought: On the one hand, one needs to save memory and computational time with efficient encoding, compression and signal cleaning methods [1]. On the other hand, one needs to avoid too important preprocessing that systematically smoothes signals of lower magnitudes, as it is now well-established that interesting biological patterns can be found near the noise level [2]. To face this challenge, a recent and efficient investigation path has been to apply cluster analysis to LC-MS data. Cluster analysis refers to a large family of unsupervised statistical learning and multivariate analysis techniques which share a common goal: Aggregating similar data items into clusters, so that within-cluster similarities are larger than between cluster ones. By doing so, it becomes possible to consider the various clusters independently, and thus to reduce the computational footprint without any quality loss. Moreover, as each cluster contains similar data elements, it facilitates the extraction of repetitive but small biological patterns.

### State of the art

To date and contrarily to the presented work, investigations have mainly focused on clustering LC-MS data across the chromatographic (or elution time) dimension, *i.e.* when the data elements are MS spectra: MS2grouper [3, 4], Pep-Miner [5], PepMerger [6], the MS-Clustering/ MS-Cluster/Pride-Cluster/spectra-cluster series [7–10], Bonanza [11], CAMS-RS [12], MaRaCluster [13], N-cluster [14], and msCRUSH [15]. All these approaches propose to improve peptide identification by benefiting from the aforementioned trade-off: By grouping similar fragmentation spectra into a consensus representation, one clearly reduces the data volume. Moreover, peaks corresponding to random noise should not reinforce between spectra, while on the contrary, small but chemically consistent peaks should [16].

Clustering across the mass-to-charge ratio (*m*/*z*) dimension, *i.e.* when the data elements are chromatographic profiles (depicting the signal changes along the elution time at a given *m*/*z* value), is also insightful for many reasons: First, it proposes an original framework to construct and extract precursor ion chromatograms, which integration is essential for quantitative analysis [17]. Second, cluster centroids naturally provide consensus elution profiles which are of interest for retention time alignment [18]. Finally, elution profiles are also essential to disentangle chimeric spectra [19]. Notably if the clustering is sufficiently accurate, it can be insightful to disentangle multiplexed acquisitions (*e.g.* Data Independent Acquisition [20], or DIA), without relying on spectral libraries [21, 22]. To date, these practical problems have been tackled in the proteomics literature by applying various heuristics which differ to some extend from the cluster analysis framework. For instance, in DIA-Umpire [23], peptide fragments’ elution profiles are clustered according to their correlations with precursor profiles, so that formally, the approach is more that of classification (*i.e.* supervised) than of clustering (*i.e.* unsupervised). Similarly, in many quantification algorithms (Maxquant [24], OpenMS [25], MsInspect [26], Xnet [17]) cluster analysis aims to extract isotopic envelopes, *i.e.* to group the elution profiles of several isotopes of a given molecule, within a closed neighborhood of *m/z* values. As a consequence, two identical profiles in different *m/z* regions are not grouped together. Although this behavior (that will be referred to as the *envelope assumption simplification* in the rest of the article) concurs with the objective of isotopic envelope reconstruction, it makes the heuristic strongly attached to one objective; and non applicable to other cluster analysis problems. In contrast, we believe generic clustering algorithms would also be of interest, as different tuning would make them appropriate to deal with different objectives: *e.g.* by adding must-link/must-not-link constraints [27] so as to guide the demultiplexing task as in the DIA-Umpire case; or by incorporating an *m/z* difference in the similarity definition, in the case of isotopic envelope extraction; and so on.

Moreover, a refine analysis of the algorithms underlying all these (either spectrum or chromatogram) clustering techniques let appear a strong filiation between them: All rely on agglomerative and linkage-based methods, be it previously published algorithms (HAC [28, 29], DBSCAN [30] or UPGMA [31]) or *ad-hoc* procedures developed in the specific context of LC-MS data clustering (proposed in MS2grouper, Pep-Miner, PepMerger, the MS-Cluster series, Bonanza, CAMS-RS, N-cluster and XNet). Despite their unquestionable efficiency, some diversity would help. Cluster analysis is as much an art as a science [32] and there does not exist such thing as the perfect clustering – at least, on real data. Most of the time, data analysts need to rely on a toolbox of various algorithms to extract the best of their data [33]. With this respect, MS-based omics would benefit from differently principled and complementary algorithms which have demonstrated their efficiency in data science [34]. For instance, spectral clustering [35–37] (which should not be confused with the cluster analysis of mass spectra [38]), mean shift algorithm [39, 40], and variants of the *k*-medoids [41] and *k*-means [42, 43] are of prime interest.

Finally, one observes a difference between algorithms dedicated to spectrum clustering and those dedicated to chromatogram clustering: While the former ones are mainly implemented in an independent manner, the latter ones are all embedded in computational pipelines (DIA-Umpire [23], Maxquant [24], OpenMS [25], MsInspect [26]). The only exception is Xnet [17], which makes it a unique literature reference for algorithmic and low-level comparisons. In addition, Xnet is the most recently published algorithm, and it displays interesting performances on a benchmark dataset.

In a nutshell, Xnet is a Bayesian algorithm which aims to cluster elution profiles into isotopic envelopes. More precisely, it starts from the construction of a network with chromatograms as nodes. Then, the network is decomposed into preliminary clusters. The edges within each cluster are scored by estimating the likelihood of two parameters: the correlation between chromatograms and their *m*/*z* separation. Finally, the edge validation is carried out using the scores and a chromatogram apex match verification. This leads to the final isotopic envelope construction.

Xnet has many strengths: First, it is a parameter free clustering method – the number of clusters can be inferred during the learning process. Second, the time complexity of the algorithm is linear with respect to the number of chromatograms in the data. However, it also has weaknesses: First, it cannot work on raw data and requires an important preprocessing step, referred to as *ion chromatogram extraction*, which denoizes the LC-MS map and aggregates independent measurements into well-formed *traces* (*i.e.* lists of peak intensities corresponding to a same ion, identified in consecutive mass spectra). Concretely, starting from a raw file, it is first necessary to extract non trivial information and to store them into an input CSV file with the following columns: *m*/*z* ratios, retention times, intensities and trace labels. In addition to be time consuming, it can arguably be considered that excluding the trace construction from the algorithm amounts to transferring a bottleneck question to another preliminary processing, or to a human annotator. Second, it strongly relies on the envelope assumption simplification, making it impossible to group elution profiles which *m*/*z* difference exceeds a predefined threshold. The third weakness is related to the generalization capabilities: As acknowledged in [17], there is not enough data to accurately train the probability model underlying Xnet, making it necessary to complement it with a Bayesian prior. This obviously questions the applicability to datasets that significantly differ from the ones that served to tune the prior. Finally, Xnet does not provide a consensus chromatogram for each cluster: Its output is a CSV file that only assigns a cluster index to each line of the input CSV file.

### Objectives and contributions

The objective of this article is twofold: First is to propose a new cluster analysis pipeline adapted to the challenging problem of clustering multiplexed chromatographic profiles resulting from data independent acquisitions. The second objective is to build this pipeline around an algorithm which is not agglomerative and linkage-based. Concretely, we focused on *k*-means objective function, for two reasons: First, until recently, it was considered by the proteomics community as non-applicable to data as big as LC-MS data [7], while recent theoretical progresses have made this scaling-up possible [44] (this explains the historical predominance of agglomerative linkage-based clustering, less computationally demanding); Second, *k*-means can be reformulated to fit the reproducing kernel Hilbert space theory [45] (leading to the so-called kernel *k*-means framework [46]), which provides new opportunities to define similarity measures that capture the biochemical specificities of LC-MS data (a challenge that has consistently been pinpointed as essential over the last fifteen years [3, 5, 6, 11–13]).

The contributions of this article are the following: First, it introduces the use of Wasserstein-1 (W1) distance (a.k.a. earth mover’s distance, a.k.a. optimal transport distance) to account for similarities between elution profiles. Second, it shows that combining Nyström method and random Fourier features leads to adramatic data compression level that makes the *k*-means objective function minimizable on raw and high resolution proteomics data with a multi-core machine. Finally, it demonstrates the applicability and interest of the method to process proteomics data from DIA experiments.

## Methods

### Materials

To conduct our study, we have relied on three datasets. The first one, hereafter referred to as UPS2GT, is a publicly available dataset [23]. To be used as a benchmark for Xnet, this dataset had been preprocessed and manually annotated with isotopic envelopes that can serve as ground truth [47]. Moreover, the data had been converted into *centroid* mode, *i.e.* a compressed version of the original *profile* data. In the *profile* mode, each peak of the mass spectrum is represented by intensities reported for several consecutive *m*/*z* values, so as to account for the measurement imprecision. In contrast, the *centroid* mode summarises all the values of the profile mode into a single *m*/*z* value, located at the center of the measurement distribution. It leads to significantly smaller memory footprint, at the price of blurring the differences between true signal and noise.

The second dataset, hereafter referred to as Ecoli-DIA, is the raw output of a DIA analysis of an *Escherichia Coli* sample (containing over 15,000 peptides^1^ which signals are multiplexed). To avoid any distortion or information loss, it was stored using the profile mode. The resulting file has an important memory footprint of 3.6 GB. Thus, even though chromatogram clustering operates on fraction of the data only (the so-called MS1 acquisitions, see "Ecoli datasets: Data preparation"  section), it requires adapted software tools and methods.

Finally, to account for the rapid increment of data size in proteomics (resulting from using ever longer LC and ever more resoluted MS acquisitions), we have considered a third dataset, exactly similar to the Ecoli-DIA dataset, but acquired as Full-MS instead of as DIA. This means that 100% of the acquisition time was dedicated to MS1 signals, so as to mimick the extraction of a much larger DIA dataset resulting from more time- and *m*/*z*-resoluted acquisitions. This so-called Ecoli-FMS dataset has a memory footprint of 3.2 GB. Even though of equivalent size, this dataset is in fact 16 bigger than Ecoli-DIA (four times more MS1 spectra which are four times more resoluted), see "Ecoli datasets: Data preparation" section.

#### UPS2GT benchmark dataset

The UPS2GT dataset [47] resulted from the liquid chromatography coupled to mass spectrometry analysis of 48 human proteins of the Proteomics Dynamic Range Standard (UPS2) on a AB Sciex TripleTOF 5600 instrument using data dependent acquisition with an MS1 ion accumulation time of 250 ms [23].

The 28,568,990 detected points in the resulting LC-MS map were annotated according to their intensity value, either as informative or as noisy. Over 1,2 million informative points were segmented into 57,140 extracted ion chromatograms referred to as *traces*. Then, the traces were grouped into 14,076 isotopic envelopes. These envelopes constitute the dataset ground truth (therefore, the objective of the clustering task would be to re-build the envelopes from the traces). The final fully annotated data were stored in a CSV file, where each row depicts one LC-MS point with four pieces of information: its mass to charge ratio, retention time, intensity, trace label and envelope label. The points that were assumed noise were given -1 or 0 as trace label.

#### Ecoli datasets: wet-lab analysis

*Escherichia Coli* bacteria were lysed with BugBuster reagent (Novagen, final protein concentration 1$\upmu$g/$\upmu$L). Around 560 $\upmu$g of proteins were stacked in the top of a 4 - 12$\%$ NuPAGE ZOOM gel (Life Technologies) and stained with R-250 Coomassie blue. Gel was manually cut in pieces before being washed by six alternative and successive incubations in 25 mM NH$_4$HCO$_3$ for 15 min, followed by 25 mM NH$_4$HCO$_3$ containing 50$\%$ (v/v) acetonitrile. Gel pieces were then dehydrated with 100$\%$ acetonitrile and incubated with 10 mM DTT in 25 mM NH$_4$HCO$_3$ for 45 min at 56 $^\circ$C and with 55 mM iodoacetamide in 25 mM NH$_4$HCO$_3$ for 35 min in the dark. Alkylation was stopped by the addition of 10 mM DTT in 25 mM NH$_4$HCO$_3$ (incubation for 10 min). Gel pieces were then washed again by incubation in 25 mM NH$_4$HCO$_3$ followed by dehydration with 100$\%$ acetonitrile. Modified trypsin (Promega, sequencing grade) in 25 mM NH$_4$HCO$_3$ was added to the dehydrated gel pieces for incubation at 37 $^\circ$C overnight. Peptides were extracted from gel pieces in three sequential extraction steps (each 15 min) in 30 $\upmu$L of 50$\%$ acetonitrile, 30 $\upmu$L of 5$\%$ formic acid, and finally 30 $\upmu$L of 100$\%$ acetonitrile. The pooled supernatants were aliquoted and dried under vacuum.

The dried extracted peptides were resuspended in 5$\%$ acetonitrile and 0.1$\%$ trifluoroacetic acid and 500ng were analyzed by online nanoliquid chromatography coupled to tandem mass spectrometry (LC-MS/MS) (Ultimate 3000 RSLCnano and the Q-Exactive HF, Thermo Fisher Scientific). Peptides were sampled on a 300 $\upmu$m 5mm PepMap C18 precolumn (Thermo Fisher Scientific) and separated on a 75 $\upmu$m 250 mm C18 column (Reprosil-Pur 120 C18-AQ, 1.9 $\upmu$m, Dr. Maisch HPLC GmbH). The nano-LC method consisted of a 120 minute multi-linear gradient at a flow rate of 300 nl/min, ranging from 5 to 41$\%$ acetonitrile in 0.1$\%$ formic acid. The spray voltage was set at 2 kV and the heated capillary was adjusted to 270$^\circ$C. For the Ecoli-FMS dataset, survey full-scan MS spectra (*m*/*z* from 400 to 1,400) were acquired with a resolution of 240,000 after the accumulation of $3\cdot 10^6$ ions (maximum filling time 200 ms). For the Ecoli-DIA dataset, survey full-scan MS spectra (*m*/*z* from 400 to 1,400) were acquired with a resolution of 60,000 after the accumulation of $3\cdot 10^6$ ions (maximum filling time 200 ms) and 30 successive DIA scans were acquired with a 33Th width and a resolution of 30,000 after the accumulation of $2\cdot 10^5$ ions (maximum filling time set to auto). The HCD collision energy was set to 30%. MS data were acquired using the software Xcalibur (Thermo Fisher Scientific).

#### Ecoli datasets: data preparation

The output of the LC-MS/MS experiments were converted from the proprietary RAW format into mzXML files using ProteoWizard [48]. It led to files of 11.4 GB (Ecoli-DIA) and of 10.2 GB (Ecoli-FMS), containing several pieces of information: discretized spectra under the form of coupled lists of *m/z* and intensity values; as well as metadata about the experiment (number of spectra, retention time range, etc).

**Fig. 1:**
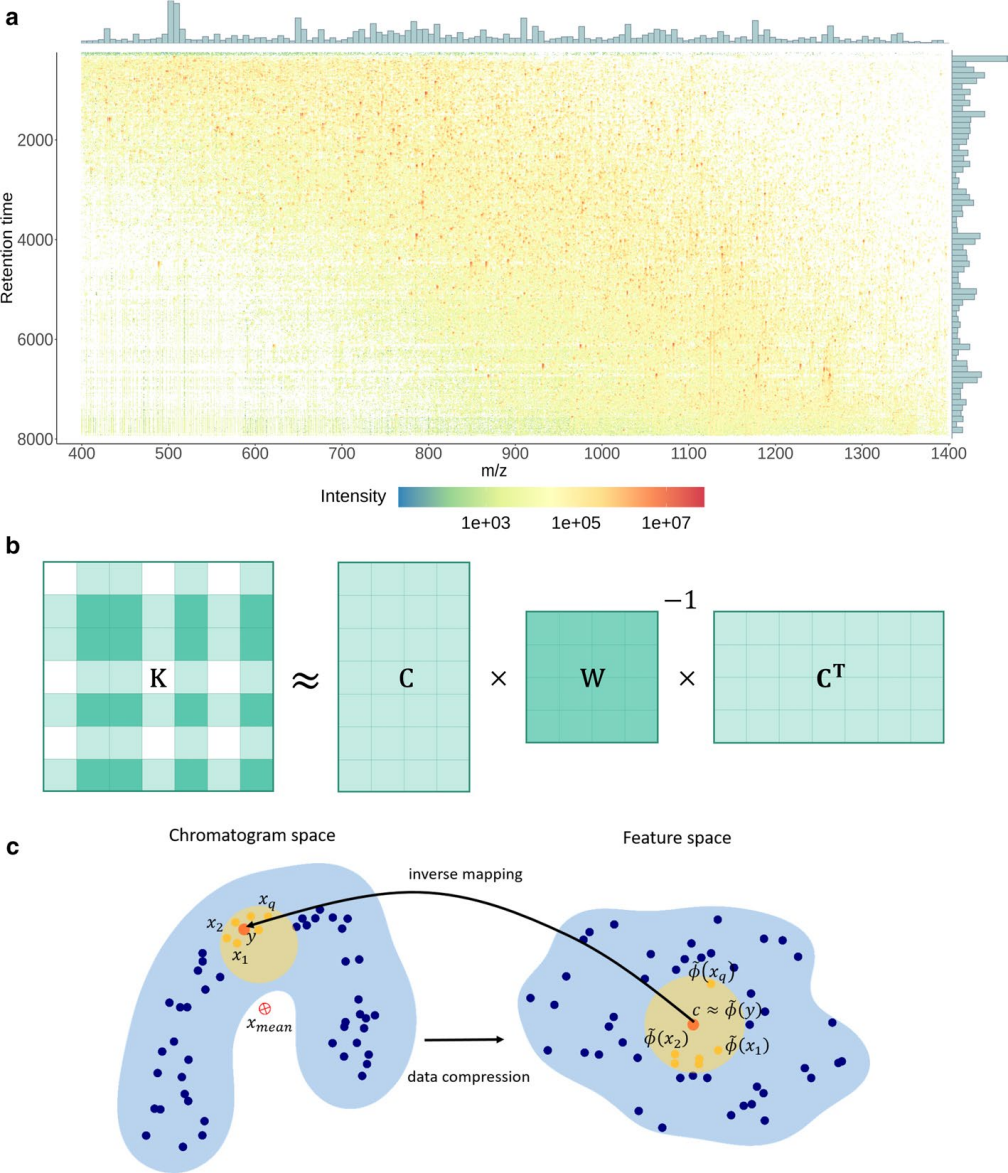
Data matrix, Nyström approximation and pre-image illustrations. **a** Ecoli-DIA data matrix. Each matrix column corresponds to a chromatographic profile for a fixed m/z value. Maximum Intensity for columns and for rows is depicted in bar plots. **b** Nyström kernel approximation. The matrix *C* represents the similarity between each data point and the random sample. The matrix *W* corresponds to the pairwise similarity evaluation between selected data points. **c** Pre-image problem. Consensus chromatogram construction amounts to solve a pre-image problem, *i.e.* to map the feature space (right) back to the space of chromatograms (left). Blue points depict the elution profiles (left) and their images in the feature space (right). The red points are the cluster centroid (right) and the corresponding consensus chromatogram (left). The yellow circles represent the cluster centroid and consensus chromatogram neighborhoods. Due to the mapping non-linearity, the mean chromatogram may lie outside the cluster, while the correct consensus chromatogram should belong to it

**Fig. 2:**
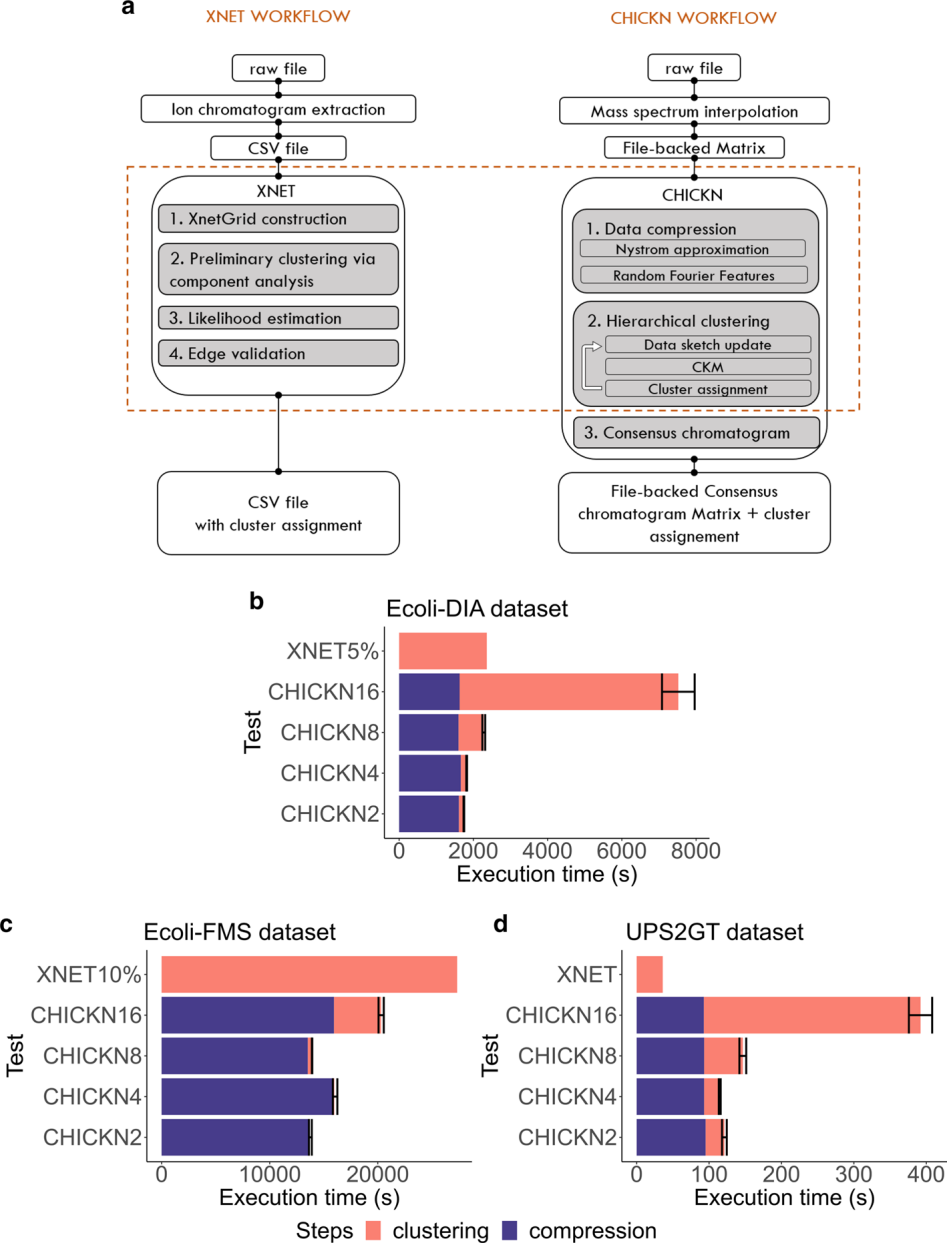
Xnet and CHICKN comparison. **a** The method workflows. To allow for fair comparisons, we have focused on the core algorithms, depicted within the dotted rectangle. **b**–**d** The execution time comparison for Ecoli and for the UPS2GT datasets. The CHICKN execution time is decomposed into the data compression time (blue) and the clustering time (pink). Note that XNet had to be run on 5% of the Ecoli-DIA dataset and 10% of the Ecoli-FMS dataset only, to avoid “out of memory” issues. The experiments on Ecoli-DIA were performed on a laptop, while other datasets were processed with a multi-core machine

In the case of the Ecoli-FMS dataset, all the spectra are peptide mass spectra, also termed MS1. However, the Ecoli-DIA datasets contains two types of spectra: precursor spectra (MS1) and fragmentation spectra (MS2). Thus, to work on the elution profiles, we have extracted the MS1 signals from the Ecoli-DIA file. Then, for both files, we have reconstructed chromatographic signals from MS1 spectrum intensities. As the proposed method aims to work on data as raw as possible (*i.e.* without preliminary denoising, smoothing and so on), we converted each mzXML file into an intensity matrix such as the ones of Fig. 1a (Ecoli-DIA) and of Additional file 1 (Ecoli-FMS), where each row corresponds to a spectrum and each column to an elution profile (despite possible *m/z* fluctuations that may hamper the signal continuity). We concretely constructed each data matrix using the LC-MS analysis time-stamps and a non-uniform sampling of the *m/z* range (see Additional file 2 for a detailed description). Concretely, the resampled *m/z* values are given by the following recursive formula:

1 $$\begin{aligned} m_{i+1} - m_i = \frac{0.015}{Res_{_{EXP}}} m_i^{\frac{3}{2}}, \end{aligned}$$

where $m_i$ is the $i\mathrm{th}$ sampled *m/z* value and $Res_{_{EXP}}$ is the instrument resolution used in the experiment ($Res_{_{FMS}}= 240,000$ and $Res_{_{DIA}}= 60,000$). Finally, we have linearly interpolated the intensity values at each node $m_i$ of the grid:

2 $$\begin{aligned} I_i = I_{\text {left}}+(m_i - m_{\text {left}}) \cdot \frac{I_{\text {right}} - I_{\text {left}}}{m_{\text {right}} - m_{\text {left}}}, \end{aligned}$$

where *m* and *I* pairs with sub-indexes “$\text {left}$”, “$\text {right}$” refer the left and right neighboring peaks. This is followed by the deletion of the few empty columns. The resulting Ecoli-DIA data matrix is depicted in Fig. 1a: it contains around 3,300 rows and 190,000 columns and it has a footprint of 4.8 GB. As expected, the Ecoli-FMS data matrix (Additional file 1) is bigger: 14,000 rows, 700,000 columns and 82 GB. The bar plots in the margins of both figures represent the intensity distribution across the matrix columns and rows. They show that the Ecoli-FMS and Ecoli-DIA matrices have the same structure and intensity range, despite different size.

### Methodology overview

The proposed methodology is composed of three consecutive parts, hereafter detailed:

1. *Profile similarity definition*
  As frequently discussed in the literature [3, 5, 6, 11–13], the choice of a similarity measure that reflects the biochemical semantics of LC-MS data is essential to achieve efficient processing. In this article, we relied on *Wasserstein-1 distance* [49, 50, 51] (or W1, detailed in the “Metric choice” section) and we transformed it into a similarity by applying a negative exponential function: If $x_i$ and $x_j$ are two chromatograms (or columns from the data matrix), their similarity thus reads:
  3 $$\begin{aligned} k(x_i,x_j) = e^{-\gamma \cdot [d_{W_1}(x_i,x_j)]^p} \end{aligned}$$
  where $d_{W_1}$ is the W1 distance and where $\gamma$ is a neighborhood parameter, which tuning authorizes up/down scaling the similarity values. The use of a similarity measure of the form of a negative exponential of a distance is convenient, since it makes it possible to apply the *kernel trick* [52] (see "Kernel-trick"  section), *i.e.* to apply a machine learning algorithm as if it were operating in a so-called *feature space* (depicting a non-linear data transform which respects the semantic of the chosen similarity measure).
2. *Data compression*
  Applying the kernel trick can be rather computationally demanding: For a dataset of size *N*, it requires the computation of a kernel (or similarity) matrix of size $N \times N$. Thus, with between $10^5$ and $10^6$ chromatograms in the Ecoli datasets, computing and storing the kernel matrix is simply not tractable. The purpose of *Nyström method* [53] (see "Nyström approximation" section) is to replace the kernel matrix by a low rank approximation, as illustrated in Fig. 1b. By relying only on the similarities between each data element and a randomly selected subset, it provides a dramatic reduction of the computational burden at the price of a small and controlled loss of accuracy. Even though Nyström approximation allows for an efficient computation of the kernel matrix, it does not accelerate the clustering algorithm itself, which requires multiple traversing of the entire dataset (*i.e.* *N* elements). To cope for this, it has recently been proposed in the compressive learning framework [54] to summarize the entire dataset by a relatively small vector of fixed size, referred to as *data sketch*, and to have the algorithm operating on his sketch only, irrespective of the original data. Concretely, we built the data sketch as an average of *random Fourier features* of the chromatographic profiles in the feature space (see "Random Fourier feature sketching" section).
3. *Cluster and centroid definitions*
  Lloyd algorithm [55] (*i.e.* the most classical algorithm to cluster data according to the *k*-means objective function) cannot directly be applied on sketched data. Fortunately, it is possible to rely on the *Compressive* *k*-*means* (CKM) algorithm proposed in [56] (see "Cluster computations"  section). However, CKM only returns a set of cluster centroids and does not cluster the data *per se*. Therefore, traversing the entire (original) dataset to perform the *assignment* of each chromatogram to its closest centroid (according to the W1 distance) is necessary (see "Cluster assignment"  section). CKM complexity does not depend on the original data size (as it operates on the data sketch) which makes it well-scalable. However, its complexity grows rapidly with the number of clusters, which is an issue as thousands of clusters can be sought in LC-MS data. To cope for this, we implemented a *hierarchical clustering scheme*, where each cluster is recursively divided into a small number of sub-clusters until the desired number of clusters is obtained (see "Cluster assignment"  section). This procedure provides a set of clusters with centroids only defined in the feature space. To recover the corresponding consensus chromatograms, one has to solve a *pre-image problem*. We practically did so by computing the mean of the elution profiles neighboring each centroid (see "Pre-image computation"  section).

To the best of the authors’ knowledge, this work is the first one to combine Nyström method and compressive learning with random Fourier features on a problem as difficult as the clustering of LC-MS data, which combines high-dimensionality and a very large number of potential clusters in addition to the traditional difficulties of raw biological data (non-linearities, low signal-to-noise ratio, *etc.*). From this point on, we refer to the proposed method as CHICKN (standing for Chromatogram HIerarchical Compressive K-means with Nyström approximation).

### Profile similarity definition

#### Metric choice

Originally, the Wasserstein-1 (W1) metric was defined to compute optimal transport strategies, which explains why it is also referred to as the *earth mover’s distance*. It has witnessed a recent gain of interest in machine learning as an efficient way to measure a distance between two probability distributions [57, 58]: Essentially, if one sees probability distributions as earth heaps, the most energy efficient way to move one earth heap in place of the other makes an interesting distance estimate. In this work, we leveraged a similar analogy between an earth heap and a chromatographic elution profile. Concretely, this approach is insightful since it accounts for two distinct components of what makes chromatographic elution profiles similar or not: their time separation as well as their difference of shape. Let us also note that this distance has recently been applied to LC-MS data, yet, to spectra rather than to chromatograms [51].

**Fig. 3:**
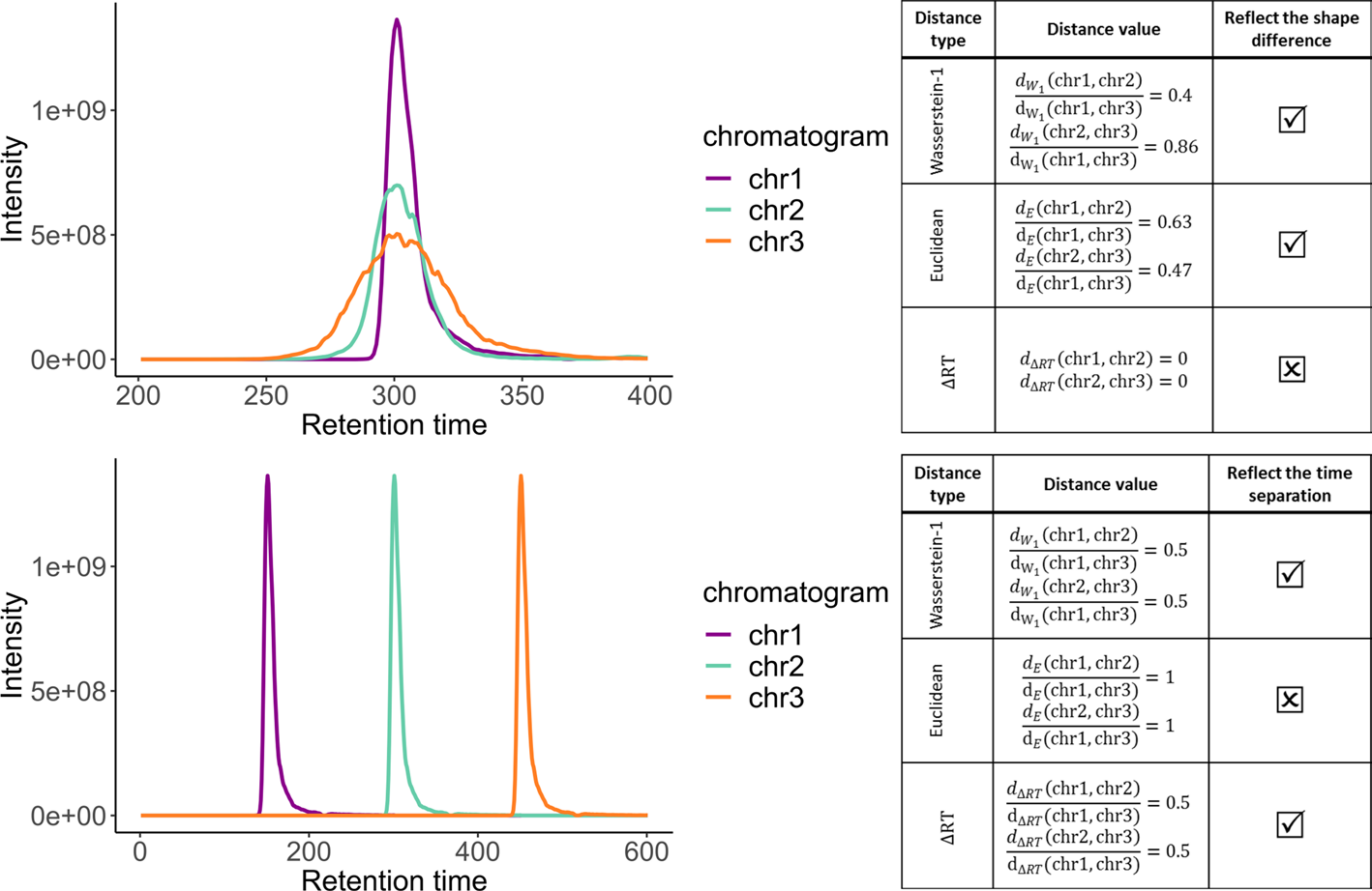
Distance metrics for chromatographic data analysis. Comparison of Wasserstein-1, Euclidean and RT difference distances on real chromatographic profiles from the Ecoli-FMS dataset

**Fig. 4:**
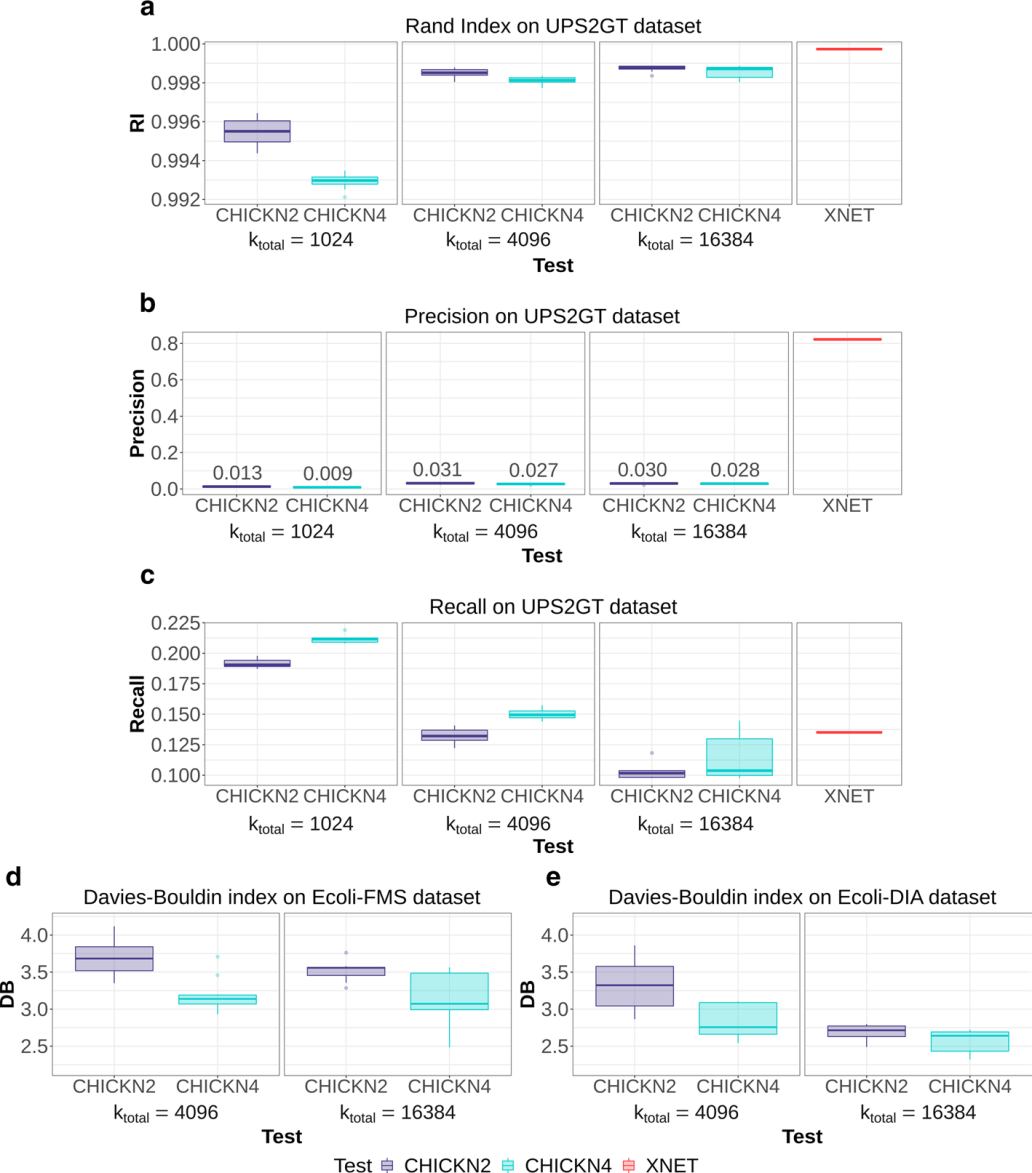
Statistical result analysis. **a** Rand index, **b** Precision, **c** Recall and **d**–**e** DB index depending on the *k* and $k_{\text {total}}$ parameters; CHICKN2 and CHICKN4 tests are depicted in purple and light blue respectively; For the UPS2GT dataset, additional comparisons with Xnet (in red) are provided

In general, the W1 distance between distributions $\mathcal P$ and $\mathcal Q$ is computed by solving Kantorovich minimization problem, namely:

4 $$\begin{aligned} d_{W_1}(\mathcal P,\mathcal Q) = \inf \limits _{\xi \in \mathcal J (\mathcal P, \mathcal Q)} \int \Vert x- y\Vert d\xi (x, y) \end{aligned}$$

where $\mathcal J (\mathcal P, \mathcal Q)$ denotes all joint distributions $\xi (x,y)$ that have marginals $\mathcal P$, $\mathcal Q$. However, in the 1-dimensional discrete setting where distributions $\mathcal P$ and $\mathcal Q$ are replaced by chromatograms $x = (x^1, \dots , x^n)$ and $y = (y^1, \dots , y^n) \in \mathbb {R}^{n}$, the W1 distance boils down to a difference between empirical cumulative functions:

5 $$\begin{aligned} d_{W_1}(x,y) = \sum _{j=1}^n |F_{x}(j) - F_{y}(j)|, \end{aligned}$$

where $F_x(j) = \sum \limits _{i \le j} \frac{x^i}{\sum \limits _{k=1}^n x^k}$ is the $j\mathrm{th}$ component of the cumulative distribution function of chromatogram *x*.

#### Kernel trick

Converting distances between data vectors into similarities by means of a negative exponential function is a good way to derive a similarity measure endowed with the positive semi-definite (or PSD) property.^2^ This property is essential to the application of the kernel trick [59], which notably explains why kernels of the form $k(x_i,x_j) = e^{-\gamma \cdot [d_2(x_i,x_j)]^p}$, with $p=1$ (the Laplacian kernel) or $p=2$ (the Gaussian kernel) and with $d_2$ depicting the Euclidean distance are classically used.

Concretely, let $X = [x_1, \dots , x_N] \in \mathbb {R}^{n\times N}$ be the data matrix composed of *N* chromatograms. The kernel trick actually consists in using the similarity measure to implicitly map the data onto a feature space that better represents them. The mapping is deemed “implicit” as it does not require the computation of coordinates of the data point images $\Phi = [\phi (x_1), \dots , \phi (x_N)]$, where $\phi$ denotes the mapping function. Two conditions must be met for this trick to work: First, the algorithm must rely on similarity measures only (*i.e.* once the similarities are computed, the values of the $x_i$’s are not used any more). Second, the similarity measure reproduces the inner product of the feature space: $k(x,y) = \left<\phi (x), \phi (y)\right>$. According to Mercer’s theorem [60], any PSD similarity measure satisfies the second condition. From that point on, we refer to $K = \Phi ^T\Phi = \left[ k(x_i, x_j)\right] _{i,j =1, \dots , N}$ as the *kernel matrix*.

However, when using a distance like $d_{W_1}$, which does not derive from a norm inducing an inner product on the data space (like for instance $d_2$), then the PSD-ness is not guaranteed [61]. In this work, we have investigated both the Laplacian W1 and the Gaussian W1 kernels: While we exhibit a formal proof of the Laplacian W1 kernel PD-ness (see Additional file 3, Section 3), we only have empirical evidence in the Gaussian case (see Additional file 3, Section 2). As in practice, both kernels lead to similar ranks in pairwise similarities, the resulting clusters only marginally differ. Owing to its popularity in life science applications, as well as to its easier tuning (interpretation and stability of the hyperparameter) the article thus focuses on the Gaussian case. Notably, as computational costs are necessarily higher with $p=2$ than $p=1$, the displayed runtimes are an upper bound for both cases. However, for qualitative analysis, results with $p=1$ are also depicted in various additional files (see below).

**Fig. 5:**
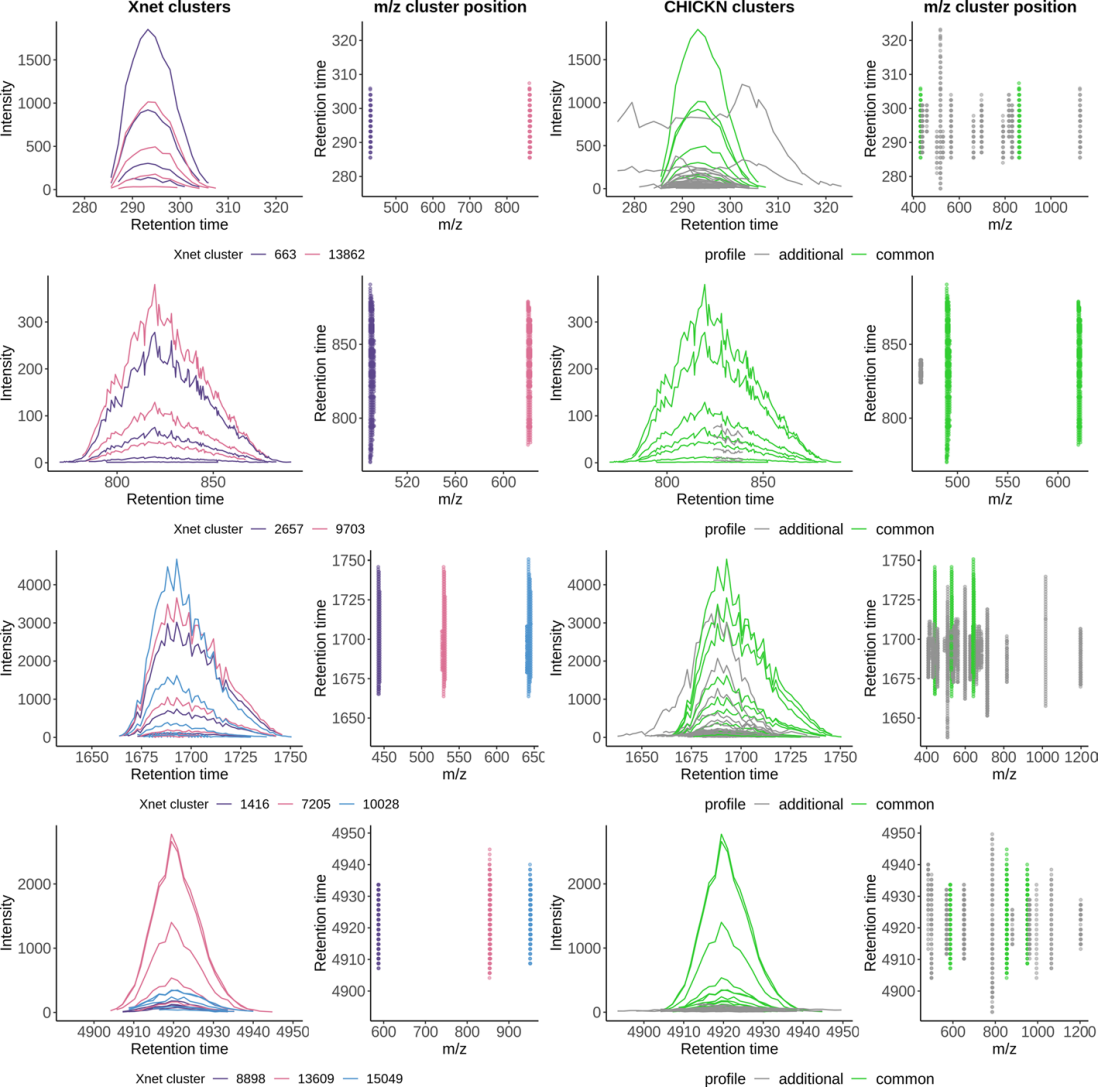
Xnet and CHICKN clusters for UPS2GT dataset. Each of the four lines represent a series of chromatograms in the context of their Xnet and CHICKN Cluster. On the plot of the leftmost column, a series of chromatograms with similar shapes are represented in different colors (2 or 3) according to the distinct Xnet clusters they belong to. In the second column, each elution profile is represented with the same color, according to its *m*/*z* position, hereby illustrating that Xnet clusters similar signals in different clusters because of a too large *m*/*z* difference. The plot of the third column represents the CHICKN cluster which encompasses all the Xnets cluster profiles of the leftmost column (in green), as well as other signals (in gray) falling in the same CHICKN cluster, hereby illustrating CHICKN builds meaningful patterns irrespective of the *m*/*z* information that is essential to isotopic envelope construction. In the rightmost column, the *m*/*z* positions of the signals of the third columns, depicited with the same color code

**Fig. 6:**
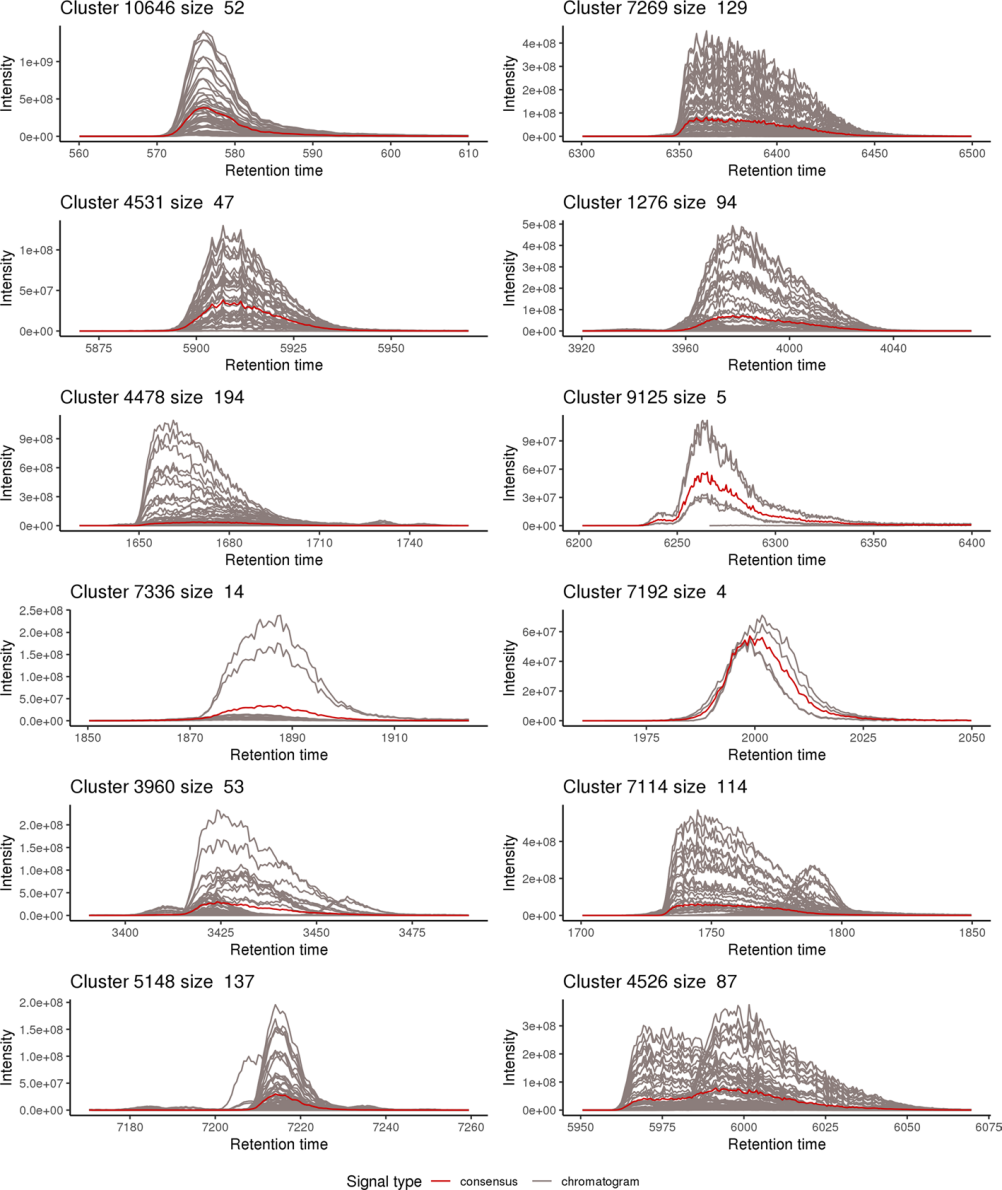
Examples of well-formed clusters for the Ecoli-FMS dataset. 12 clusters proposed by CHICKN (represented as time series), where each chromatogram is represented in gray, and where the consensus chromatogram is represented in red. The numbers above each example indicate the cluster ID and the number of chromatograms it encompasses

### Data compression

#### Nyström approximation

Brute force computation of a kernel matrix has a quadratic complexity, so that it does not easily scale-up. To cope for this, a classical solution is to apply Nyström approximation. This approach relies on the fast decaying property of the kernel spectrum (the set of kernel matrix eigenvalues): the smallest eigenvalues of the kernel matrix can safely be removed (intuitively, alike principal component analysis). Concretely, one approximates the kernel matrix ${K} \in \mathbb {R}^{N\times N}$ as following:

6 $$\begin{aligned} {K} \approx C W^{-1} C^\top , \end{aligned}$$

with $C={K}P \in \mathbb {R}^{N \times l}$ and $W=P^\top {K}P \in \mathbb {R}^{l \times l}$, where $P \in \mathbb {R}^{N \times l}$ is constructed from an $N \times N$ identity matrix where $(N-l)$ randomly selected columns are removed. The larger *l*, the better the approximation, but the heavier the computations. Finally, according to [53], an additional rank-*s* truncated singular value decomposition (SVD) is of interest to increase numerical stability. This leads to Algorithm 1, which complexity^3^ is $\mathcal {O}(N\cdot n\cdot l + N\cdot l^2)$. 
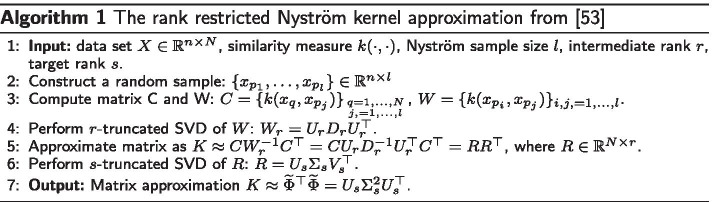


It provides the following approximation of the kernel matrix: $K \approx \widetilde{\Phi }^\top \widetilde{\Phi }$ where the matrix $\widetilde{\Phi }= \left[ \widetilde{\phi }(x_1), \dots , \widetilde{\phi }(x_N)\right]$ is obtained by applying the feature mapping $\widetilde{\phi }(x_i) = (\lambda _1 u_{1i}, \dots , \lambda _s u_{si})$, where $\lambda _j$ and $u_{ji}, j=1, \dots , s$ and $i=1, \dots , N$ are the *s* highest eigenvalues and eigenvectors (columns of matrix $U_s$) of *K* (see Algorithm 1). Moreover, it is demonstrated in [62] that the approximation accuracy is guaranteed when Nyström sample size *l* is on the order of $\sqrt{N}$. It was also shown in [53] that the target dimension *s* scales to $\mathcal O(\sqrt{l\cdot k})$, where *k* is the number of clusters, and the intermediate rank *r* is equal to $\frac{l}{2}$.

#### Random Fourier feature sketching

The sketching procedure of [54] is closely related to random Fourier features [63] , which seminal idea is to rely on Bochner’s theorem [64] to approximate any shift-invariant (*i.e.* $k'(x,y) = \kappa (x - y)$) PD kernel (by leveraging the fact it is a Fourier transform of some non-negative measure $\upmu$):

7 $$\begin{aligned} k'(x,y) =\mathbb E_{w \sim \mu }\left( e^{-i w^\top (x- y)}\right) . \end{aligned}$$

Elaborating on this, [54] proposed to apply a similar random Fourier map

8 $$\begin{aligned} \varphi (x) = \frac{1}{\sqrt{m}} \left[ e^{-iw_j^\top x}\right] _{j=1}^m, \end{aligned}$$

(where Fourier frequencies $w_1, \dots , w_m$ are randomly sampled from some distribution $\Omega$) and to average it over all data points to approximate the data distribution itself, instead of the kernel. Concretely, applying $\varphi (\cdot )$ onto the Nyström extended data $\widetilde{\Phi }$ (that is $Z = [\varphi (\widetilde{\phi }(x_1)), \dots , \varphi (\widetilde{\phi }(x_N))] \in \mathbb C ^{m \times N}$), led us to computing the data sketch as:

9 $$\begin{aligned} SK(\widetilde{\Phi }) = \frac{1}{N\sqrt{m}}\left[ \sum \limits _{i=1}^N e^{-i w_j^\top \widetilde{\phi }(x_i)}\right] _{j=1}^m \in \mathbb C^{m} \end{aligned}$$

The critical step of this data compression method lies in the frequency distribution estimation. It has been empirically shown in [54] that $\Omega = \mathcal N (0, \frac{1}{\sigma ^2}{} \mathbf{I} )$ is a suitable choice for it mimicks well the fast decaying property of real life signals. Then, $\sigma ^2$ can be estimated from a small data fraction using nonlinear regression. Applying this frequency distribution law allows to promote more informative sketch components and to eliminate small sketch values, which are usually related to noise. The key computational benefit of the compression is the independence between the data sketch length *m* and the data size *N*: *m* should be of the order of $k\cdot s$ [54], where *s* is the target dimension in Nyström approximation and *k* is the number of clusters.

### Cluster and centroid definitions

#### Cluster computations

CKM (the compressive implementation of the *k*-means clustering presented in [56]) can be used to compute the cluster centroids from the data sketch $SK(\widetilde{\Phi })$ introduced in Eq. (9). Briefly, and in contrast with classical Lloyd’s algorithm, it is a greedy heuristic based on orthogonal matching pursuit, which searches for a data representation as a weighted sum of cluster centroids by minimizing the difference between corresponding sketches:

10 $$\begin{aligned} \Vert SK(\widetilde{\Phi }) - \sum \limits _{i=1}^k \alpha _i SK(c_i)\Vert _2^2 \end{aligned}$$

The CKM involves two main steps summarized in Algorithm 2. First, across several iterations, it alternates between expanding the cluster centroid set with a new element, whose sketch is the most correlated to the residue; and recomputing the centroid weights using non-negative least-squares minimization. The second step consists in the global minimization of (10) with respect to cluster centroids and their weights. 
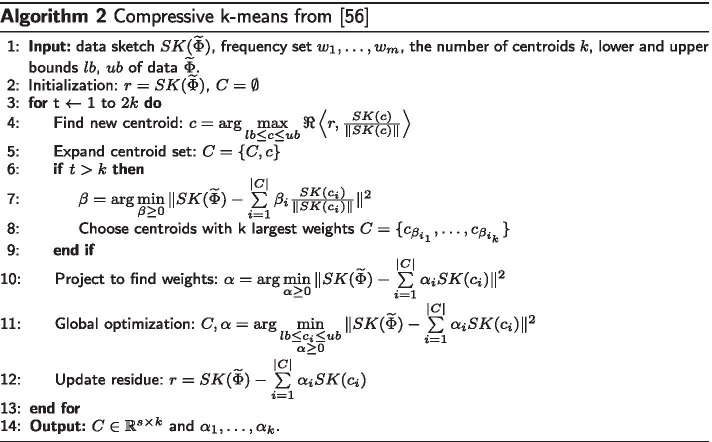


#### Cluster assignment

The CKM algorithm only provides the cluster centroids and does not assign data points to clusters. Nevertheless, this can be achieved afterwards by finding the centroid which has the highest similarity value to each data point. Concretely, a cluster centroid *c* in the feature space can be defined using Nyström extension as follows:

11 $$\begin{aligned} c \approx \widetilde{\phi }(y) = \Sigma _s^{-1} U_s^T k_c \end{aligned}$$

where *y* is a cluster centroid in the input (chromatograms) space, and where $k_c = \left[ k(x_1, y), \dots , k(x_N,y)\right]$ is an unknown vector of similarities between *y* and all given chromatograms. The columns of matrix $U_s$ contain *s* eigenvectors of *K* corresponding to its *s* highest eigenvalues (the diagonal matrix $\Sigma _s$). The estimation of $k_c$ can be achieved by minimizing the difference between *c* and $\widetilde{\phi }(y)$:

12 $$\begin{aligned} \min _{y \in \mathbb R^{n}} \left\| \Sigma _s^{-1} U_s^T k_c - \frac{c}{\Vert c\Vert }\right\| ^2 \end{aligned}$$

The importance of the normalization term in (12) has been highlighted in [65] as an energy-preserving term to balance Nyström approximation. The solution of (12) can be found using the Moore-Penrose pseudo-inverse:

13 $$\begin{aligned} k_c \approx U_s \Sigma _s \frac{c}{\Vert c\Vert } \approx \widetilde{\Phi }^T \frac{c}{\Vert c\Vert }. \end{aligned}$$

Finally, the chromatographic profile $x_i, \ i = 1, \dots , N$ is associated to cluster *j* if

14 $$\begin{aligned} c_j = \arg \max \limits _{c\in \{c_1, \dots , c_k\}} \left<\widetilde{\phi }(x_i), \frac{c}{\Vert c\Vert } \right>\end{aligned}$$

The most important CKM feature is its constant execution time regardless of the data size. However, its computational complexity grows cubically with the number of clusters, so that it is not realistic to process LC-MS data where tens of thousands of clusters are classically expected. To cope for this, a divisive hierarchical scheme can be instrumental: Starting from a small number of clusters, one iteratively splits each cluster into *k* sub-clusters until a sufficiently large number of clusters $k_\text {total}$ is achieved. However, this strategy requires, for each independent call of the clustering algorithm, an update of the data sketch as well as a complete assignment to clusters. Thus, to practically improve its computational efficiency, we leveraged the expected decrease of the cluster size at each iteration to optimize the code, and we decided to compute all the data sketches from the same frequency samples, either on the entire dataset (at first step) or on the cluster to be re-clustered (at the following iterations). Finally, it appeared these repetitive computations of the cluster sketches and assignments did not hamper the efficiency of the whole process.

#### Pre-image computation

The combination of Nyström approximation and of random Fourier features leads to an additional difficulty: To recover the signal of each consensus elution profile, it is necessary to compute its reverse mapping from the feature space back to the input space. This is referred to as a *pre-image* problem and it is ill-posed: only an approximation of the cluster centroids in the input space can be obtained. The conventional fixed point iteration method [66] cannot be applied due to the use of the W1 distance. Similarly, the reconstruction of a consensus chromatogram as the mean of the cluster chromatograms is not adapted, due to large scale non-linearities between the input and feature spaces, as illustrated in Fig. 1c.

To correct for this, we decided to compute a local (*i.e.* small-scale) mean by considering only a subset of the closest chromatograms. To determine the cluster centroid neighbourhood $\mathcal {N}(c)$, we proceeded similarly to the cluster assignment step, by choosing the chromatograms in the cluster $\mathcal {J}(c)$ with the highest similarities to the cluster centroid:

15 $$\begin{aligned} \mathcal {N}(c) = \{x_1, \dots , x_q\} \subset \mathcal {J}(c) \mid k(c, x) > k(c, y) \quad \forall x \in \mathcal {N}(c), y \in \mathcal {J}(c) \setminus \mathcal N (c), \end{aligned}$$

where similarities $k(c,\cdot )$ were estimated using Eq. (13). Concretely, $\mathcal {N}(c)$ was defined by selecting the *q* closest neighbors (so that $q= |\mathcal {N}(c)|$). The tuning of parameter *q* is discussed with that of other parameters in the “Parameter tuning” section.

### Performance metrics

For experiments annotated with a ground truth (like UPS2GT dataset), clustering accuracy can be evaluated with the Rand index (RI). The Rand index measures the percentage of correctly clustered pairs of signals over the total number of pairs. Let us denote as $U = \{U_1, \dots , U_k\}$ the obtained clusters and as $V = \{V_1, \dots , V_q\}$ the ground truth clusters. A pair of signals is considered as correctly clustered: *true positive (TP)* or *true negative (TN)*, if signals are assigned to the same cluster in *U* and *V* or on the contrary, to different clusters in *U* and *V*. A pair of signals is called *false positive (FP)* (resp. *false negative (FN)*), if signals are grouped in *U* (resp. *V*) but not in *V* (resp. *U*). Then, the Rand index is given by:

16 $${\text {RI}} = \frac{TP + TN}{TP + TN + FP + FN}$$

The maximum value of the Rand index is 1 (perfect match with the ground truth). Additionally, it is possible to evaluate how often different chromatograms are grouped in the same cluster; and how often similar chromatograms were assigned to different clusters. To do so, one classically relies on the Precision and Recall metrics, respectively:

17 $${\text{Precision}} = \frac{{TP}}{{TP + FP}}\quad {\text{Recall}} = \frac{{TP}}{{TP + FN}}$$

For datasets without ground truth annotation (like both Ecoli datasets), it is possible to rely on the Davies - Bouldin (DB) index. Let us denote as $\mathcal J(c_j)$ the $j^{th}$ cluster with the cluster centroid $c_j$, and as $\left\{ \mathcal J(c_1), \dots , \mathcal J(c_k)\right\}$ the set of obtained clusters. The within cluster distance reads:

18 $$\begin{aligned} S_j = \frac{1}{|\mathcal {J}(c_j)|} \sum \limits _{x_i \in \mathcal {J}(c_j) } d_{W_1}(x_i, c_j) \end{aligned}$$

The DB index is defined through the ratio of the within cluster distances to the between cluster distance $d_{W_1}(c_i, c_j)$:

19 $$\begin{aligned} DB = \frac{1}{k} \sum \limits _{i=1}^k \max _{i\ne j} \frac{S_i + S_j}{d_{W_1}(c_i, c_j)}, \end{aligned}$$

It should be noted that the distance metric in the DB index and in the clustering algorithm must be the same, in our case the W1 distance in the original space. Moreover, the smaller the DB index, the better the clustering (as a good clustering minimizes cluster overlaps).

Finally, the computational load can easily be approximated by the recorded execution time, *i.e.* the difference between the end and start times, both of which being accessible in R with the Sys.time() function. For sake of brievety, execution times are reported for the Gaussian W1 kernel only, as Laplacian similarities are necessarily faster to compute (no squared distance to evaluate).

## Results

### Objectives of the experimental assessment

Many independent elements deserve evaluations: The first one is the practical interest of W1 distance in the context of LC-MS data. The second one is the computational load of our complete algorithm in function of the parameter tuning (on the one hand, an efficient compression technique is used; on the other hand, one targets the clustering of raw data into a high number of clusters, making its efficiency a challenge). The third one is the clustering result itself. However, a classical evaluation of the clustering performances will be of little interest: In fact, all *k*-means related algorithms (including their kernelized versions) have been extensively studied [44], so that their strengths and weaknesses are now well-documented. For instance, *k*-means optimizers can easily be trapped into local minima and cannot naturally deal with outliers, which are both significant drawbacks; however, they scale up well to very-high dimensional data, which definitely is an asset for LC-MS applications. In contrast, highlighting the differences of our approach with respect to linkage-based agglomerative clustering and showing that despite noticeable differences, one obtains clusters which are meaningful, is of real practical interest to computational mass spectrometry experts.

As reported in the “Background” section, comparisons with Xnet is mandatory. However, considering the reported specificities (trace extraction preprocessing, envelope assumption simplification, *etc.*), comparing Xnet and CHICKN workflows may appear as somewhat arbitrary. To cope for this, we have made the following choices: First, we have focused on the core of each algorithm, as represented in Fig. 2a. Second, we have adapted the UPS2GT and Ecoli datasets to be processed by each algorithm: The UPS2GT data are already formatted into a CSV file meeting Xnet requirements. To construct a data matrix suitable to CHICKN from the UPS2GT data, we simply loaded the data points according to their retention time and trace labels in the matrix columns (similarly to Xnet, we excluded point with trace indices -1 and 0, as assumed to be noise). This led to a data matrix containing 57,140 columns and 6,616 rows. Conversely, to build the CSV files from Ecoli datasets, we stored any non-zero entry of the data matrix in a row, the column index being used in place of the trace labels.

### Wasserstein distance validation

W1 distance was proposed to discriminate between signals that represent different elution profiles. To assess this choice, we compared it with two distances amongst the most widely used in mass spectrometry signal processing: The first one is the classical Euclidean distance. The second one is the peak retention time difference (or $\Delta$RT): It corresponds to the difference between the time stamps at which each signal reaches its highest intensity value. Based on the Ecoli-FMS dataset (which provides the finest temporal sampling), we examined two situations presented in Fig. 3: In the first one, we selected 3 signals with different shapes, that we precisely aligned so that their pairwise $\Delta$RT was zero; in other words, only the shape difference makes it possible to discriminate them. Conversely, in the second situation, an elution profile was translated to mimic a case where only the $\Delta$RT was meaningful. In both situations, the second chromatogram (chr2) stands as an in-between the first (chr1) and the third chromatogram (chr3). As illustrated by the distance ratios given in the tables embedded in Fig. 3, both the Euclidean and the $\Delta$RT distances are meaningful in one case: The Euclidean distance captures the shape information, while $\Delta$RT captures the time translation effect. However, none of these classically used distances is able to capture both the shape and the translation simultaneously. On the contrary, W1 distance is efficient on both situations, making it a suitable distance to construct a similarity measure adapted to LC-MS data.

### Parameter tuning

Unlike Xnet, CHICKN is governed by eight parameters. Four of them are involved in the data compression: Nyström sample size (*l*), target rank (*s*), kernel parameter ($\gamma$) and sketch size (*m*). Three parameters are involved in the hierarchical clustering: number of clusters at each iteration of the hierarchical clustering (*k*), upper bound of the total number of expected clusters ($k_{\text {total}}$) and maximum number of levels in the hierarchy (*T*). The remaining parameter is the neighbourhood size in the consensus chromatogram computation (*q*). However, all parameters except $\gamma$ and *q* are interrelated (see the “Data compression” section as well as  [53, 62]) and can be expressed through *k*, $k_{\text {total}}$ and *N* (the dataset size) as follows:

20 $$\begin{aligned} \begin{aligned} l&\approx \sqrt{N}, \\ s&\approx \sqrt{k} \cdot N^{1/4},\\ m&\approx k^{3/2}\cdot N^{1/4}, \\ T&= \lfloor \log (k_{\text {total}}, k)\rfloor . \end{aligned} \end{aligned}$$

These theoretical results can nonetheless be discussed. Notably, tuning the sketch size *m* to a larger value may be of interest if contrarily to our case, the computational efficiency is not the only targeted goal. Thus, we have performed complementary investigation to relate the clustering performance (in terms of DB index) to the sketch size (see Additional file 4, leftmost figure). Oddly enough, it appears the DB index increases (*i.e.* the performances deteriorates) when the sketch size increases (leading to a more refined representation of the data). However, it appears to be an indirect consequence: when increasing *m*, more differences between the signals are represented, making it possible to define a larger number of smaller clusters (see Additional file 4, rightmost figure).

Finally, four parameters remain ($\gamma$, *q*, *k* and $k_{\text {total}}$). Concretely, we tuned the kernel parameter $\gamma$ as an average of the power of *p* distances to the $\nu$ nearest neighbors for all chromatographic profiles:

21 $$\begin{aligned} \gamma = \frac{1}{N\cdot \nu } \sum _{i =1}^N \sum _{j = 1}^{\nu } [d_{W_1}(x_i, x_{i_j})]^p, \end{aligned}$$

where $x_{i_1}, \dots , x_{i_{\nu }}$ are $\nu$ neighbors of $x_i$ (selected among the *l* points of the Nyström sample) and $p \in \{1,2\}$ depending on the kernel type. Practically, we observed that tuning $\nu$ to 32 guaranteed each data point to be sufficiently connected to the rest of the dataset, as advised in [37]. Moreover, we observed that $\gamma$ was rather stable with respect to $\nu$, for both Laplacian W1 and Gaussian W1 kernels. However, as expected, the stability is higher with the latter than with the former (see Additional file 5).

For *q* (in the consensus chromatogram computation) we observed that the shape cluster problem (see “Pre-image computation” section) could only occur with significantly large clusters (few tenth of elements). Thus, as preliminary stability analysis indicated us that the consensus chromatogram shapes were preserved across various values of $\nu$ (see Additional file 6), we decided to bound *q* with $\nu$ and to set $q = \min (\nu , \text {cluster size})$.

A known drawback of *k*-means objective function is the requirement to set the maximum number of expected clusters (knowing some clusters can remain empty). In our case, this is achieved by tuning *k* and $k_{\text {total}}$. Yet, it should be noted that increasing *k* leads to decreasing *T* for a fixed value of $k_{\text {total}}$ so that a trade-off between *T* and *k* must be sought. With this respect, we have evaluated different scenarios with $k =$ 2, 4, 8 and 16. CHICKN execution times (excluding the data compression step, which remains constant whatever the various scenario) on the smallest (UPS2GT) and largest (Ecoli-FMS) datasets are depicted in Additional file 7. This experiment pointed out the importance of tuning *k* to a small enough value, which is coherent with the observation that the original CKM algorithm does not scale up well with the number of clusters. Practically, working with $k=2$ or 4 appeared to be the most efficient.

In the case of UPS2GT, the expected number of isotopic envelopes is known (*i.e.* 14,076). Thus, it is easy to tune $k_{\text {total}}$ accordingly (*i.e.* $2^{14}=4^7=16,384$). However, knowing that CHICKN does not rely on the envelope assumption simplification, it can be expected to find a much lower number of clusters: broadly, all the isotopic envelopes corresponding to different charge states of a same peptide can be expected to cluster together. Therefore, it also makes sense to tune $k_{\text {total}}$ to $4^5=1,024$; *i.e.* close enough from the expected number of identifiable peptides in the sample (around 700, according to [23]).

Tuning $k_{\text {total}}$ for any real life data (*i.e.* unlabeled) is much more complicated. However, the *Escherichia Coli* sample is well studied, and based on prior biological/analytical knowledge, 15,000 different peptides can be expected, broadly. Consequently, for both Ecoli datasets, $k_{\text {total}}=16,384$ seems reasonable. Finally, even though it is not as sensible from a biological viewpoint, we have decided to also consider $k_{\text {total}}=4^6= 4,096$, which provides an even ground for computational load comparisons (see next section for details).

To summarize, three different ways to tune $k_{\text {total}}$ are insightful: 1024 for the UPS2GT dataset only (as it matches the number of expected peptides); 4096 on all datasets (for computational benchmarcks); and 16,384 on all datasets (number of isotopic envelopes in UPS2GT and number of expected peptides in Ecoli datasets).

Finally, we fixed the remaining parameter values using the formulas in Eq. (20), as summarized in Table 1.

**Table 1:** Summary of the different combinations of parameter tuning

Dataset	$\gamma$	*l*	*s*	*m*	*k*	*T*		
						$k_{\text {total}} = 1,024$	$k_{\text {total}} = 4,096$	$k_{\text {total}} = 16,384$
UPS2GT	5.96e−06	240	22	44	2	10	12	14
	6.9e−06	240	31	124	4	5	6	7
Ecoli-DIA	9.06e−06	432	30	60	2	–	12	14
	9.27e−06	432	42	168	4	–	6	7
Ecoli-FMS	7.07e−07	863	42	84	2	–	12	14
	7.03e−07	863	59	236	4	–	6	7

### Computational load

We have compared the execution times of CHICKN and Xnet cores (see Fig. 2a). Previously reported comparisons showed us that CHICKN execution time largely depends on *k*. However, it only has a sub-linear complexity with respect to $k_{\text {total}}$: As illustrated in Additional file 8, multiplying $k_{\text {total}}$ by 4 only results in a threefold (resp. twofold) increase in the CHICKN run-time for the Ecoli-FMS (resp. UPS2GT) dataset. As reducing $k_{\text {total}}$ to limit the execution time will therefore be of little interest, experiments hereafter reported only focused on the influence of *k*. Despite CHICKN being more efficient when run with $k = 2$ and 4 (see “Parameter tuning” section), we also included comparisons with $k = 8$ and 16 to investigate the consequences of sub-optimal parameter tuning. The corresponding tests are referred to as CHICKN2, CHICKN4, CHICKN8 and CHICKN16. Therefore, to rely on an even basis for comparisons, we focused on $k_{\text {total}}=4,096$: it is a power of 16, contrarily to 1024 and 16,384 (which are even not a power of 8).

Since CHICKN algorithm embeds a compressive *k*-means algorithm which may converge towards different local minima depending on the stochasticity of several steps, each scenario was repeated 10 times and the average execution time was reported. In contrast, Xnet being deterministic, it was executed once. In [17], Xnet exhibits impressive computational times on pre-processed and adequately formatted data. However, raw LC-MS data stored in a matrix format are more cumbersome. Thus, our first experiment was to compare the efficiency of Xnet and of CHICKN on the Ecoli-DIA dataset, using a laptop machine with the following characteristics: HP Pavilion g6 Notebook PC with Intel(R) Core(TM) i5-3230M CPU @ 2.60 GHz, 8 Gb of RAM, 4 cores, running under Ubuntu 18.04.4 LTS OS. Xnet produced an “out-of-memory” error when trying to cluster more than 10,000 columns (*i.e.* 5% of the Ecoli-DIA dataset) in a single batch. This is why Fig. 2b compares the computational time of CHICKN2, CHICKN4, CHICKN8 and of CHICKN16 on the entire Ecoli-DIA dataset to that of Xnet on only 5% of the same dataset. On this figure, different colors are used to discriminate between the clustering step *per se* and CHICKN preliminary data compression step. Let us note that the compression step is time consuming, however, it also includes the computations of all the W1 similarities. This as-a-matter-of-factly illustrates the computational cost of relying on more elaborated metrics to capture the semantics of data as complex as LC-MS ones. Except for CHICKN16, which has already been pointed as suboptimal, CHICKN is always faster for a dataset 20 times larger.

This first experiment clearly showed CHICKN could be used on a simple laptop, even with large datasets, in long but acceptable times (half an hour to two hours, broadly). Then, to reduce the execution times of our multiple experiments, but also to allow Xnet working on a larger dataset, we moved to a larger station using 10 cores of an Intel Xeon CPU E5-2470 v2 @ 2.40 GHz, 94 GB of RAM and running with CentOS Linux release 7.4.1708. As depicted in Fig. 2c, on such a machine, CHICKN was able to process Ecoli-FMS within 5h30 (most of them being necessary to perform the preliminary compression), despite its huge size. On the contrary, with the same machine, Xnet only processed 10% of it in a comparable time (almost 8 hours). Moreover, larger fractions of the dataset were not processable, as leading to memory failure.

To explain this discrepancy, we noticed that Xnet spent a considerable time to construct the preliminary network. The nature of Ecoli data (raw data without any trace pre-processing and recorded with the highly resoluted *profile* mode, see “Materials” section) contrasts with that of UPS2GT, on which Xnet is really efficient. As it appears on Fig. 2d, CHICKN is clearly not as fast as Xnet to process UPS2GT: The Xnet analysis took less then 40 s, while CHICKN computation times varied from 2 to 7 min depending on values of parameter *k* (from 2 to 16).

As a whole, these experiments illustrate the utmost importance of prior preprocessing methods when studying LC-MS data. In this context, algorithms working on raw data, such as CHICKN, are real assets.

### Cluster evaluation

Figure 4 reports the Rand index, Precision and Recall (UPS2GT dataset) as well as the DB index (Ecoli datasets) with different clustering strategies: CHICKN2 and CHICKN4 (with $k_{\text {total}} \in \{1,024\ ; 4,096\ ; 16,384\}$ and with $p=2$), as well as Xnet (on UPS2GT only, for computational reasons). A similar figure for $p=1$ is available in Additional file 9.

First, it can be noted that the Rand index is hardly informative (Fig. 4a): All clustering methods exhibit an index of almost 1, and it is necessary to go three (and sometimes four) decimals to notice a difference. Such high values are a direct consequence of the huge number of expected clusters in UPS2GT datasets, which comes with an excessively large number of true negative pairs (almost 99 $\%$ of all possible pairs). In this context, the Rand index obtained with “only” 1024 expected clusters is particularly highlighting: Despite 16 times less clusters, it achieves an equivalent index. This indicates that, relatively, the provided clustering is probably of better quality.

However, contrarily to the Rand index, Precision and Recall are informative to compare with Xnet, as the true negative pair count does not level the scores. With this regard, it clearly appears on Fig. 4b that the Precision is incomparably better with Xnet. Although foreseeable (ground truth with 14,076 envelopes whereas CHICKN sought a thousand of peptides), this requires a deeper analysis: Concretely, Xnet tends to over-cluster (which artificially improves the Precision index), as it provided 17,153 clusters covering 93% of the dataset (7% of the elution profiles are excluded by Xnet) where the ground truth labels proposed only 14,076 of them (on 100% of the dataset). In addition, Xnet priors were trained on the same UPS2GT dataset as for evaluation, so that high performance are expectable. With this regard, it is particularly noteworthy that the Recall (Fig. 4c) varies the other way around. Concretely, it is best for CHICKN4 with $k_{\text {total}}=1,024$ despite this number being completely different from the one derived from the ground truth. In addition to be in line with our observations on the Rand index, this concurs with the peptide-level knowledge of the dataset: CHICKN was supposed to group together differently charged peptides, which it did (see Additional files 10 and 14 as well as below), as it provided only 510 (CHICKN4)/ 740 (CHICKN2) clusters on the entire UPS2GT dataset, hereby leaving 300 to 500 empty clusters^4^; and leading to a number of clusters in line with the expected number of peptides in the sample. Overall, the differences between Xnet and CHICKN on UPS2GT seem to be more related to the difference of objectives (finding isotopics envelopes *vs.* finding peptide-related clusters), as already discussed. Interestingly, this interpretation is confirmed by the Ecoli dataset experiments.

In absence of ground truth for both Ecoli datasets, we chose the tuning minimizing the DB index (see Fig. 4d, e): $k_{\text {total}}= 16,384$ for Ecoli-FMS and for Ecoli-DIA. With such a tuning, we obtained around 11,600 (resp. around 9,400) non-empty clusters for Ecoli-FMS (resp. Ecoli-DIA). This number is obviously lower than the expected number of identifiable peptides (between 15 and 20 thousands), however under-clustering was clearly supported by empirical observations (see above, as well as Additional file 4, rightmost figure). This clearly means that CHICKN could not separate too many peptides with too similar elution profiles. However, this can be easily explained by the difference of complexity between the UPS2GT and the Ecoli samples: while the former is fairly simple (a handful of spiked proteins), the latter ones are complex real life samples for which the discriminative power of the liquid chromatography is clearly challenged (as illustrated in the next section). This is notably why fragmentation spectra are classically used to identify as many as 15 to 20 thousand peptides. However, achieving to discriminate half of this number of peptides with MS1 processing only is noticeable.

Finally, let us note, that, in general, relying on $k=4$ provided slightly better scores. We assume that $k=4$ was a trade-off between cluster diversity ($k>4$) and computational efficiency ($k=2$), as discussed above.

## Discussions

### Cluster interpretability

Beyond evaluation metrics, it is insightful to compare algorithms according to the interpretability of the clusters they can provide. Figure 5 represents different elution profiles from UPS2GT (their shape as well as their *m*/*z* position) in the context of the clusters they fall into, according to CHICKN and Xnet. The envelope assumption simplification clearly appears: As expected, Xnet splits into different clusters elution profiles that are arguably similar for the reason they have too different *m*/*z* values. In contrast, CHICKN promotes the inner coherency of clusters as it aggregates related Xnet clusters together. Notably, Additional files 10 and 14 show a subset of 12 clusters provided by CHICKN, each gathering at least 2 differently charged ions from a same peptide (all of them being identified and manually validated with the associated MS2 spectra). Interestingly, the multiple isotopes of each ion also appear to be grouped, as illustrated by the manifold of profile co-clustered with each ion. Morevoer, a refine analysis of CHICKN clusters shows that, globally, they contain similar chromatograms, which is coherent both with the clustering metrics provided above, and with the expected behavior of the W1 kernel. However, some clusters also contain noise signals, as for examples, the first two lines of Fig. 5. Although undesirable, this is a direct consequence of (*i*) the grouping capabilities of CHICKN, which captures similarities between slightly different but largely overlapping signals (third line); and (*ii*) the possibility to run CHICKN on raw data, which also contains many spurious signals that need be spread across various meaningful clusters.

Similar conclusions regarding CHICKN behavior can be derived from the Ecoli datasets (let us focus on the Ecoli-FMS one, as it displays elution profile signals with higher sampling resolution, due to the Full-MS acquisition). The majority of clusters (Fig. 6 for the Gaussian W1 kernel and Additional file 12, for the Laplacian W1 one) containing high intensity signals depicts meaningful consensus chromatograms, as well as similar profiles even though corresponding to different *m*/*z* values. However, we observed that some clusters could be separated into several sub-clusters to improve readability (see Additional file 13). It could intuitively be interpreted as the necessity to increase $k_{total}$. However, two observations goes against this: First, from a signal viewpoint, as the phenomenon mainly impacts lower intensity profiles, it also highlights the difficulty of finding consensus patterns near the noise level, which equally affects most of the clustering algorithms. In this context, over-clustering is usually not considered a viable solution. Second, from an analytical viewpoint, the clustering algorithm cannot be expected to separate beyond the chromatographic capabilities (as in Additional file 13, where few different profiles have too important overlap to expect discrimination).

Finally, it is worthy focusing on consensus chromatograms: interestingly enough, most of those observed in Fig. 6 and in Additional file 12 have meaningful shapes that are not deteriorated by the presence of noisy signals in the cluster, which can be interpreted as a positive consequence of our method to compute the cluster centroids pre-image based on a restricted neighborhood (see “Pre-image computation” section).

### Implementation and code availability

CHICKN algorithm was implemented in R. The W1 distance computations and the gradient descent were accelerated using C and interfaced with R thanks to Rcpp. The data compression procedure and the hierarchical strategy were parallelized with RcppParallel, foreach and doParallel. To access and manipulate large data matrices, we relied on the File-backed Big Matrix class of the bigstatsr package [67]. A File-backed matrix allows to overcome the memory limitation by storing the data on the disk, using a binary memory-mapped file.

For practitioners, the proposed algorithm is available through an R package, available on Gitlab [68], as well as on the CRAN [69].

## Conclusion

We have presented two complementary contributions to the cluster analysis of LC-MS data. First, we have proposed a unique combination of hierarchical strategy, of Nyström approximation and of random Fourier features based compression technique to scale up the kernel *k*-means clustering to the large size, the large dimensionality and the large number of expected clusters of LC-MS data. Second, we have proposed to rely on the optimal transport framework (Wasserstein-1 distance) to define a similarity measure and we have shown it is insightful to capture the semantics of elution profiles in LC-MS data. On a more theoretical front, we have established the Wasserstein-1 distance could lead to a positive-definite Laplacian kernel, and exhibit a path for further investigations about a Gaussian one.

We have demonstrated these contributions could help extracting other structures than isotopic envelopes, even on multiplexed data acquired with Data Independent Acquisition protocol. However, the experimental assessment of these contributions is difficult to interpret. On the one hand, when compared to the canonical application of isotopic envelope extraction, CHICKN does not outperform the state-of-the-art algorithm (better Recall and worse Precision, as it tends to under-cluster rather than over-cluster). However, it provides an important advantage: it can be run on raw data and does not require costly preprocessing. As for an application-independent evaluation, it clearly appears that CHICKN is able to extract patterns from the data which are not accessible to linkage-based algorithms. Put together, we interpret this as following: Although cluster analysis has made important progresses in the theoretical front over the past 50 years, processing LC-MS data remains a challenge which requires research efforts. It is still necessary to propose complementary and differently principled algorithms that will help make LC-MS practitioners extract the best from their data. In this context, new kernels could be defined; and numerous state-of-the-art clustering algorithms recently developed in the machine learning community could advantageously be applied to LC-MS data.

## Supplementary Information

Below is the link to the electronic supplementary material.

**Additional file 1:** Ecoli-FMS data matrix. Figure depicting the matrix built thanks to the mass spectrum interpolation of Ecoli-FMS data. Each matrix column corresponds to a chromatographic profile for a fixed *m/z* value. Maximum Intensity for columns and for rows is depicted in bar plots.

**Additional file 2:** Preprocessing details. Detailed explanations of Eq. (1) (interpolation needs, justification of the method and parameter tuning).

**Additional file 3:** Kernel positive (semi-)definiteness. Empirical evidences (Gaussian W1 case) and formal demonstration (Laplacian W1 case) of the P(S)D-ness of the proposed kernels.

**Additional file 4:** Sketch size influence on the clustering. Influence of the sketch size on performances clustering of the Ecoli-DIA dataset, in function of the computational cost and the number of clusters.

**Additional file 5:** Kernel hyperparameter stability. Figure showing the stability of the hyperparameter γ of Laplacian and Gaussian W1 kernels with respect to the neighborhood maximum size ν.

**Additional file 6:** Consensus chromatogram stability. A set of 10 figures exemplifying the stability of the pre-image computation through the averaging of a neighborhood of varying size.

**Additional file 7:** Influence of *k* on the execution time of CHICKN. Figure depicting CHICKN execution time as a function of *k*, the number of clusters at each iteration, for both UPS2GT (blue) and Ecoli-FMS (red) datasets.

**Additional file 8:** Influence of *k*_total_ on the execution time of CHICKN. Figure depicting CHICKN execution time as a function of *k*_total_, the maximum number of clusters, for both UPS2GT (blue) and Ecoli-FMS (red) datasets.

**Additional file 9:** Performance evaluation for the Laplacian W1 kernel. This figure is the same as Fig. 4, yet with p = 1 instead of p = 2. The performances on the UPS2GT dataset are a bit lower than with the Gaussian W1 kernel (equivalent Rand index, better precision, lower recall), making it unable to compete with Xnet. However, on raw data such as Ecoli-DIA (i.e., on data CHICKN should work with), the Laplacian W1 kernel exhibit slightly better DB index than its Gaussian counterpart; however, this is hardly significant, making us conclude that strict performance should not be the criterion to choose the kernel.

**Additional file 10:** Differently charged ions of a same peptide tend to cluster together. A subset of clusters was manually inspected so as to label as many profiles with the corresponding identified ion. Although this labelling cannot be exhaustively conducted due to the largely incomplete coverage of MS/MS analysis, it could be established that ions of a same peptide cluster together in many cases.

**Additional file 11:** Cluster size distribution. Histograms of the cluster size distribution resulting from the application of CHICKN on each of the three datasets.

**Additional file 12:** Examples of well-formed clusters for the Ecoli-FMS dataset. Same figure as Fig. 6 with Laplacian W1 kernel.

**Additional file 13:** Examples of multiplexed clusters for the Ecoli-FMS dataset using CHICKN method. Figure illustrating that dividing multiplexed clusters into several sub-clusters would improve the elution profile interpretation. The real chromatograms and the consensus chromatograms are depicted in gray and in red, respectively.

**Additional file 14:** Differently charged ions of a same peptide tend to cluster together. Figure similar to Additional File 10. It depicts another subset of CHICKN clusters with chromatographic profiles manually annotated with the corresponding peptide ion. It could be established that ions of a same peptide tend to cluster together.

## Data Availability

The UPS2GT dataset supporting the conclusions of this article is available in the Github, https://github.com/optimusmoose/ups2GT [47]. The Ecoli DIA and Ecoli FMS raw data (generated and analyzed for the current study) are not publicly available due to their too large size. However they are available from the corresponding author upon reasonable request. To reproduce all experiments described in the article, the preposessed Ecoli datasets in the file-backed matrix format can also be provided upon request.

## Abbreviations

CHICKN: Chromatogram HIerarchical Compressive K-means with Nyström approximation
CKM: Compressive k-means
DB: Davies–Bouldin index
$\Delta$RT: Peak Retention Time difference
DIA: Data Independent Acquisition
Ecoli-DIA: Dataset resulting from the analysis of an *Escherichia coli* sample in DIA mode
Ecoli-FMS: Dataset resulting from the analysis of an *Escherichia coli* sample in FMS mode
FMS: Full mass spectrum (only MS1s are recorded)
FN: False Negative
FP: False Positive
LC-MS: Liquid chromatography and mass spectrometry
MS1: Peptide (or precursor) mass spectrum
MS2: Peptide fragment mass spectrum
*m/z*: Mass to charge ration
PD: Positive definite
PSD: Positive semi-definite
RI: Rand index
RT: Retention Time
SVD: Singular Value Decomposition
TN: True Negative
TP: True Positive
UPS2GT: Dataset resulting from the analysis of the Proteomics Dynamic Range Standard (UPS2) and manually annotated (ground truth)
W1: Wasserstein-1 distance

## Footnotes

1 We consider that a peptide is characterized by a triplet: its amino acid sequence, a list of post-translational modifications and their localization on the sequence. Accordingly, different isotope measurements can be grouped into a single peptide definition.

2 Positive semi-definiteness or PSD-ness, means the resulting similarity matrix will have only non-negative eigenvalues (if the eigenvalues are positive, the matrix is called positive definite or PD, see Additional file 3, Section 1).

3 As a recall, $\mathcal {O}(f(n))$ indicates that with an input data of size *n*, the running time will not exceed *C*.*f*(*n*) where *C* is a constant factor (*i.e*. independent of *n*).

4 More generally, the capability of CHICKN to adapt the cluster sizes to the data distribution is illustrated on Additional file 11.

## References

[1] Teleman J, Dowsey AW, Gonzalez-Galarza FF, Perkins S, Pratt B, Röst HL (2014). Numerical compression schemes for proteomics mass spectrometry data. Mol Cell Proteomics..

[2] Klaus B, Strimmer K (2013). Signal identification for rare and weak features: Higher criticism or false discovery rates?. Biostatistics..

[3] Tabb DL, MacCoss MJ, Wu CC, Anderson SD, Yates JR (2003). Similarity among tandem mass spectra from proteomic experiments: Detection, significance, and utility. Anal Chem..

[4] Tabb DL, Thompson MR, Khalsa-Moyers G, VerBerkmoes NC, McDonald WH (2005). MS2Grouper: Group assessment and synthetic replacement of duplicate proteomic tandem mass spectra. J Am Soc Mass Spectrom..

[5] Beer I, Barnea E, Ziv T, Admon A (2004). Improving large-scale proteomics by clustering of mass spectrometry data. Proteomics..

[6] Flikka K, Meukens J, Helsens K, Vandekerckhove J, Eidhammer I, Gevaert K (2007). Implementation and application of a versatile clustering tool for tandem mass spectrometry data. Proteomics..

[7] Frank AM, Bandeira N, Shen Z, Tanner S, Briggs SP, Smith RD (2008). Clustering millions of tandem mass spectra. J Proteome Res..

[8] Frank AM, Monroe ME, Shah AR, Carver JJ, Bandeira N, Moore RJ (2011). Spectral archives: Extending spectral libraries to analyze both identified and unidentified spectra. Nat Methods..

[9] Griss J, Foster JM, Hermjakob H, Vizcaíno JA (2013). PRIDE Cluster: building a consensus of proteomics data. Nat Methods..

[10] Griss J, Perez-Riverol Y, Lewis S, Tabb DL, Dianes JA, Del-Toro N (2016). Recognizing millions of consistently unidentified spectra across hundreds of shotgun proteomics datasets. Nat Methods..

[11] Falkner JA, Falkner JW, Yocum AK, Andrews PC (2008). A spectral clustering approach to MS/MS identification of post-translational modifications. J Proteome Res..

[12] Saeed F, Hoffert JD, Knepper MA (2014). CAMS-RS: Clustering algorithm for large-scale mass spectrometry data using restricted search space and intelligent random sampling. IEEE/ACM Trans Comput Biol Bioinf..

[13] The M, Käll L (2016). MaRaCluster: a fragment rarity metric for clustering fragment spectra in shotgun proteomics. J Proteome Res..

[14] Griss J, Perez-Riverol Y, The M, Käll L, Vizcaíno JA (2018). Response to “comparison and Evaluation of Clustering Algorithms for Tandem Mass Spectra”. J Proteome Res..

[15] Wang L, Li S, Tang H (2019). MsCRUSH: fast tandem mass spectral clustering using locality sensitive hashing. J Proteome Res..

[16] Perez-Riverol Y, Vizcaíno JA, Griss J (2018). Future prospects of spectral clustering approaches in proteomics. Proteomics..

[17] Gutierrez M, Handy K, Smith R (2019). XNet: a Bayesian approach to extracted ion chromatogram clustering for precursor mass spectrometry data. J Proteome Res..

[18] Fischer B, Grossmann J, Roth V, Gruissem W, Baginsky S, Buhmann JM (2006). Semi-supervised LC/MS alignment for differential proteomics. Bioinformatics..

[19] Houel S, Abernathy R, Renganathan K, Meyer-Arendt K, Ahn NG, Old WM (2010). Quantifying the impact of chimera MS/MS spectra on peptide identification in large-scale proteomics studies. J Proteome Res..

[20] Chapman JD, Goodlett DR, Masselon CD (2014). Multiplexed and data-independent tandem mass spectrometry for global proteome profiling. Mass Spectrom Rev..

[21] Peckner R, Myers SA, Jacome ASV, Egertson JD, Abelin JG, MacCoss MJ (2018). Specter: linear deconvolution for targeted analysis of data-independent acquisition mass spectrometry proteomics. Nat Methods..

[22] Hu A, Lu YY, Bilmes J, Noble WS (2019). Joint precursor elution profile inference via regression for peptide detection in data-independent acquisition mass spectra. J Proteome Res.

[23] Tsou CC, Avtonomov D, Larsen B, Tucholska M, Choi H, Gingras AC (2015). DIA-umpire: comprehensive computational framework for data-independent acquisition proteomics. Nat Methods.

[24] Cox J, Mann M. MaxQuant enables high peptide identication rates, individualized p.p.b.-range mass accuracies and proteome-wide protein quantication. Nat Biotechnol. 2008;26(12):1367–72. 10.1038/nbt.1511 19029910

[25] Bertsch A, Gröpl C, Reinert K, Kohlbacher O. OpenMS and TOPP: open source software for LC-MS data analysis. In: Methods in molecular biology (Clifton, N.J.). vol. 696. Springer; 2011; 353–367. 10.1007/978-1-60761-987-1_23 21063960

[26] Bellew M, Coram M, Fitzgibbon M, Igra M, Randolph T, Wang P (2006). A suite of algorithms for the comprehensive analysis of complex protein mixtures using high-resolution LC-MS. Bioinformatics.

[27] Basu S, Davidson I, Wagstaff K (2008). Constrained clustering: advances in algorithms, theory, and applications.

[28] Sibson R (1973). SLINK: an optimally efficient algorithm for the single-link cluster method. Comput J.

[29] Defays D (1977). An efficient algorithm for a complete link method. Comput J..

[30] Ester M, Kriegel HP, Sander J, Xu X. A Density-Based Algorithm for Discovering Clusters in Large Spatial Databases with Noise. In: Proceedings of the 2nd International Conference on Knowledge Discovery and Data Mining. 1996; 96: 226–231.

[31] Michener SR. A statistical method for evaluating systematic relationships. Univ Kans Sci Bull. 1958;38:1409–1438. Available from: http://ci.nii.ac.jp/naid/10011579647/en/.

[32] Von Luxburg U, Williamson RC, Guyon I. Clustering: Science or art? In: Proceedings of ICML workshop on unsupervised and transfer learning; 2012; 65–79.

[33] Adolfsson A, Ackerman M, Brownstein NC (2019). To cluster, or not to cluster: an analysis of clusterability methods. Pattern Recogn.

[34] Datta S, Datta S (2003). Comparisons and validation of statistical clustering techniques for microarray gene expression data. Bioinformatics.

[35] Shi J, Malik J (2000). Normalized cuts and image segmentation. IEEE Trans Pattern Anal Mach Intell..

[36] Ng AY, Jordan MI, Weiss Y. On spectral clustering: Analysis and an algorithm. In: Advances in neural information processing systems; 2002; 849–856.

[37] Von Luxburg U (2007). A tutorial on spectral clustering. Stat Comput..

[38] Borges H, Guibert R, Permiakova O, Burger T (2019). Distinguishing between Spectral Clustering and Cluster Analysis of Mass Spectra. J Proteome Res..

[39] Cheng Y (1995). Mean shift, mode seeking, and clustering. IEEE Trans Pattern Anal Mach Intell..

[40] Comaniciu D, Meer P (2002). Mean shift: a robust approach toward feature space analysis. IEEE Trans Pattern Anal Mach Intell..

[41] Schubert E, Rousseeuw PJ. Faster k-Medoids clustering: improving the PAM, CLARA, and CLARANS algorithms. In: International conference on similarity search and applications. Springer; 2019; 171–187.

[42] Macqueen J. Some methods for classification and analysis. In: Proceedings of the Fifth Berkeley Symposium on Mathematical Statistics and Probability, Volume 1: Statistics. vol. 233. Oakland, CA, USA; 1967. p. 281–297. Available from: http://projecteuclid.org/bsmsp.

[43] Lloyd SP (1982). Least Squares Quantization in PCM. IEEE Trans Inf Theory..

[44] Jain AK (2010). Data clustering: 50 years beyond K-means. Pattern Recogn Lett..

[45] Williams CKI (2003). Learning with kernels: support vector machines, regularization, optimization, and beyond.

[46] Schölkopf B, Smola A, Müller KR (1998). Nonlinear component analysis as a Kernel eigenvalue problem. Neural Comput..

[47] Henning J, Tostengard A, Smith R (2019). A peptide-level fully annotated data set for quantitative evaluation of precursor-aware mass spectrometry data processing algorithms. J Proteome Res..

[48] Chambers MC, Maclean B, Burke R, Amodei D, Ruderman DL, Neumann S (2012). A cross-platform toolkit for mass spectrometry and proteomics. Nat Biotechnol..

[49] Yu Z, Herman G. On the earth mover’s distance as a histogram similarity metric for image retrieval. In: IEEE international conference on multimedia and expo, ICME 2005. 2005;2005(2):686–689.

[50] Courty N, Flamary R, Tuia D. Domain adaptation with regularized optimal transport. In: Joint European conference on machine learning and knowledge discovery in databases. Springer; 2014; 274–289.

[51] Majewski S, Ciach MA, Startek M, Niemyska W, Miasojedow B, Gambin A. The wasserstein distance as a dissimilarity measure for mass spectra with application to spectral deconvolution. In: 18th international workshop on algorithms in bioinformatics (WABI 2018). Schloss Dagstuhl-Leibniz-Zentrum fuer Informatik; 2018. .

[52] Schölkopf B. The kernel trick for distances. In: Advances in Neural Information Processing Systems; 2001; 301–307.

[53] Wang S, Gittens A, Mahoney MW (2019). Scalable kernel K-means clustering with Nyström approximation: relative-error bounds. J Mach Learn Res..

[54] Keriven N, Bourrier A, Gribonval R, Pérez P (2018). Sketching for large-scale learning of mixture models. Inf Inference J IMA..

[55] Hartigan JA, Wong MA (1979). Algorithm AS 136: a K-means clustering algorithm. Appl Stat..

[56] Keriven N, Tremblay N, Traonmilin Y, Gribonval R. Compressive K-means. In: ICASSP, IEEE international conference on acoustics, speech and signal processing - proceedings. Institute of Electrical and Electronics Engineers Inc.; 2017; 6369–6373.

[57] Givens CR, Shortt RM (1984). A class of Wasserstein metrics for probability distributions. Mich Math J..

[58] Gibbs AL, Su FE (2002). On choosing and bounding probability metrics. Int Stat Rev..

[59] Hofmann T, Schölkopf B, Smola AJ (2008). Kernel methods in machine learning. Ann Stat..

[60] Berlinet A, Thomas-Agnan C (2004). Reproducing Kernel Hilbert spaces in probability and statistics.

[61] Feragen A, Lauze F, Hauberg S. Geodesic exponential kernels: When curvature and linearity conflict. In: Proceedings of the IEEE conference on computer vision and pattern recognition; 2015; 3032–3042.

[62] Calandriello D, Rosasco L. Statistical and computational trade-offs in kernel K-means. In: Advances in neural information processing systems. vol. 2018-Decem; 2018; 9357–9367.

[63] Rahimi A, Recht B. Random features for large-scale kernel machines. In: Advances in neural information processing systems; 2008; 1177–1184.

[64] Puckette SE, Rudin W (1965). Fourier analysis on groups.

[65] Arias P, Randall G, Sapiro G. Connecting the out-of-sample and pre-image problems in Kernel methods. In: Proceedings of the IEEE computer society conference on computer vision and pattern recognition. IEEE; 2007; 1–8.

[66] Mika S, Schölkopf B, Smola A, Müller KR, Scholz M, Rätsch G. Kernel PCA and de-noising in feature spaces. In: Advances in neural information processing systems; 1999; 536–542.

[67] Prive F, Aschard H, Ziyatdinov A, Blum MGB (2018). Efficient analysis of large-scale genome-wide data with two R packages: Bigstatsr and bigsnpr. Bioinformatics..

[68] Permiakova O, Guibert R, Burger T. Gitlab of CHICKN (Chromatogram HIerarchical Compressive K-means with Nystrom approximation) R package; 2020. Available from: https://gitlab.com/Olga.Permiakova/chickn.

[69] Permiakova O, Guibert R, Burger T. CRAN repository of CHICKN (Chromatogram HIerarchical Compressive K-means with Nystrom approximation) R package; 2020. Available from: https://CRAN.R-project.org/package=chickn.

