# Supplementary figures and images for: CHICKN: extraction of peptide chromatographic elution profiles from large scale mass spectrometry data by means of Wasserstein compressive hierarchical cluster analysis
Source: BMC Bioinformatics. 2021 Feb 12;22:68. doi: 10.1186/s12859-021-03969-0 (PMC7881590; doi:10.1186/s12859-021-03969-0)

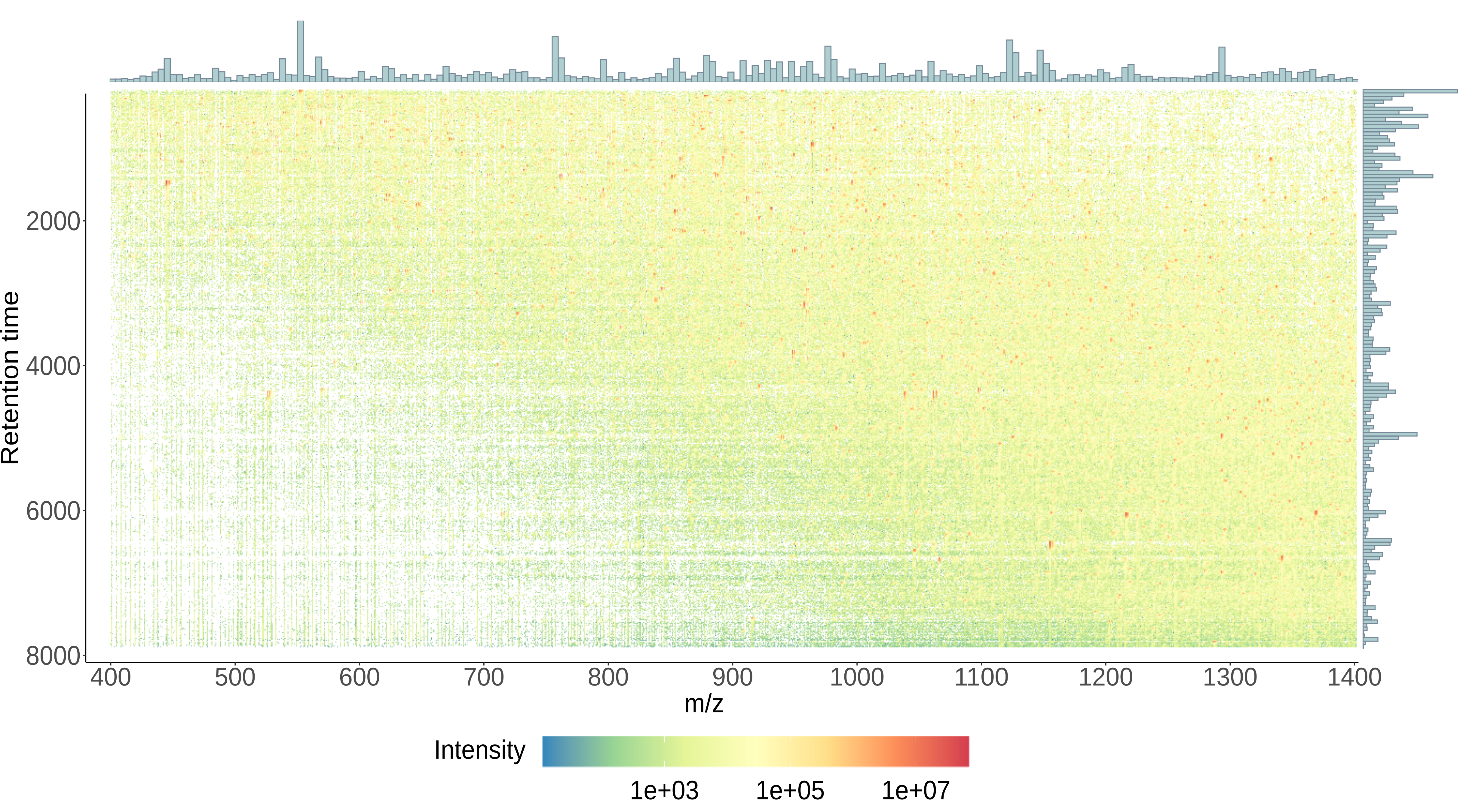

Supplement: Supplementary file 1 — Additional file 1: Ecoli-FMS data matrix. Figure depicting the matrix built thanks to the mass spectrum interpolation of Ecoli-FMS data. Each matrix column corresponds to a chromatographic profile for a fixed m/z value. Maximum Intensity for columns and for rows is depicted in bar plots. [file 12859_2021_3969_MOESM1_ESM.png]

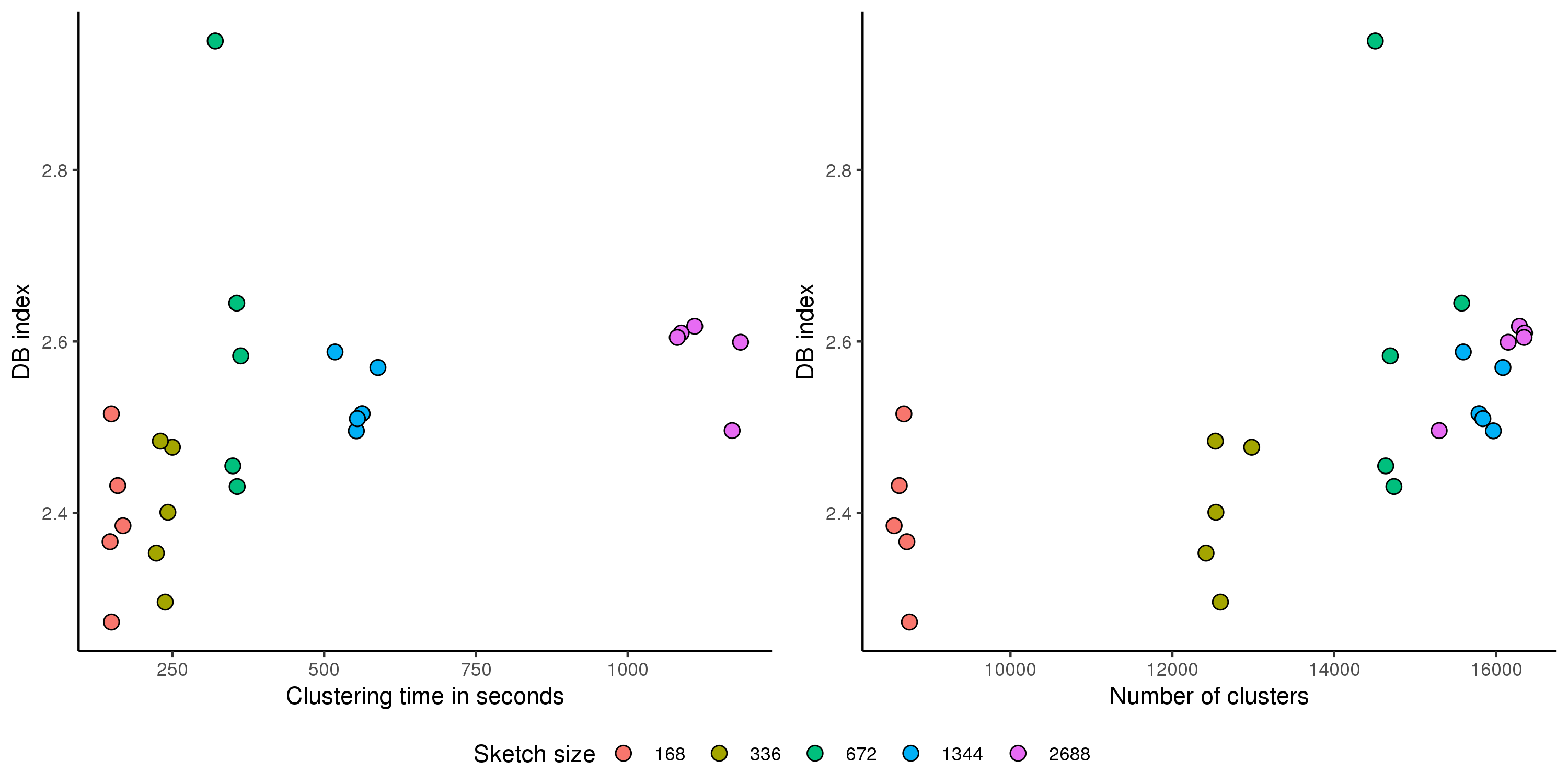

Supplement: Supplementary file 4 — Additional file 4: Sketch size influence on the clustering. Influence of the sketch size on performances clustering of the Ecoli-DIA dataset, in function of the computational cost and the number of clusters. [file 12859_2021_3969_MOESM4_ESM.png]

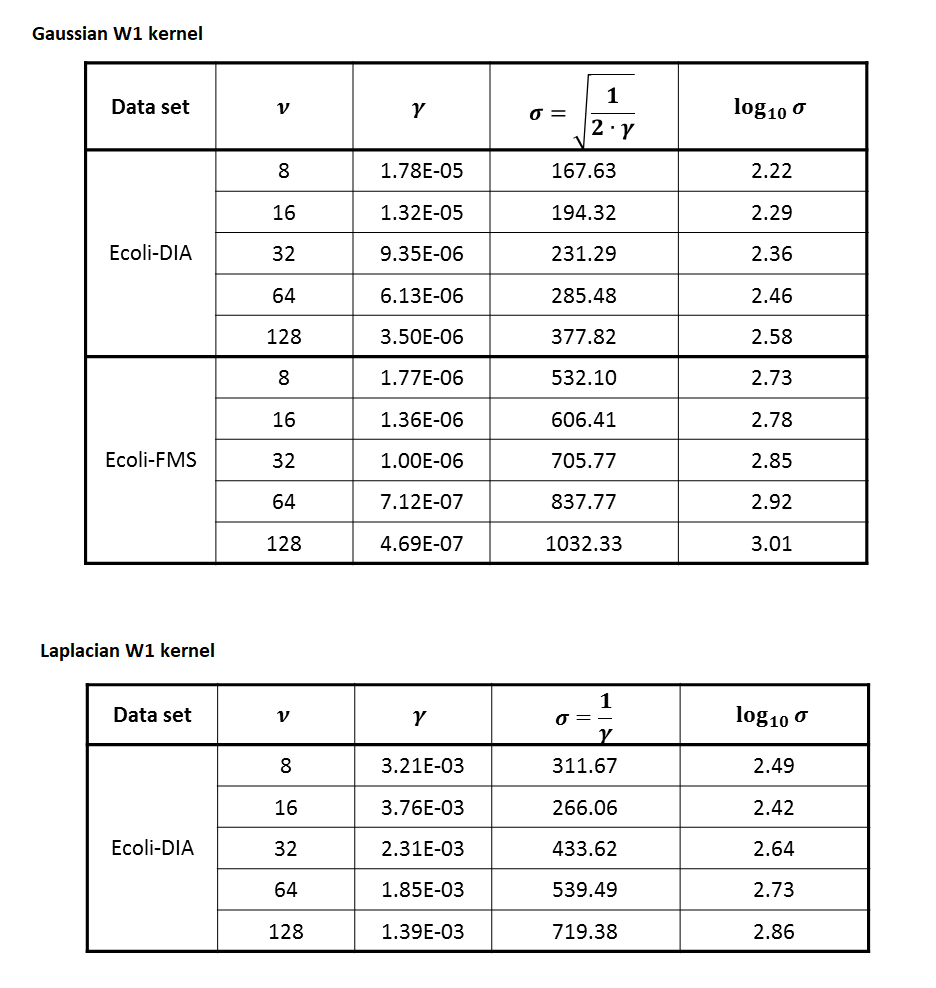

Supplement: Supplementary file 5 — Additional file 5: Kernel hyperparameter stability. Figure showing the stability of the hyperparameter γ of Laplacian and Gaussian W1 kernels with respect to the neighborhood maximum size ν. [file 12859_2021_3969_MOESM5_ESM.png]

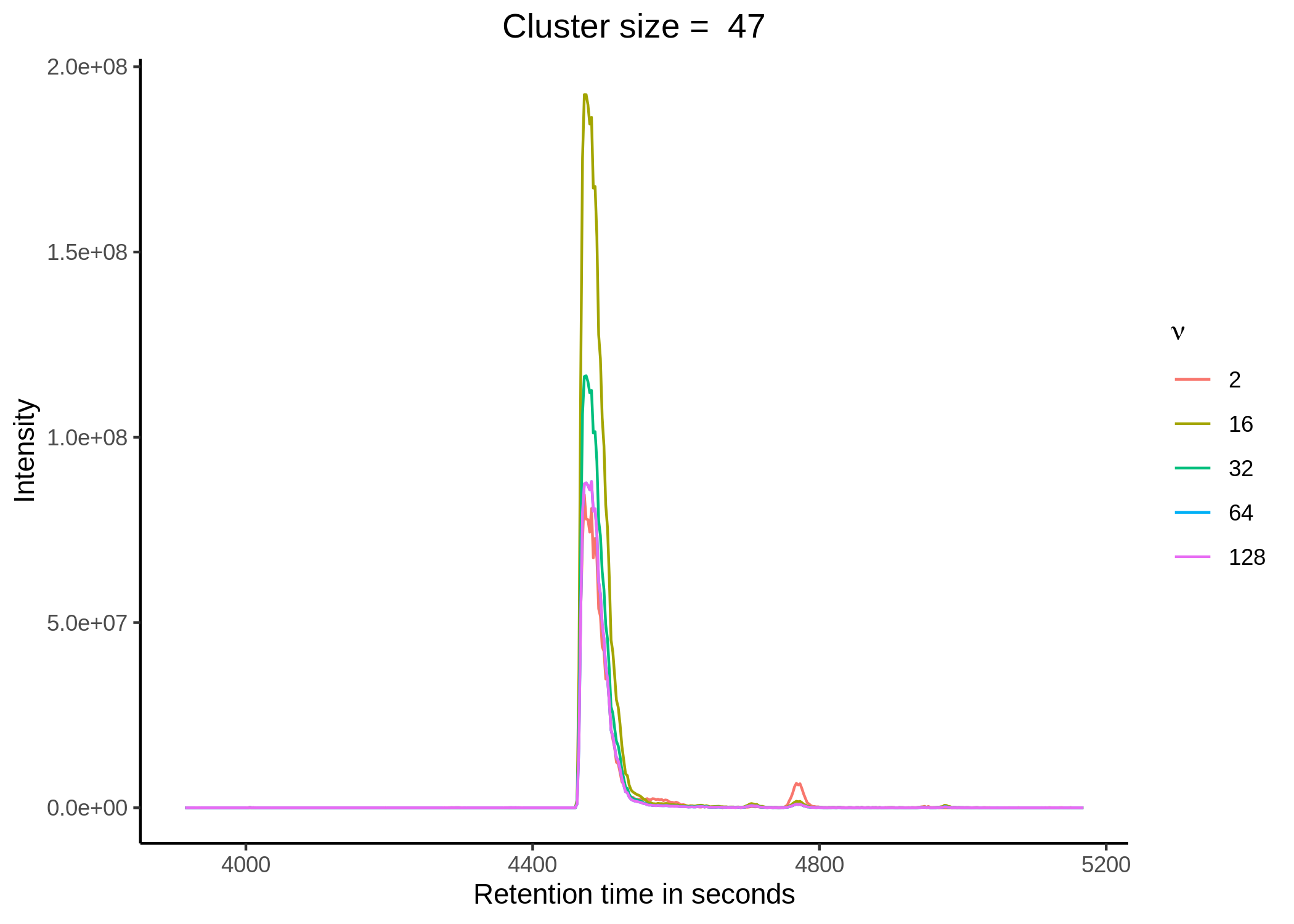

Supplement: Supplementary file 6 — Additional file 6: Consensus chromatogram stability. A set of 10 figures exemplifying the stability of the pre-image computation through the averaging of a neighborhood of varying size. [file 12859_2021_3969_MOESM6_ESM.zip › Consensus_chromatogram 1029 .png]

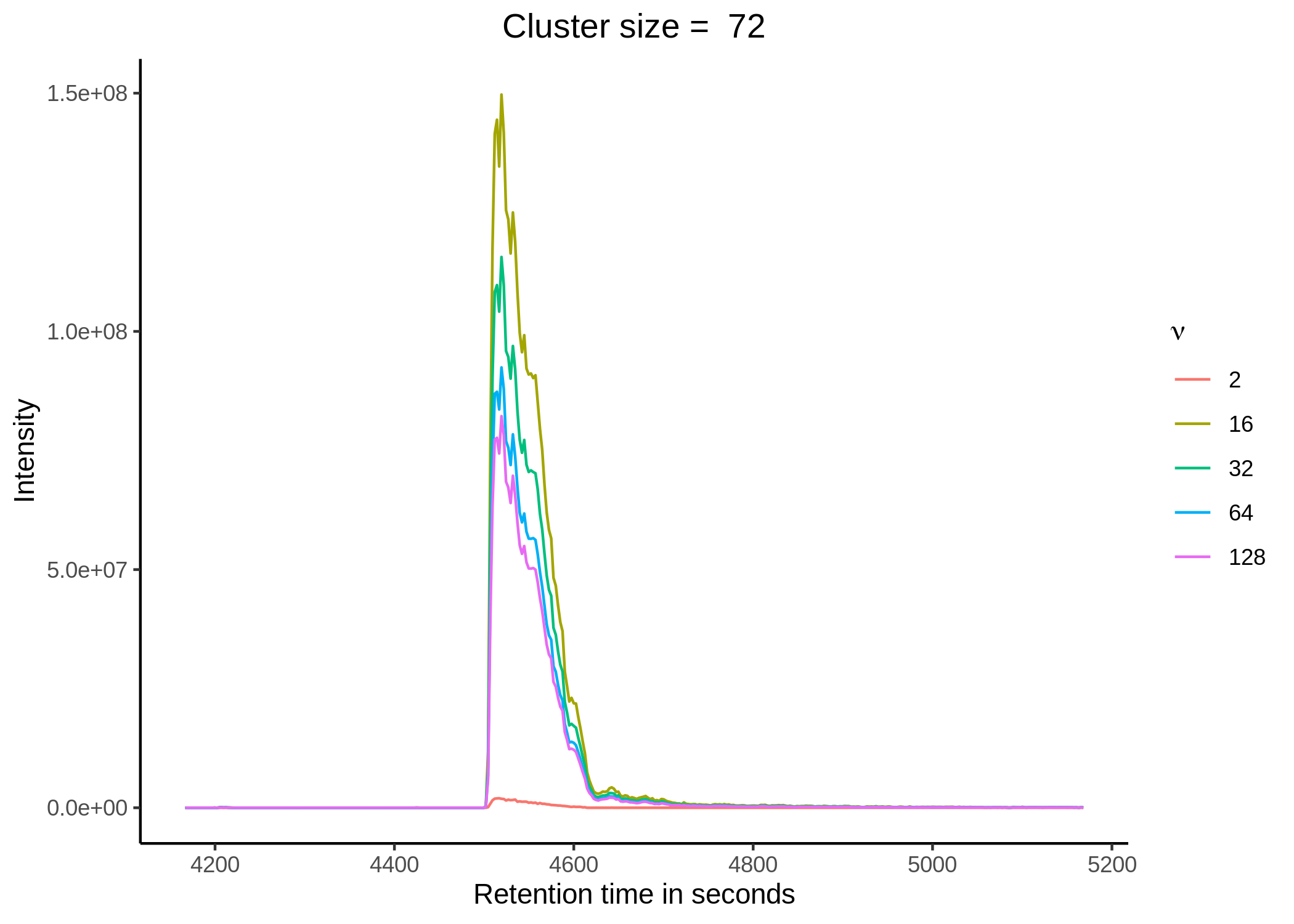

Supplement: Supplementary file 6 — Additional file 6: Consensus chromatogram stability. A set of 10 figures exemplifying the stability of the pre-image computation through the averaging of a neighborhood of varying size. [file 12859_2021_3969_MOESM6_ESM.zip › Consensus_chromatogram 1033 .png]

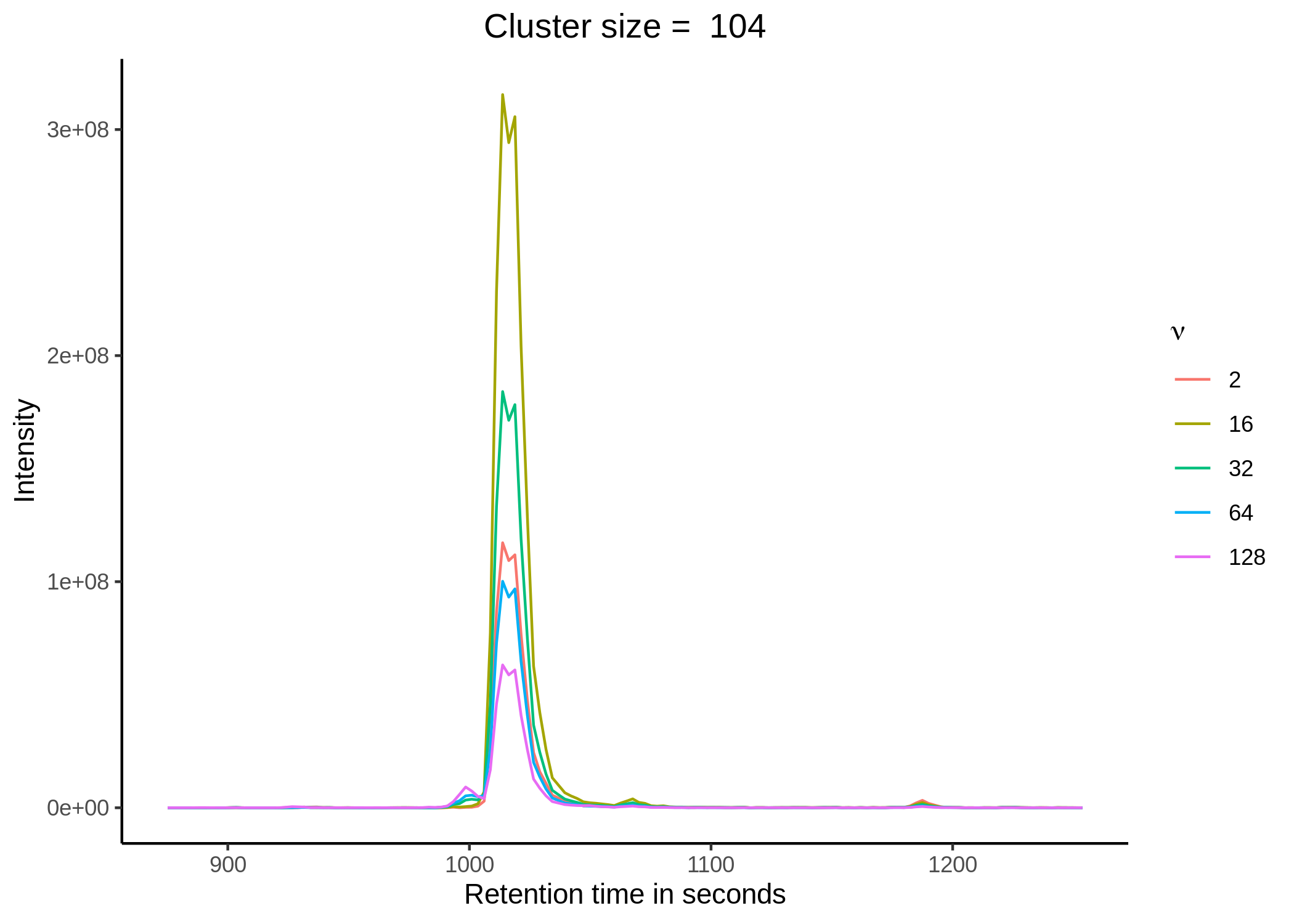

Supplement: Supplementary file 6 — Additional file 6: Consensus chromatogram stability. A set of 10 figures exemplifying the stability of the pre-image computation through the averaging of a neighborhood of varying size. [file 12859_2021_3969_MOESM6_ESM.zip › Consensus_chromatogram 1388 .png]

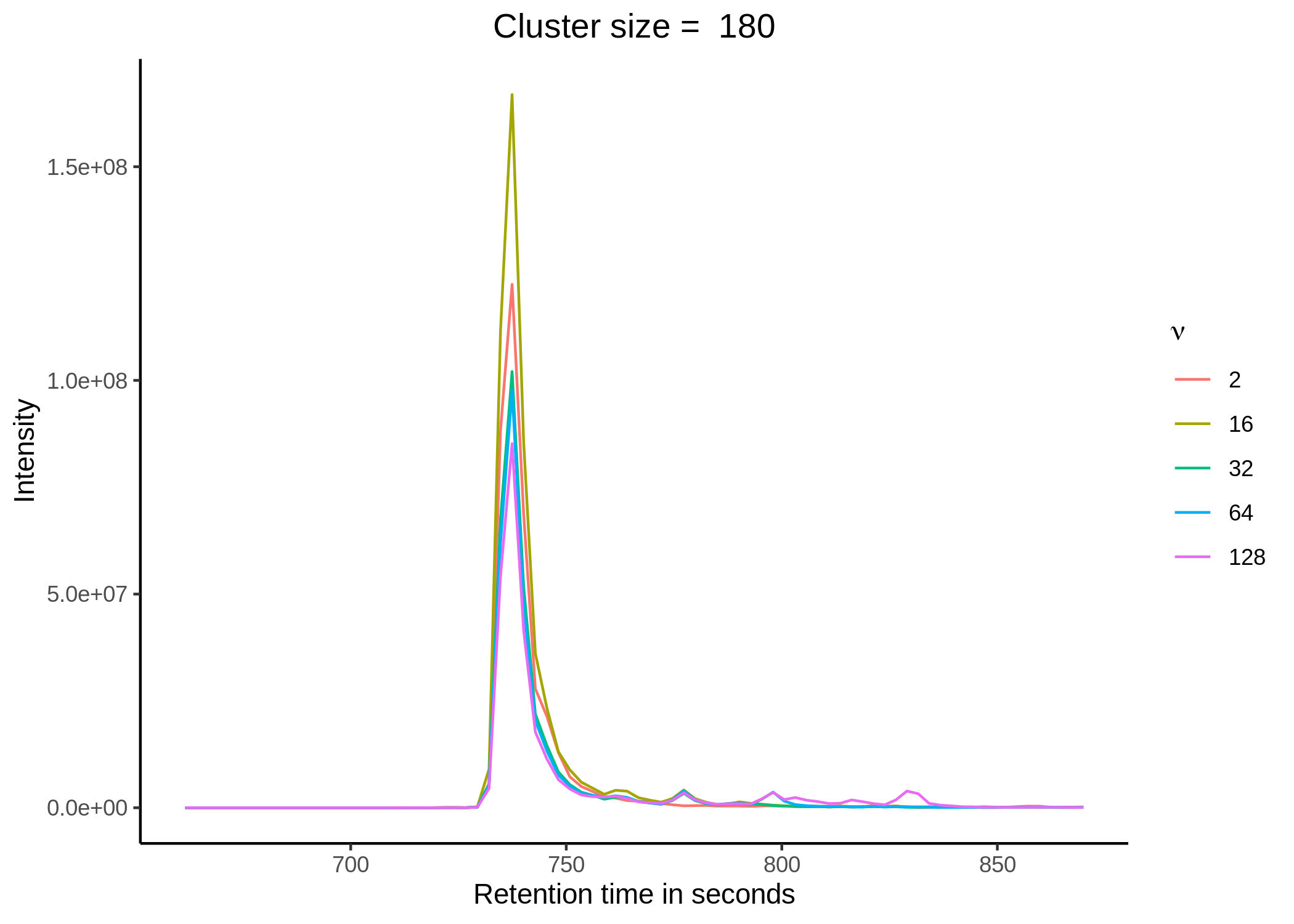

Supplement: Supplementary file 6 — Additional file 6: Consensus chromatogram stability. A set of 10 figures exemplifying the stability of the pre-image computation through the averaging of a neighborhood of varying size. [file 12859_2021_3969_MOESM6_ESM.zip › Consensus_chromatogram 1443 .png]

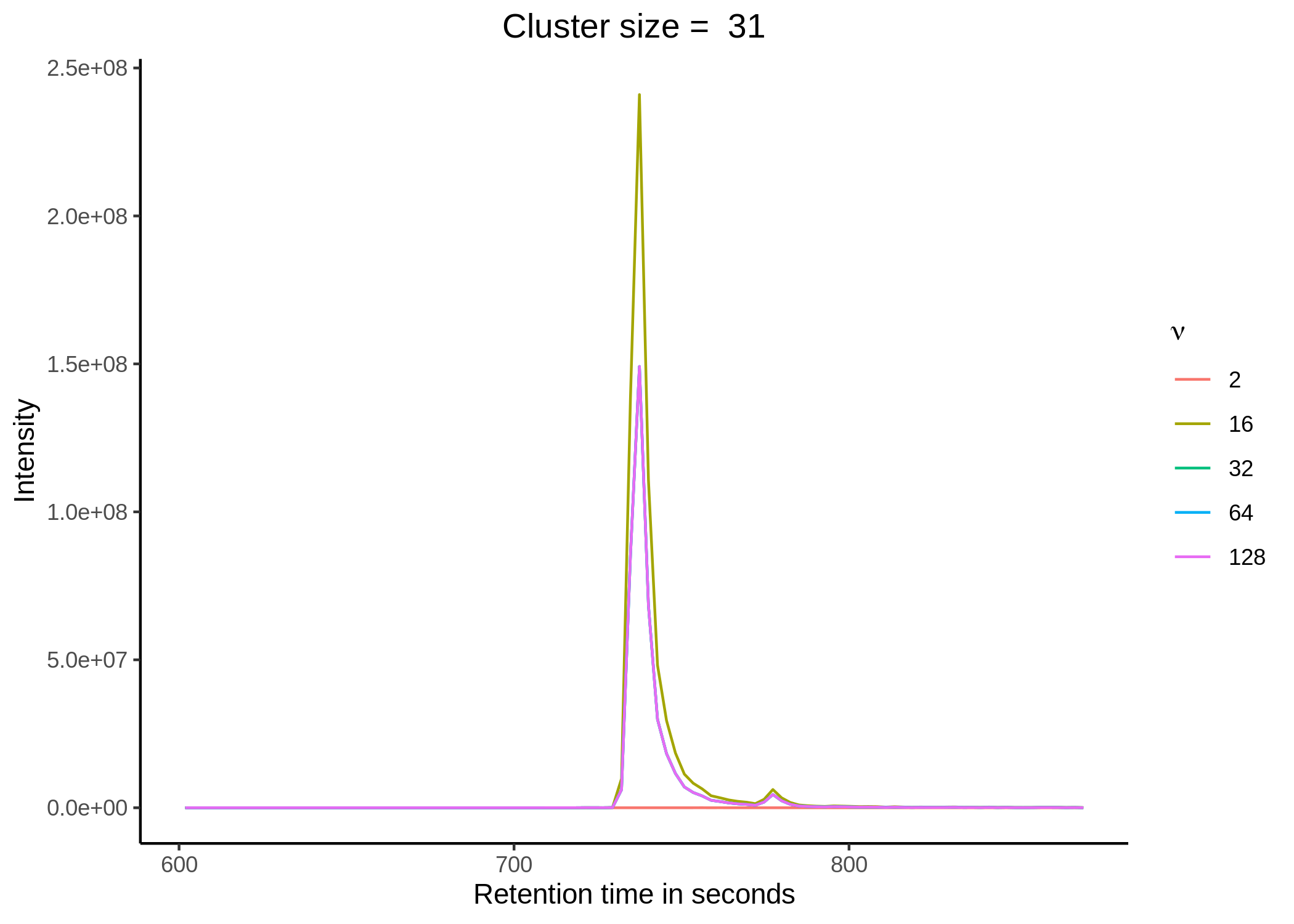

Supplement: Supplementary file 6 — Additional file 6: Consensus chromatogram stability. A set of 10 figures exemplifying the stability of the pre-image computation through the averaging of a neighborhood of varying size. [file 12859_2021_3969_MOESM6_ESM.zip › Consensus_chromatogram 4708 .png]

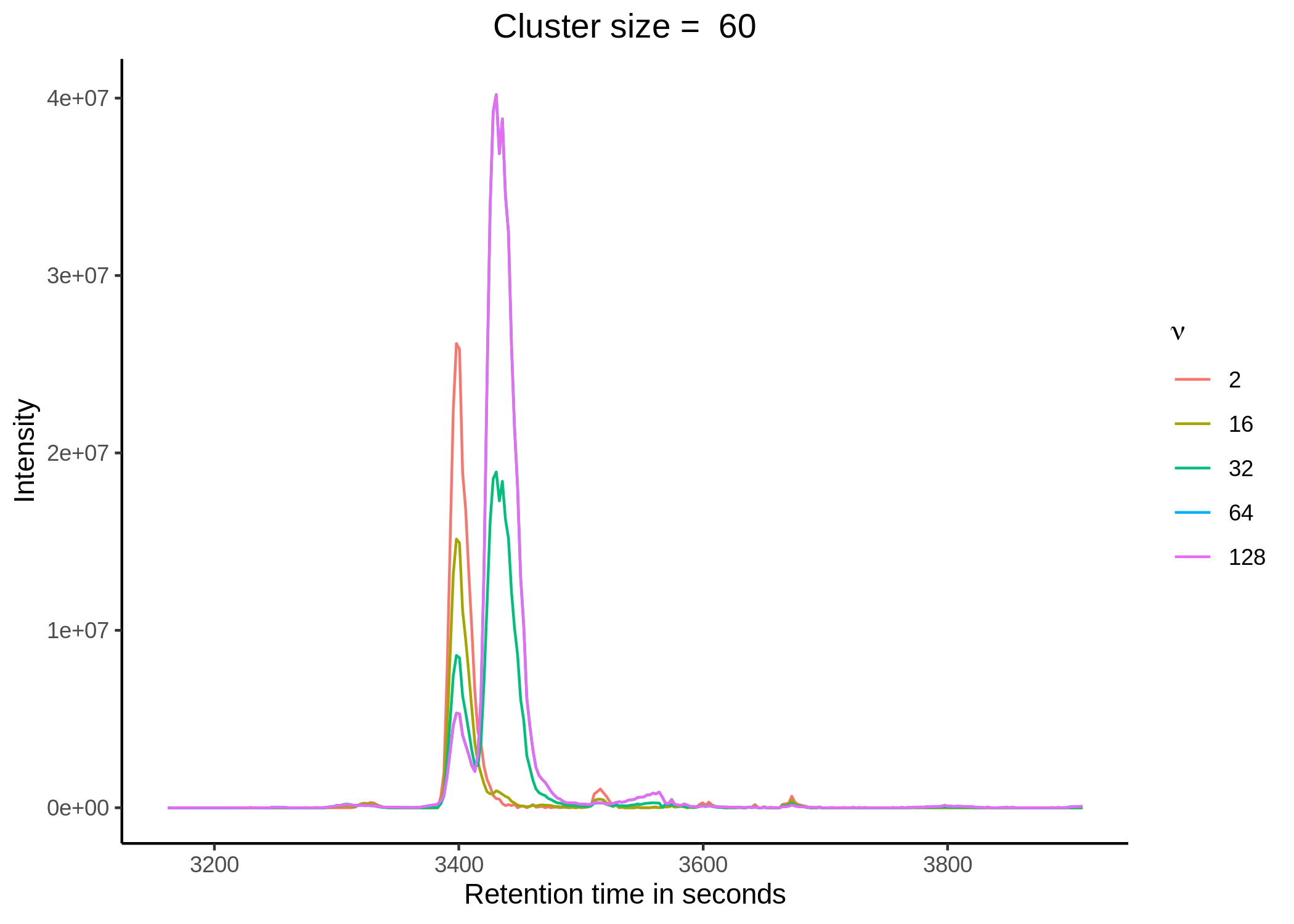

Supplement: Supplementary file 6 — Additional file 6: Consensus chromatogram stability. A set of 10 figures exemplifying the stability of the pre-image computation through the averaging of a neighborhood of varying size. [file 12859_2021_3969_MOESM6_ESM.zip › Consensus_chromatogram 5220 .png]

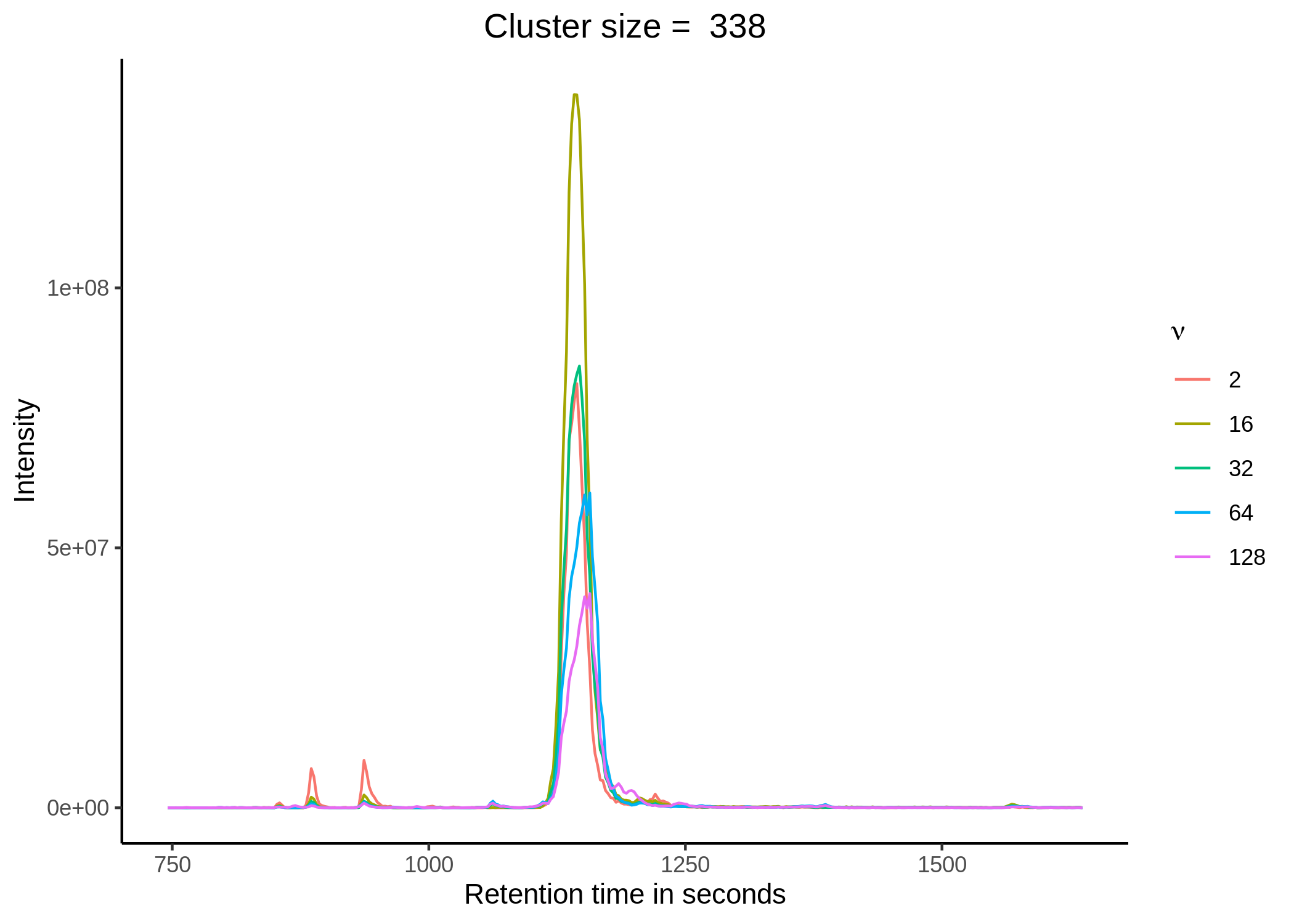

Supplement: Supplementary file 6 — Additional file 6: Consensus chromatogram stability. A set of 10 figures exemplifying the stability of the pre-image computation through the averaging of a neighborhood of varying size. [file 12859_2021_3969_MOESM6_ESM.zip › Consensus_chromatogram 6911 .png]

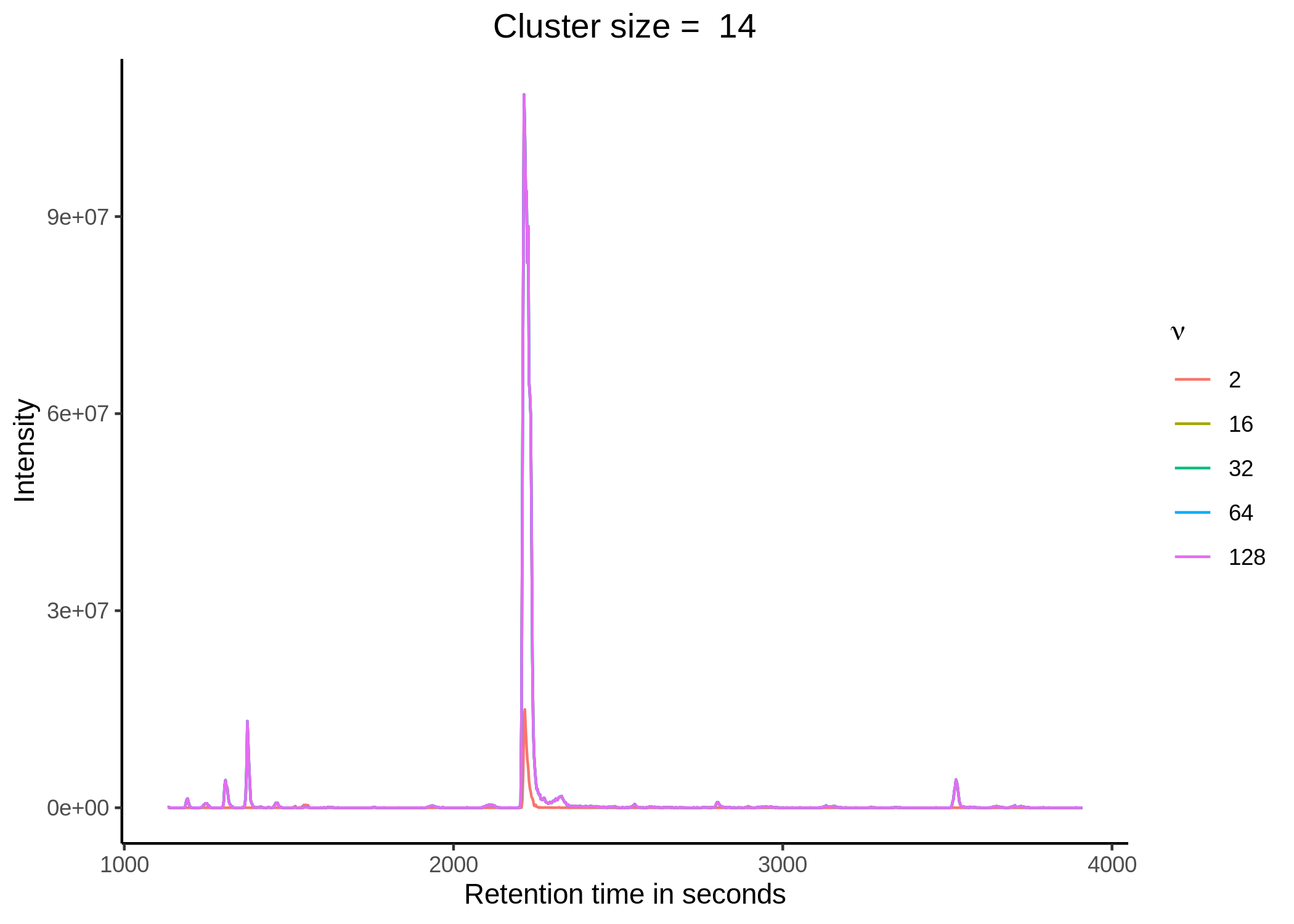

Supplement: Supplementary file 6 — Additional file 6: Consensus chromatogram stability. A set of 10 figures exemplifying the stability of the pre-image computation through the averaging of a neighborhood of varying size. [file 12859_2021_3969_MOESM6_ESM.zip › Consensus_chromatogram 7347 .png]

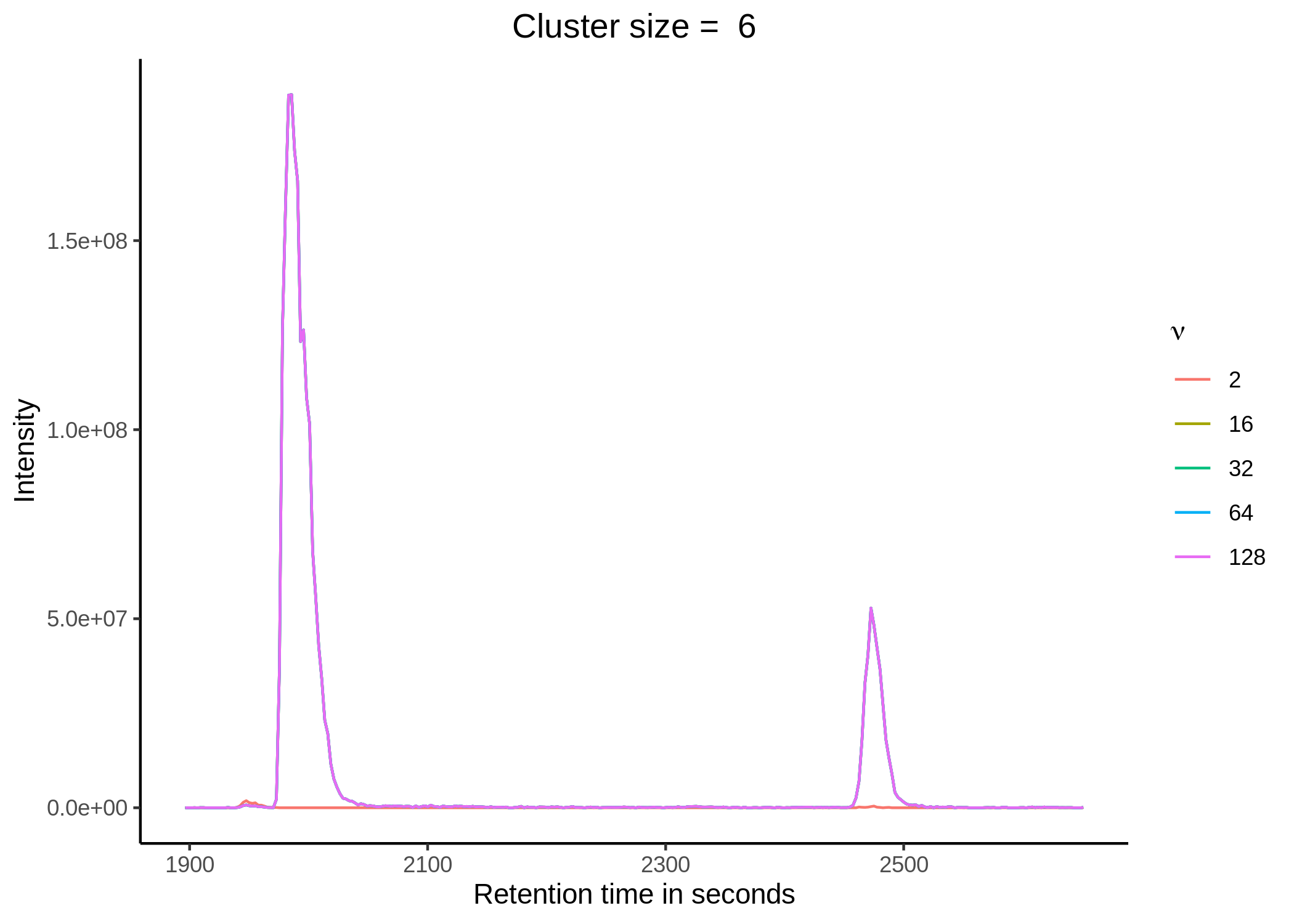

Supplement: Supplementary file 6 — Additional file 6: Consensus chromatogram stability. A set of 10 figures exemplifying the stability of the pre-image computation through the averaging of a neighborhood of varying size. [file 12859_2021_3969_MOESM6_ESM.zip › Consensus_chromatogram 7398 .png]

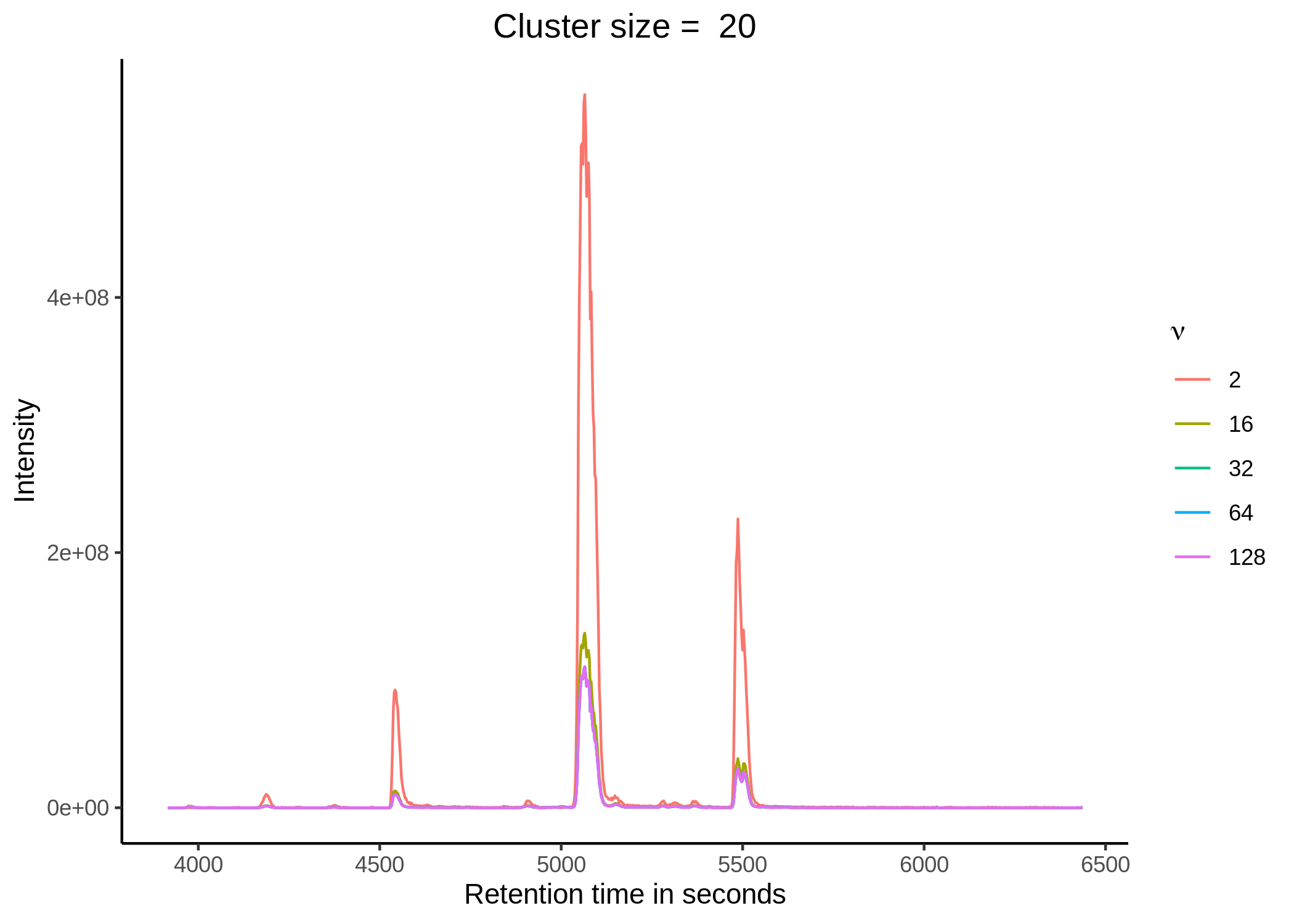

Supplement: Supplementary file 6 — Additional file 6: Consensus chromatogram stability. A set of 10 figures exemplifying the stability of the pre-image computation through the averaging of a neighborhood of varying size. [file 12859_2021_3969_MOESM6_ESM.zip › Consensus_chromatogram 8346 .png]

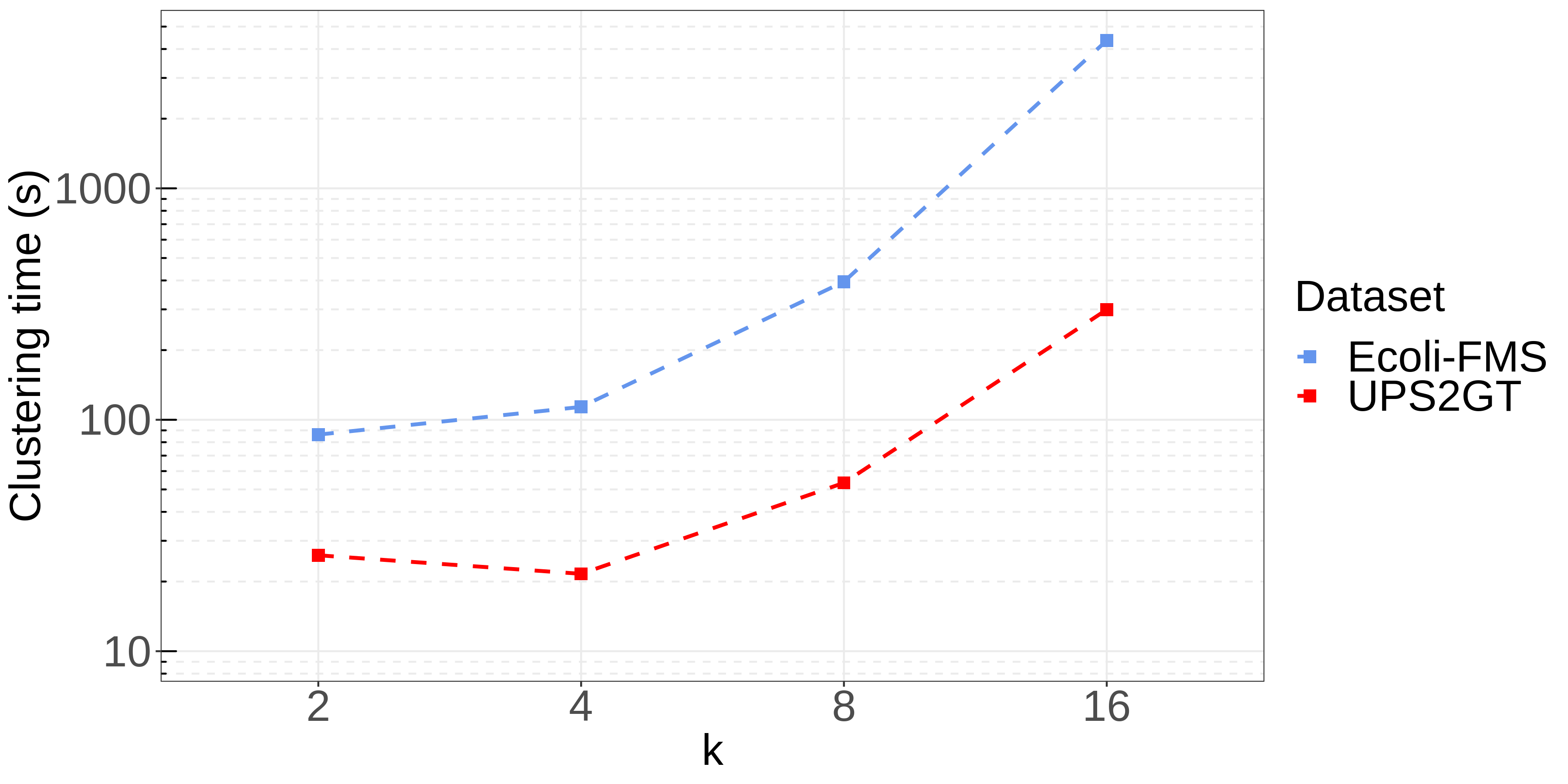

Supplement: Supplementary file 7 — Additional file 7: Influence of k on the execution time of CHICKN. Figure depicting CHICKN execution time as a function of k, the number of clusters at each iteration, for both UPS2GT (blue) and Ecoli-FMS (red) datasets. [file 12859_2021_3969_MOESM7_ESM.png]

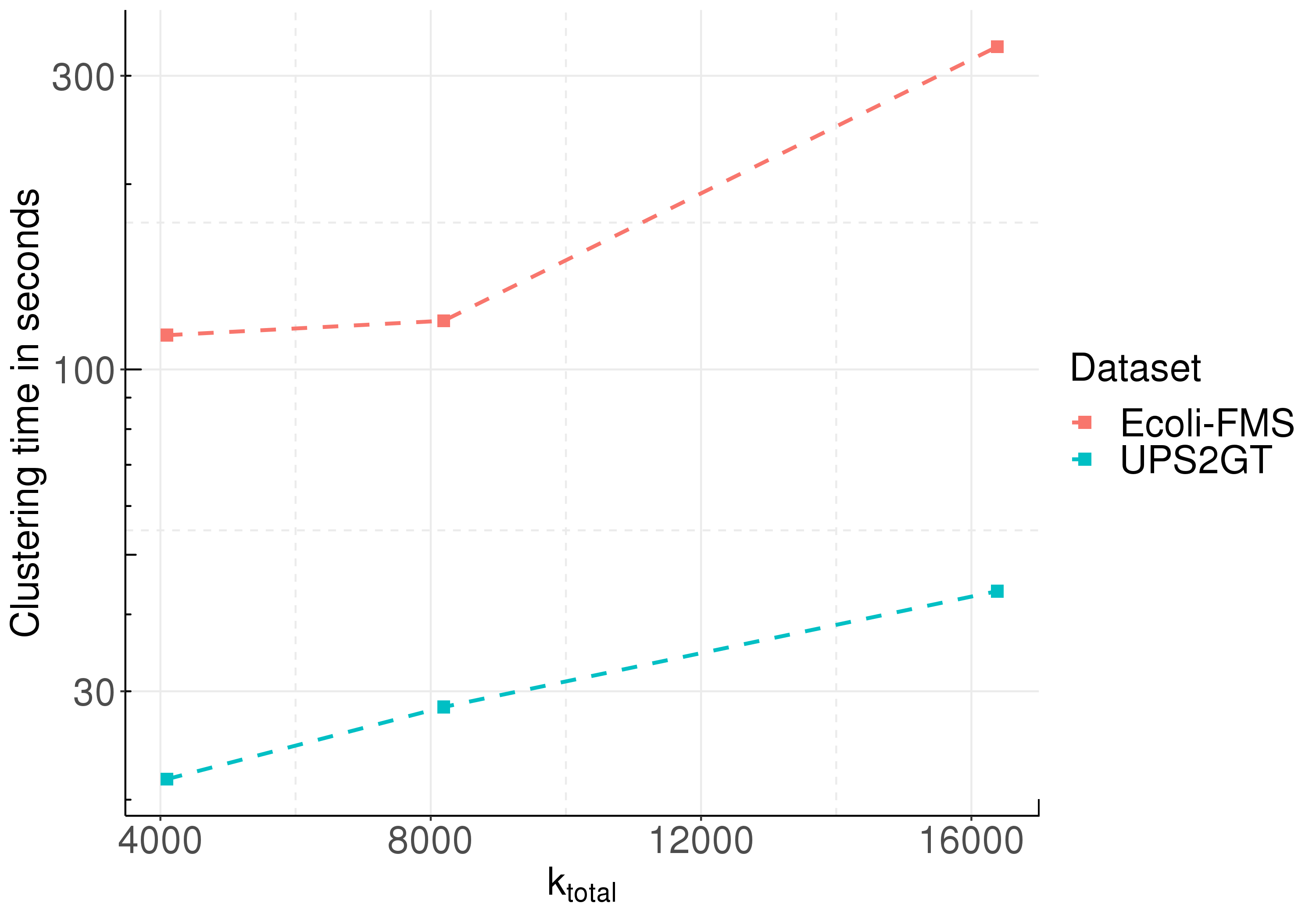

Supplement: Supplementary file 8 — Additional file 8: Influence of ktotal on the execution time of CHICKN. Figure depicting CHICKN execution time as a function of ktotal, the maximum number of clusters, for both UPS2GT (blue) and Ecoli-FMS (red) datasets. [file 12859_2021_3969_MOESM8_ESM.png]

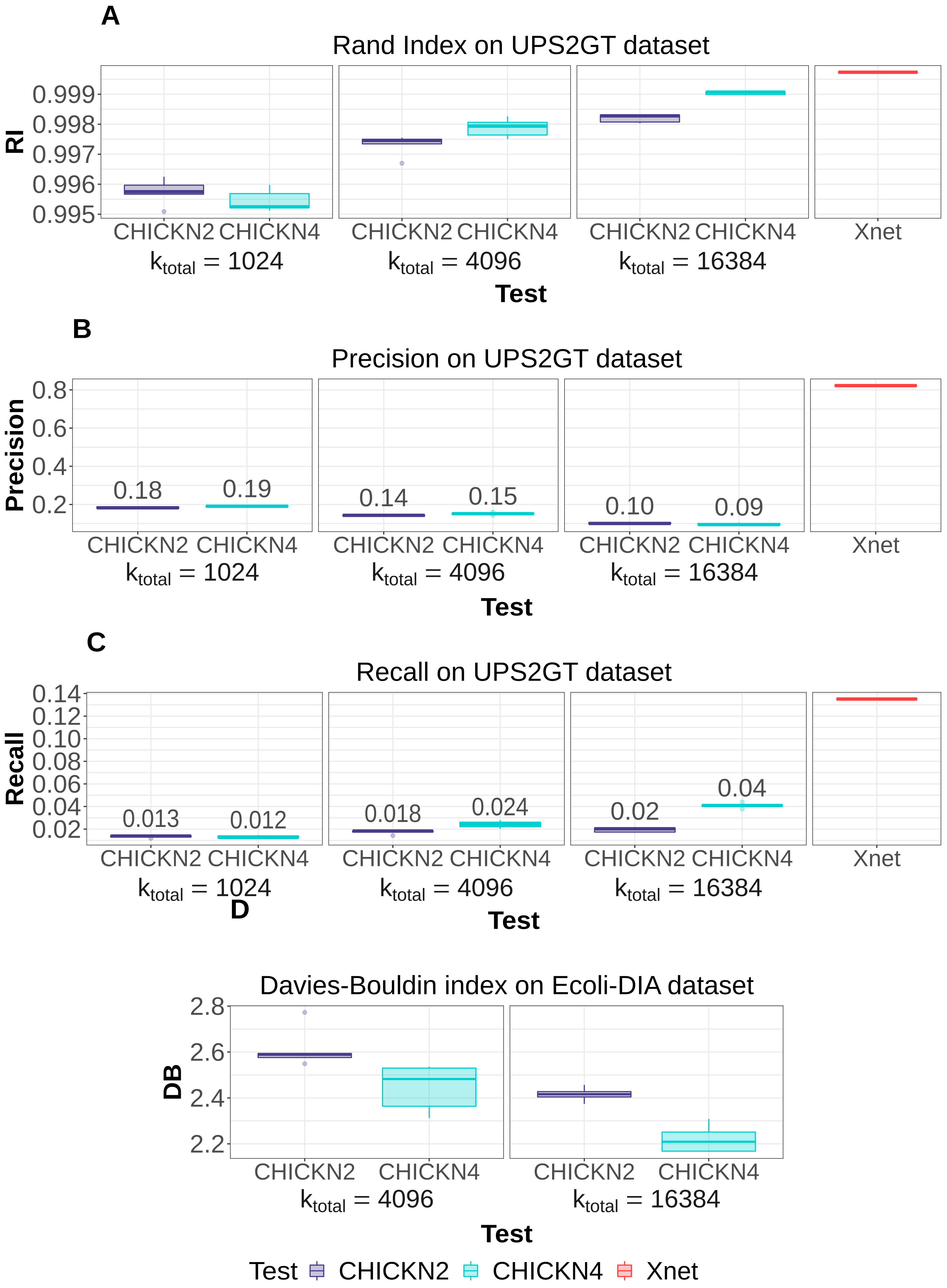

Supplement: Supplementary file 9 — Additional file 9: Performance evaluation for the Laplacian W1 kernel. This figure is the same as Fig. 4, yet with p = 1 instead of p = 2. The performances on the UPS2GT dataset are a bit lower than with the Gaussian W1 kernel (equivalent Rand index, better precision, lower recall), making it unable to compete with Xnet. However, on raw data such as Ecoli-DIA (i.e., on data CHICKN should work with), the Laplacian W1 kernel exhibit slightly better DB index than its Gaussian counterpart; however, this is hardly significant, making us conclude that strict performance should not be the criterion to choose the kernel. [file 12859_2021_3969_MOESM9_ESM.png]

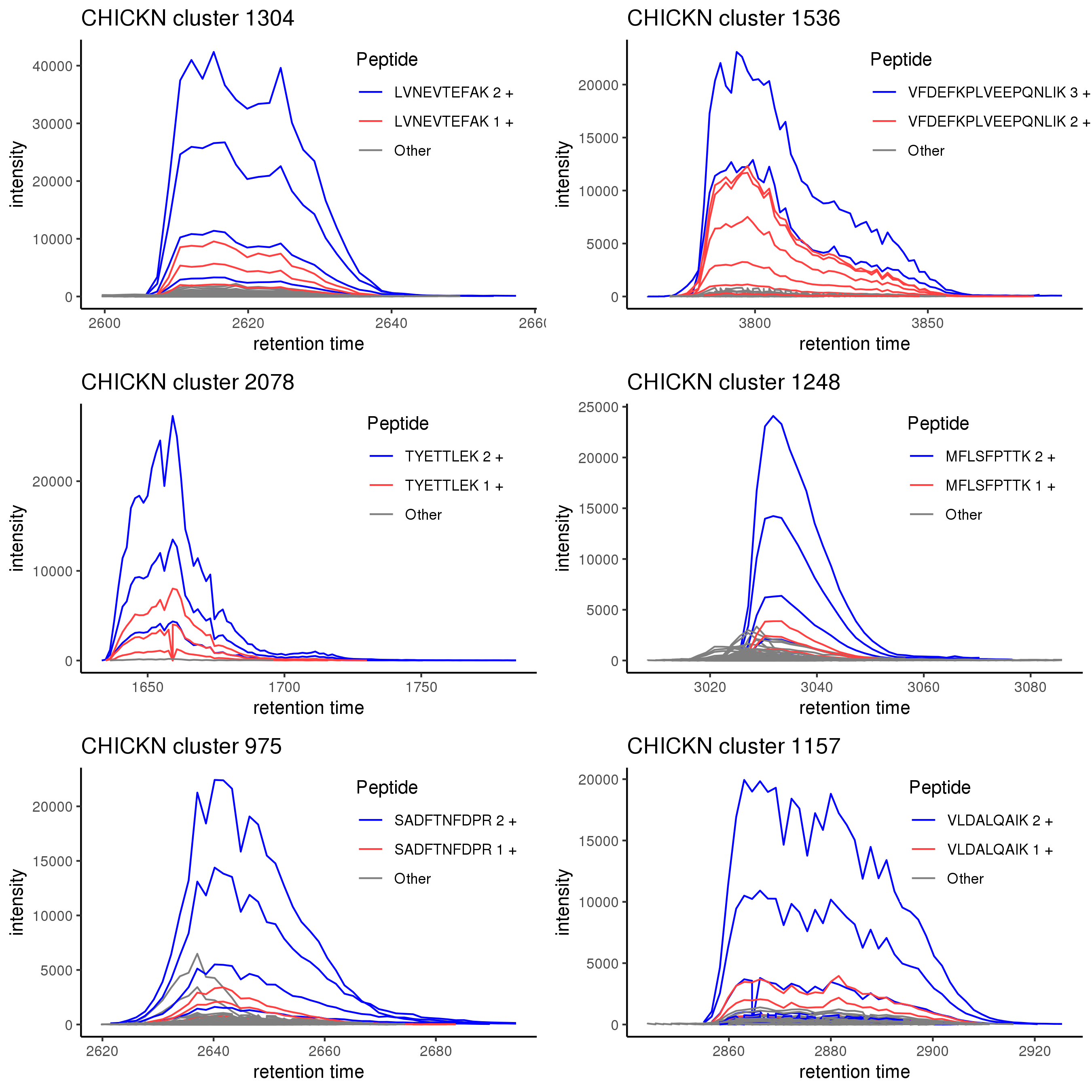

Supplement: Supplementary file 10 — Additional file 10: Differently charged ions of a same peptide tend to cluster together. A subset of clusters was manually inspected so as to label as many profiles with the corresponding identified ion. Although this labelling cannot be exhaustively conducted due to the largely incomplete coverage of MS/MS analysis, it could be established that ions of a same peptide cluster together in many cases. [file 12859_2021_3969_MOESM10_ESM.png]

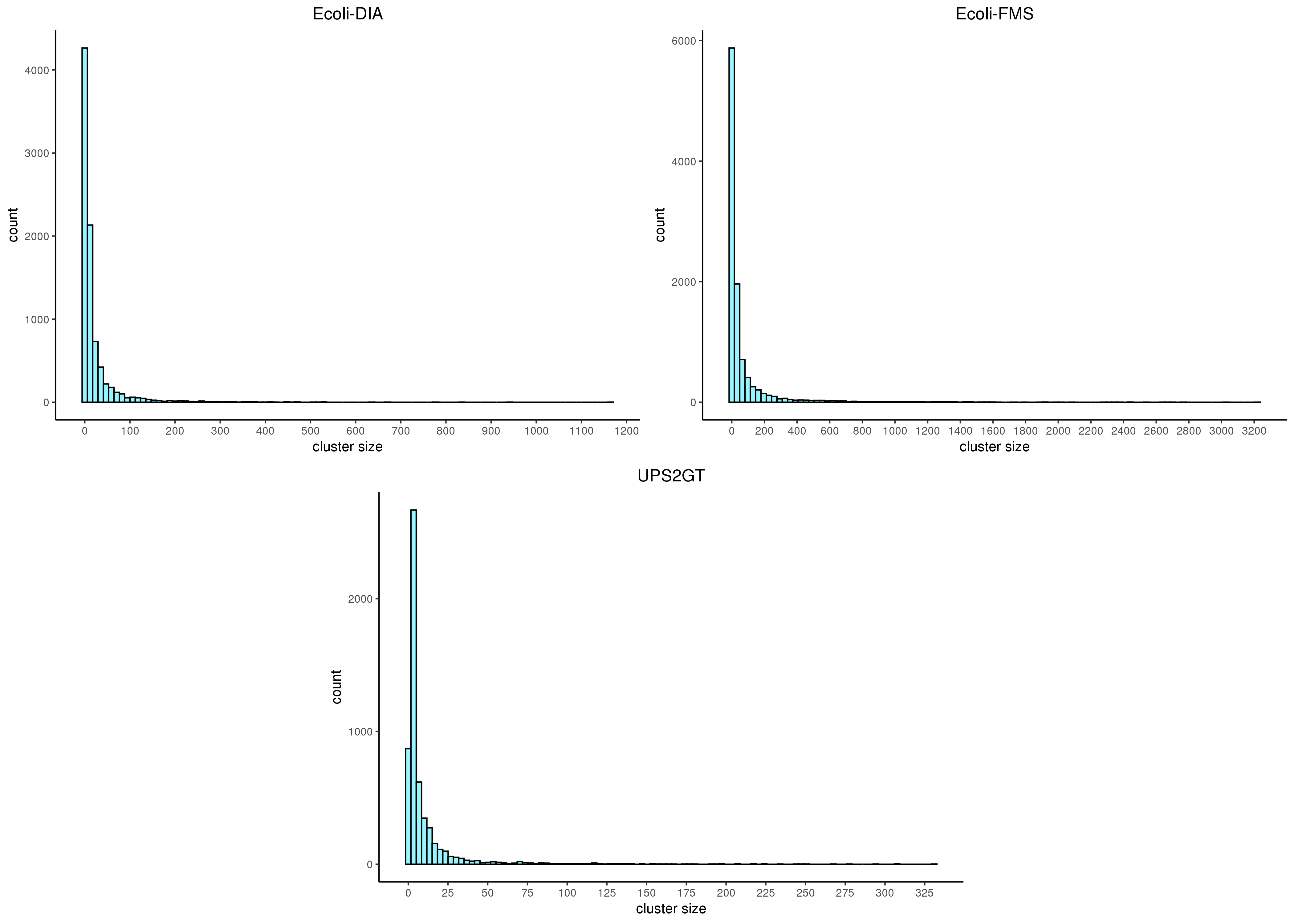

Supplement: Supplementary file 11 — Additional file 11: Cluster size distribution. Histograms of the cluster size distribution resulting from the application of CHICKN on each of the three datasets. [file 12859_2021_3969_MOESM11_ESM.png]

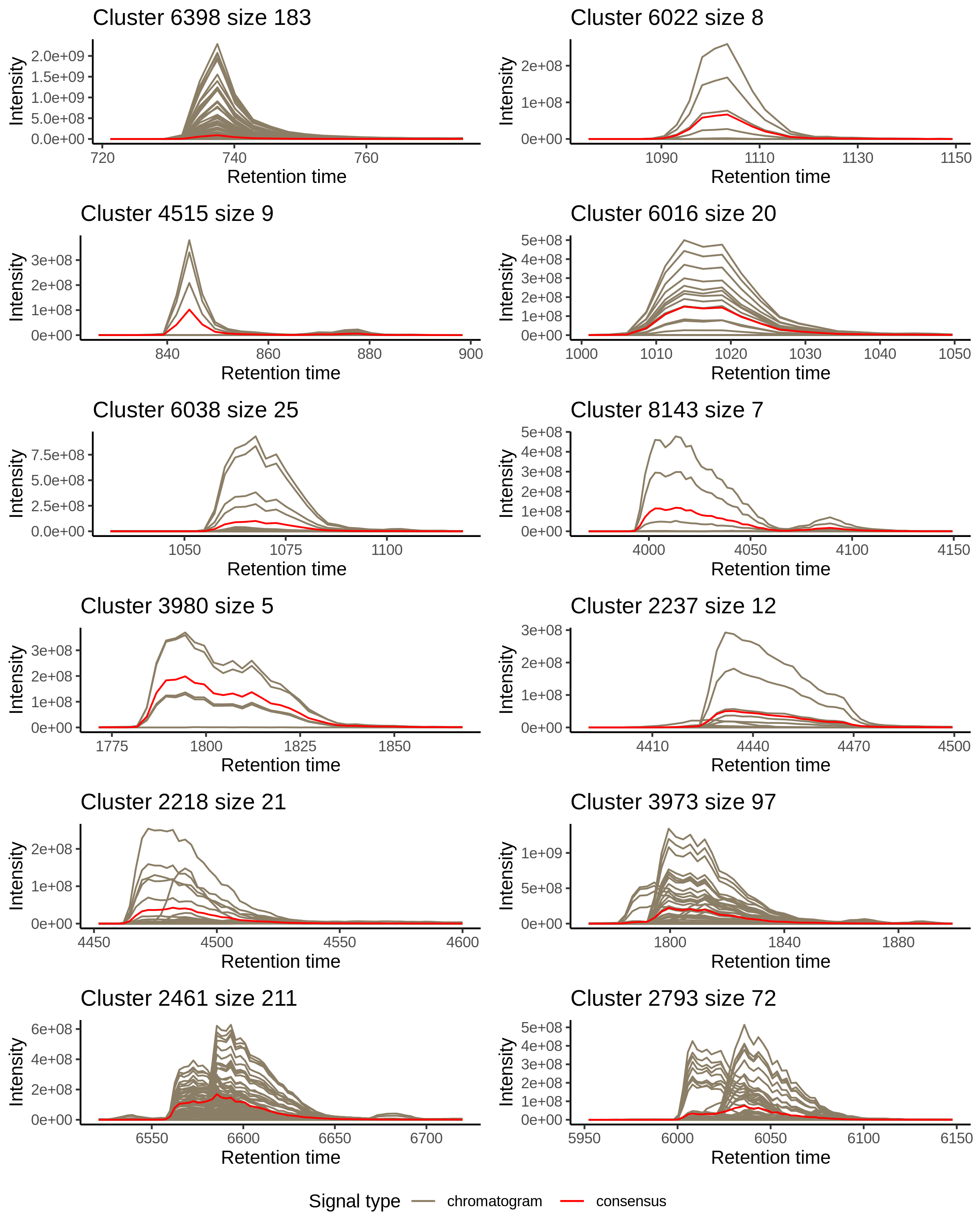

Supplement: Supplementary file 12 — Additional file 12: Examples of well-formed clusters for the Ecoli-FMS dataset. Same figure as Fig. 6 with Laplacian W1 kernel. [file 12859_2021_3969_MOESM12_ESM.png]

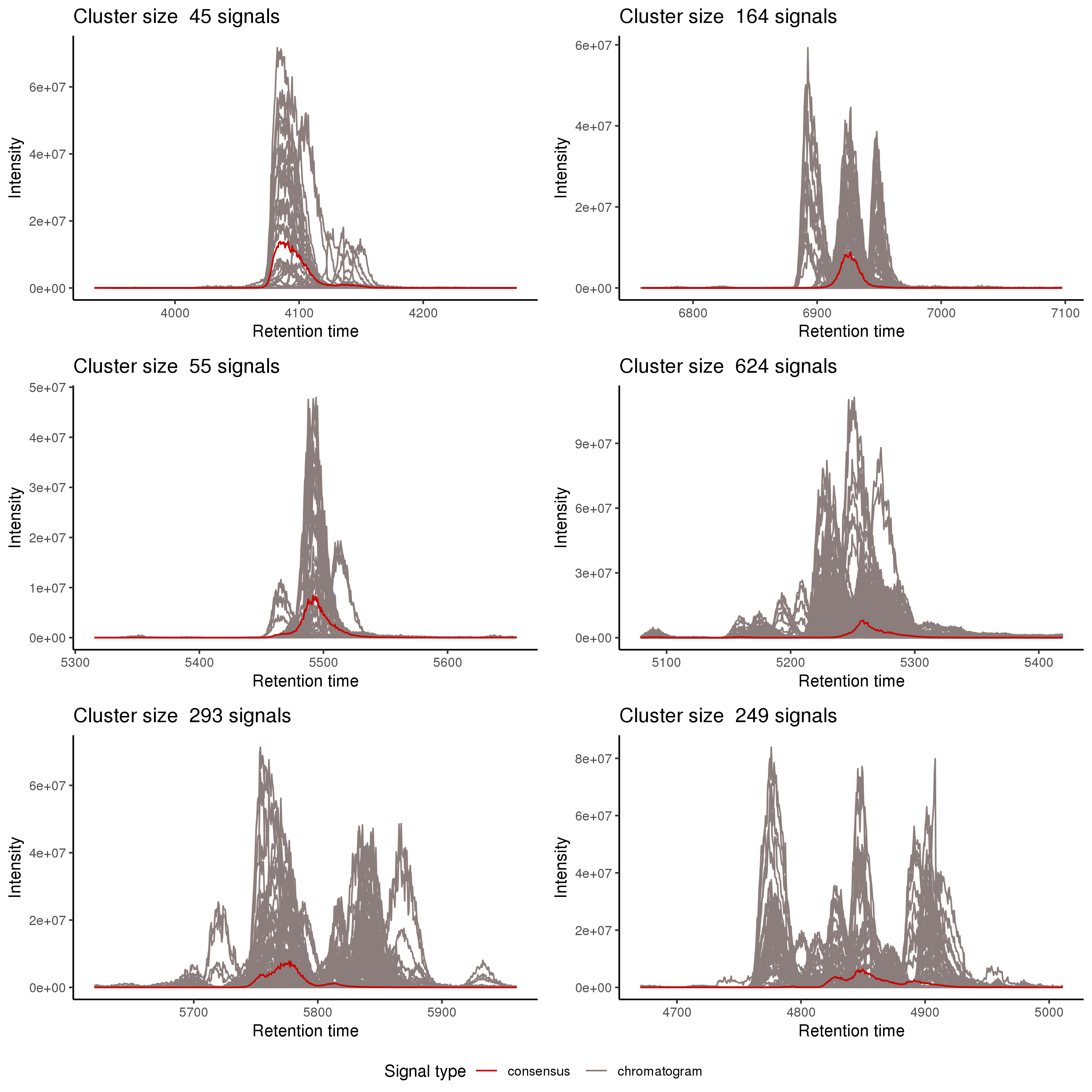

Supplement: Supplementary file 13 — Additional file 13: Examples of multiplexed clusters for the Ecoli-FMS dataset using CHICKN method. Figure illustrating that dividing multiplexed clusters into several sub-clusters would improve the elution profile interpretation. The real chromatograms and the consensus chromatograms are depicted in gray and in red, respectively. [file 12859_2021_3969_MOESM13_ESM.png]

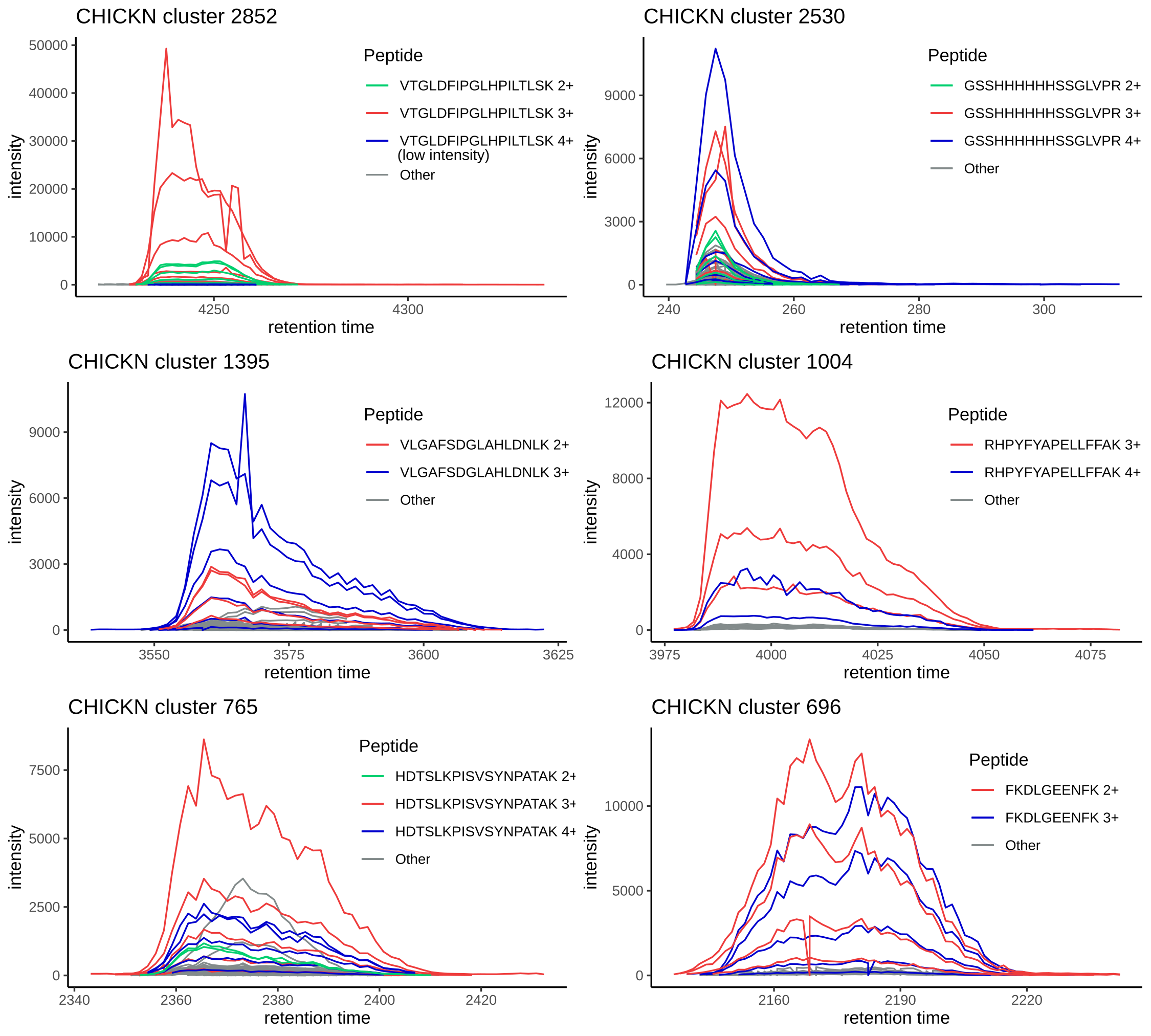

Supplement: Supplementary file 14 — Additional file 14: Differently charged ions of a same peptide tend to cluster together. Figure similar to Additional File 10. It depicts another subset of CHICKN clusters with chromatographic profiles manually annotated with the corresponding peptide ion. It could be established that ions of a same peptide tend to cluster together. [file 12859_2021_3969_MOESM14_ESM.png]
