# Supplementary material for: CHICKN: extraction of peptide chromatographic elution profiles from large scale mass spectrometry data by means of Wasserstein compressive hierarchical cluster analysis
Source: BMC Bioinformatics. 2021 Feb 12;22:68. doi: 10.1186/s12859-021-03969-0 (PMC7881590; doi:10.1186/s12859-021-03969-0)
Supplement: Supplementary file 2 — Additional file 2: Preprocessing details. Detailed explanations of Eq. (1) (interpolation needs, justification of the method and parameter tuning). [file 12859_2021_3969_MOESM2_ESM.pdf]

Non-uniform grid construction from RAW mass spectrometry data

Olga Permiakova, Romain Guibert, Alexandra Kraut,
Thomas Fortin, Anne-Marie Hesse, Thomas Burger

November 24, 2020

Additional file to "*Extraction of peptide chromatographic elution profiles from large scale mass spectrometry data by means of Wasserstein compressive hierarchical cluster analysis*" by Olga Permiakova, Romain Guibert, Alexandra Kraut, Thomas Fortin, Anne-Marie Hesse, Thomas Burger.

1 Motivation for grid-interpolation of LC-MS data

LC-MS data are acquired progressively, one mass spectrum after the other, which from a matrix viewpoint, means row-wise (see Figure 1 in the article). However, CHICKN requires to process elution profiles, *i.e.* accessing the data matrix column-wise. Unfortunately, direct access to elution profiles is not possible: The spectra are acquired and discretized independently from one another, with a non-uniform sampling of the m/z scale, which depends on (i) the m/z values (finer for smaller m/z values, coarser for larger ones); and (ii) the density of peaks (to avoid storing too many zeros between peaks). To cope for these, it is thus necessary to align the raw data, and to interpolate all spectra on a common grid. Moreover, to optimaly store and exploit the LC-MS data, the grid must respect the fluctuation of the m/z precision in function of the m/z value (as aforementioned, finer for smaller m/z values, coarser for larger ones).

2 Non-uniform grid construction

To unveil how the grid step should adapt across the m/z range, we computed the differences between consecutive m/z values (referred hereafter to as $\Delta m/z$) within each raw spectrum and analyzed their distribution in function of m/z over all spectra. However, some $\Delta m/z$ reach very high values, as over large m/z intervals devoid of peaks, the signal does not need to be sampled/stored. To cope for this, we have gotten rid of the $\Delta m/z$ for which at least one of the two intensity values was lower than $5 \cdot 10^4$. Although this strategy led to some information loss, the remaining one was largely sufficient to compute statistics and to derive conclusions on how the signal is discretized in the MS constructor data format. In fact, despite this reduction, the amount of obtained $([m/z]_i, [\Delta m/z]_i)$ pairs was still too large to

	Ecoli-DIA					Ecoli-FMS				
**m/z in Th**	400	800	1,000	1,200	1,400	400	800	1,000	1,200	1,400
**Δm/z in mTh**	2.0	5.66	7.9	10.39	13.09	0.5	1.41	1.98	2.6	3.27

Table 1: Some of $\Delta m/z$ values computed at different m/z values (in mili-thomsons) using Eq. (2) for Ecoli-DIA ($Res_{EXP} = 60,000$) and Ecoli-FMS ($Res_{EXP} = 240,000$) datasets.

be visualized on a single plot. This is why Figure 1 (respectively, Figure 2) depicts only a random 10% (respectively 2%) of them for Ecoli-DIA dataset (respectively, Ecoli-FMS dataset).

It can be observed on both figures that most of the $([m/z]_i, [\Delta m/z]_i)$ aggregate along a curve, which appears continuous and black (while it is in fact a collection of partly transparent crosses) because of the point density (the others, in proportion, rather few of them, being outliers). We empirically found that a power function of degree $\frac{3}{2}$ (dashed red line) led to a perfect fit on the data.

In addition, we noticed that the magnitudes of $\Delta m/z$ in the Ecoli-DIA dataset were 4 times larger than those in the Ecoli-FMS one. As the ratio of instrument resolutions is also exactly 4, a simple regression provided us with the missing coefficient $\frac{0.015}{Res_{EXP}}$, with Res_{EXP} being the resolution tuning of the instrument. Finally, we obtain:

$$[\Delta m/z]_i = \frac{0.015}{Res_{EXP}}[m/z]_i^{\frac{3}{2}}, \quad \forall\, i = 0, \ldots, N-1, \tag{1}$$

where N is the last grid index of the grid. Using a more convenient notation where m_i stands for $[m/z]_i$, one obtains Equation (1) from the article:

$$m_{i+1} - m_i = \frac{0.015}{Res_{EXP}} m_i^{\frac{3}{2}} \tag{2}$$

Table 1 illustrates the change of the grid step ($\Delta m/z$, expressed in mili-thomsons) obtained using Eq. (2) depending on the m/z values for both considered datasets.

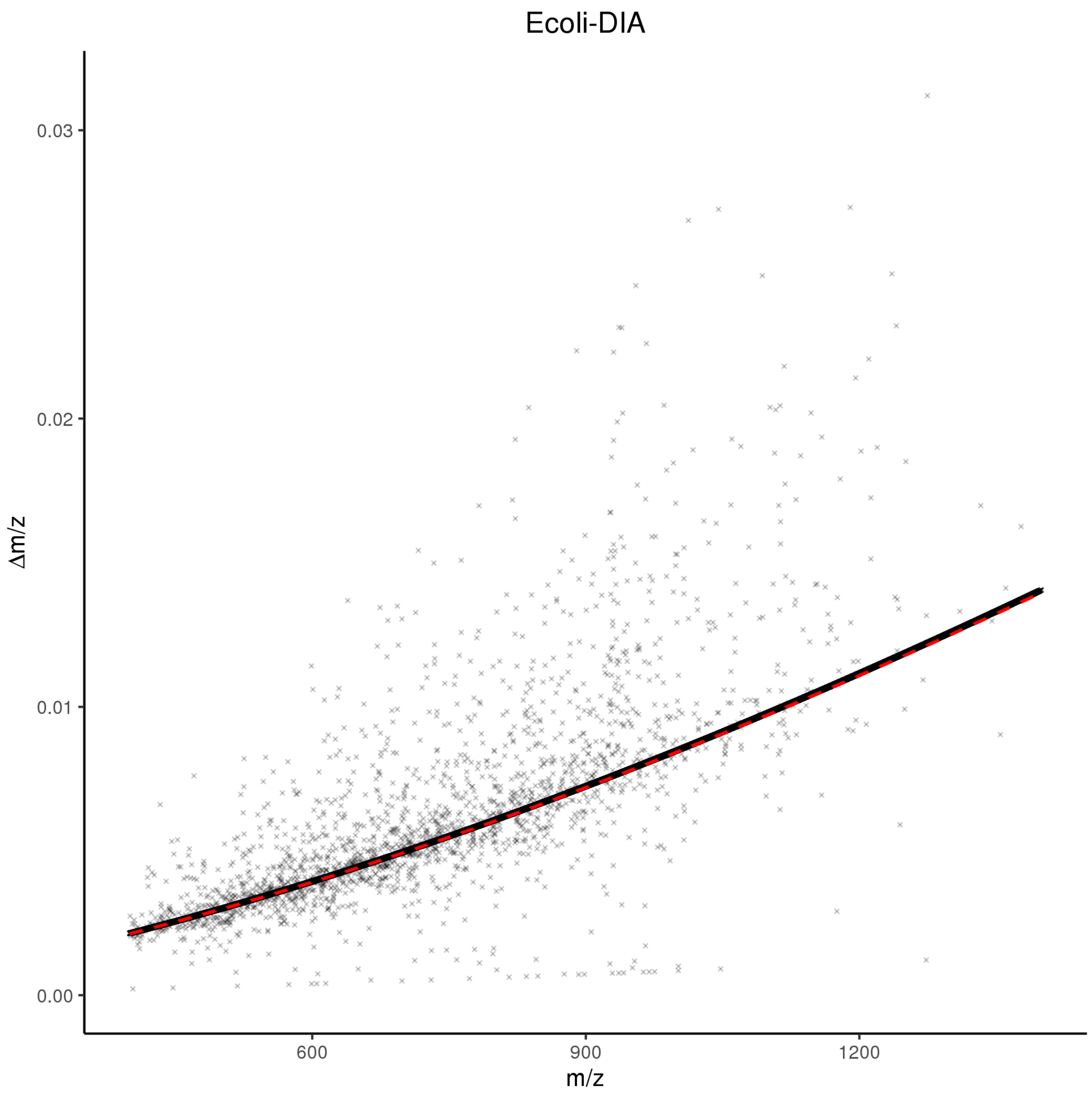

Figure 1: Gray crosses (in fact, black transparent crosses) depict (m/z, $\Delta m/z$) pairs computed from raw Ecoli-DIA dataset. Red dashed line depicts Equation (1).

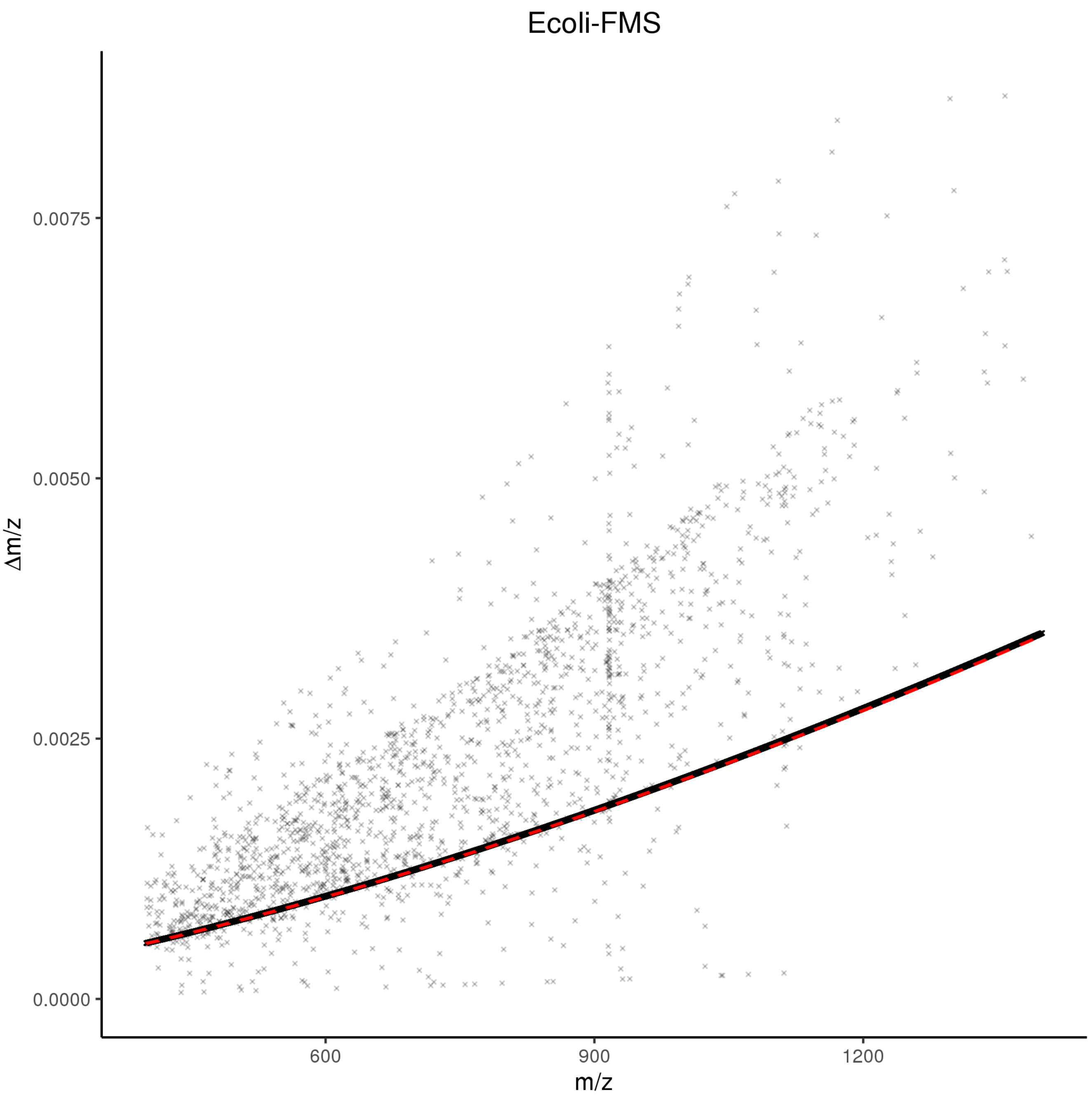

Figure 2: Gray crosses (in fact, black transparent crosses) depict (m/z, $\Delta m/z$) pairs computed from raw Ecoli-FMS dataset. Red dashed line depicts Equation (1).
