# Supplementary material for: CHICKN: extraction of peptide chromatographic elution profiles from large scale mass spectrometry data by means of Wasserstein compressive hierarchical cluster analysis
Source: BMC Bioinformatics. 2021 Feb 12;22:68. doi: 10.1186/s12859-021-03969-0 (PMC7881590; doi:10.1186/s12859-021-03969-0)
Supplement: Supplementary file 3 — Additional file 3: Kernel positive (semi-)definiteness. Empirical evidences (Gaussian W1 case) and formal demonstration (Laplacian W1 case) of the P(S)D-ness of the proposed kernels. [file 12859_2021_3969_MOESM3_ESM.pdf]

Positive (semi-)definiteness of Wasserstein-1 based kernels for real-valued signals

Olga Permiakova, Romain Guibert, Alexandra Kraut,
Thomas Fortin, Anne-Marie Hesse, Thomas Burger

September 17, 2020

Additional file to "*Extraction of peptide chromatographic elution profiles from large scale mass spectrometry data by means of Wasserstein compressive hierarchical cluster analysis*" by Olga Permiakova, Romain Guibert, Alexandra Kraut, Thomas Fortin, Anne-Marie Hesse, Thomas Burger.

1 Notations and definitions

Definition 1 (Real-valued series). *This work focuses on real-valued signals, discretized on t time stamps, such as for instance $x := [x^1, \dots, x^t]$ which can simply be referred to as vector $x \in \mathbb{R}^t$.*

Definition 2 (Wasserstein-1 distance on real-valued series). *Let $x, y \in \mathbb{R}^t$. The W1 distance between x and y reads:*

$$d_{W1}(x, y) = \sum_{k=1}^{t} |F_x(k) - F_y(k)| = \|F_x - F_y\|_{\ell_1}$$

where F_x and F_y are the empirical cumulative functions of signals x and y, respectively:

$$F_x(k) = \sum_{i=1}^{k(k \leq t)} \frac{x^i}{\|x\|_{\ell_1}}$$

and

$$F_y(k) = \sum_{i=1}^{k(k \leq t)} \frac{y^i}{\|y\|_{\ell_1}}$$

Definition 3 (Positive definite and positive semi-definite kernel). *A kernel $k(\cdot, \cdot)$ is positive semi-definite (PSD) (respectively, positive definite (PD)) if and only if it is symmetric and for any choice of n distinct $x_1, \dots, x_n \in \mathbb{R}^t$ (respectively, $\in \mathbb{R}^t \setminus 0$) and of $c_1, \dots, c_n \in \mathbb{R}$:*

$$\sum_{i,j=1}^{n} c_i c_j k(x_i, x_j) \geq 0 \quad (\textit{respectively}, \; > 0). \tag{1}$$

Property 1. *A kernel k is PSD if and only if for any set of n distinct $x_1, \ldots, x_n \in \mathbb{R}^t$, the **kernel matrix** $K \in \mathbb{R}^{n \times n}$ defined by $K_{ij} = k(x_i, x_j)$ has only non-negative eigenvalues.*

Proof. [1] (Theorem 4.1.10 p.231) □

Definition 4 (Gaussian W1 kernel). $\forall\ x, y \in \mathbb{R}^t, \forall\ \gamma \in \mathbb{R}_+^*$, *the Gaussian W1 kernel reads:*

$$k_{GW1}^{\gamma}(x, y) = e^{-\gamma \cdot d_{W_1}(x,y)^2} \tag{2}$$

Definition 5 (Laplacian W1 kernel). $\forall\ x, y \in \mathbb{R}^t, \forall\ \gamma \in \mathbb{R}_+^*$, *the Laplacian W1 kernel reads:*

$$k_{LW1}^{\gamma}(x, y) = e^{-\gamma \cdot d_{W_1}(x,y)} \tag{3}$$

Definition 6 (Exponential 1D kernel [2]). $\forall\ x, y \in \mathbb{R}, \forall \gamma \in \mathbb{R}_+^*$, *the Exponential 1D kernel reads:*

$$k_{E1D}^{\gamma}(x, y) = e^{-\gamma \cdot |x-y|} \tag{4}$$

Property 2. $\forall\ x, y \in \mathbb{R}$, $\gamma \in \mathbb{R}_+^*$, *the Exponential 1D kernel $k_{E1D}^{\gamma}(x, y)$ is positive definite.*

Proof. [3] (Corollary 2.10. p. 78 and Theorem 2.2 p. 74) □

2 Gaussian W1 kernel

Conjecture 1. $\forall x, y \in \mathbb{R}^t, \forall\ \gamma \in \mathbb{R}_+^*$ *the kernel $k_1^{\gamma}(x, y) = e^{-\gamma \cdot \|x-y\|_{\ell_1}^2}$ is positive definite.*

If **Conjecture 1** holds, then, demonstrating the positive definiteness of k_{GW1}^{γ} is possible by following a line akin to the one used in the k_{LW1}^{γ} case (see Section 3).

Nevertheless, we provide here empirical supports for the PSD-ness of k_{GW1}^{γ} (which is sufficient to apply the kernel trick): For each dataset, we performed 5 Nyström approximations of the Gaussian W1 kernel matrix, as described in Algorithm 1 (main article) with different random subsampling, and we verified that all the eigenvalues were non-negative (leading to a PSD kernel, according to **Property 1**). The results are reported on Figures 1, 2 and 3, which display the 5 series of eigenvalues (for datasets Ecoli-DIA, Ecoli-FMS and UPS2GT, respectively), sorted by decreasing order, together with the largest (λ_{max}) and smallest (λ_{min}) eigenvalues across all the 5 tests. In addition, we observed that for raw data like Ecoli ones, for which CHICKN was designed, the λ_{min} is clearly positive (contrarily to datasets such as UPS2GT, which by construction may not lead to full rank data matrices). This makes us optimistic about **Conjecture 1**.

3 Laplacian W1 kernel

Lemma 1. *Let $(X_i)_{i=1}^m$ is a sequence of non empty sets, $\forall i \in \{1, \ldots, m\}$ $x^i, y^i \in X_i$ and $(k_i)_{i=1}^m$ is a sequence of positive definite kernels such that $k_i : X_i \times X_i \to \mathbb{R}$, then a kernel defined as:*

$$K((x^1, \ldots, x^m), (y^1, \ldots, y^m)) = \prod_{i=1}^{m} k(x_i, y_i) \tag{5}$$

is positive definite on $X_1 \times \cdots \times X_m$.

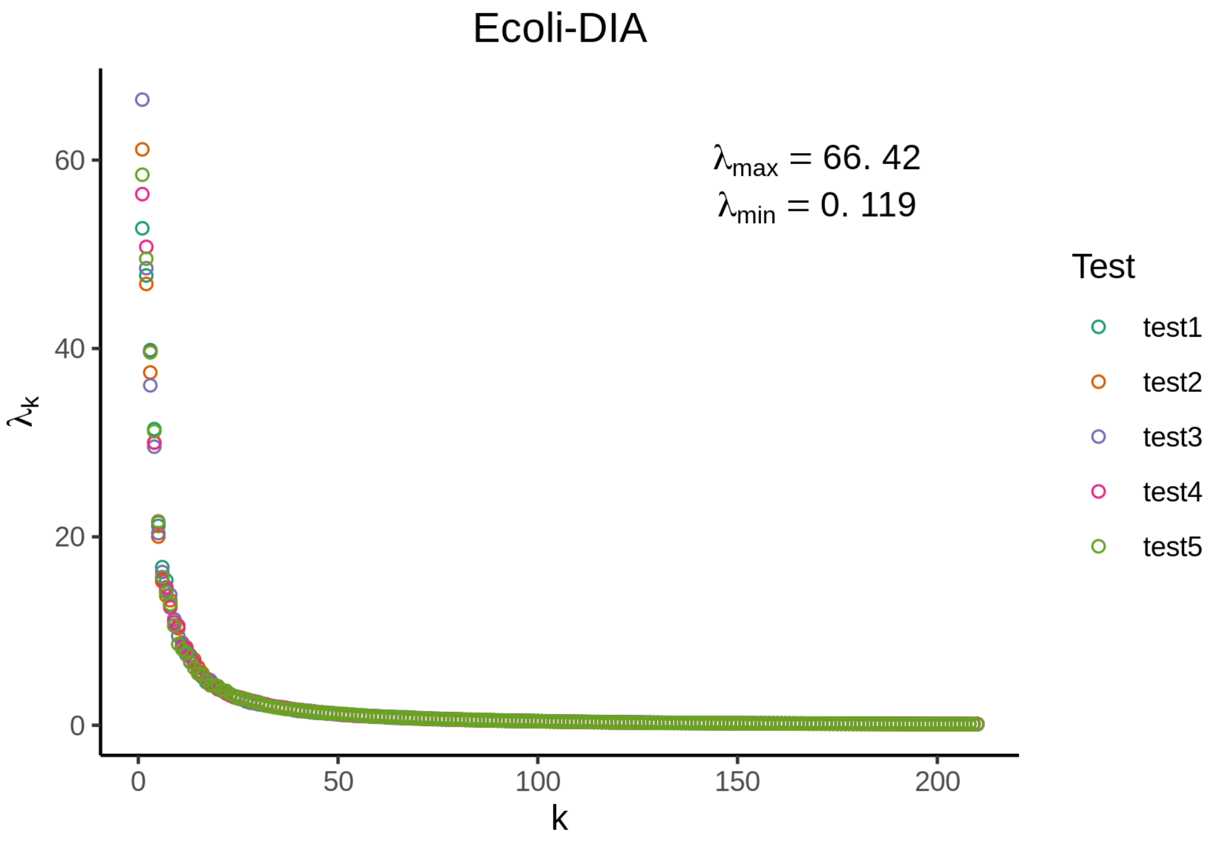

Figure 1: Matrix spectrum for the 5 repetitions (each with a specific color) of Nyström approximation resulting from Ecoli-DIA dataset. The minimal and maximum values (λ_{min} and λ_{max}, respectively) over these 5 tests are indicated in the upper right corner.

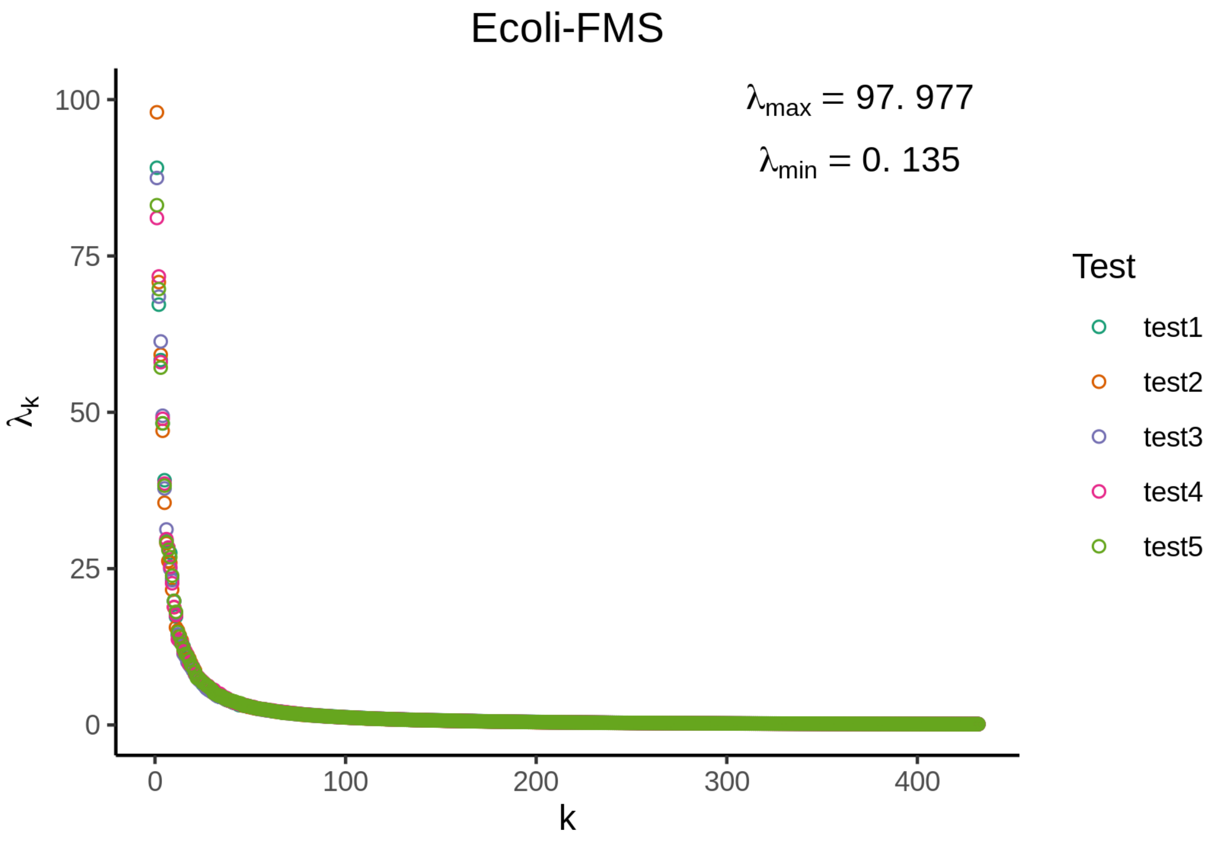

Figure 2: Matrix spectrum for the 5 repetitions (each with a specific color) of Nyström approximation resulting from Ecoli-FMS dataset. The minimal and maximum values (λ_{min} and λ_{max}, respectively) over these 5 tests are indicated in the upper right corner.

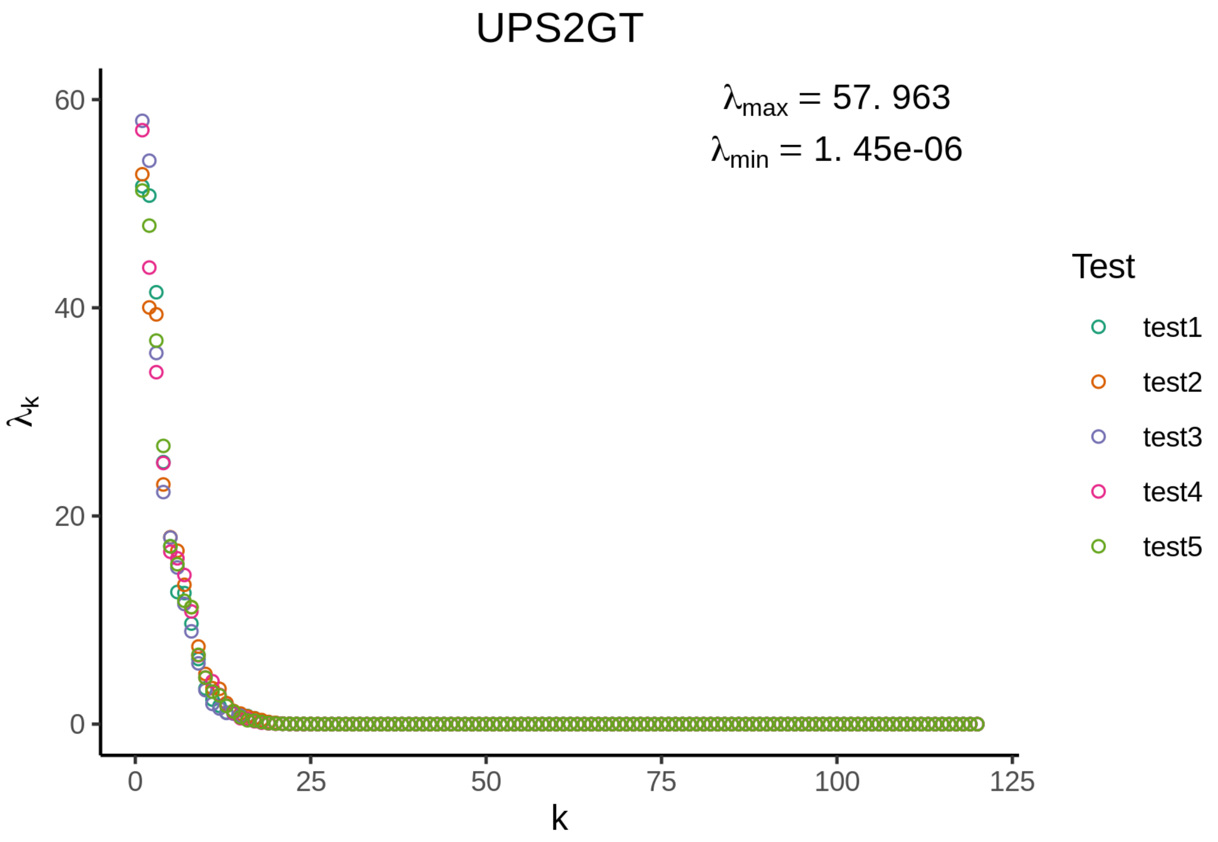

Figure 3: Matrix spectrum for the 5 repetitions (each with a specific color) of Nyström approximation resulting from UPS2GT dataset. The minimal and maximum values (λ_{min} and λ_{max}, respectively) over these 5 tests are indicated in the upper right corner.

Proof. [3] (Corollary 1.13 p. 70). □

Lemma 2. *$\forall x, y \in \mathbb{R}^t$ and $\gamma \in \mathbb{R}_+^*$ the kernel $k_2^\gamma(x,y) = e^{-\gamma \cdot \|x-y\|_{\ell_1}}$ is positive definite.*

Proof. The ℓ_1 norm of a vector x reads

$$\|x\|_{\ell_1} = \sum_{i=1}^{t} |x^i|,$$

where x^i is i^{th} coordinate of x. The kernel $k_2^\gamma(x,y)$ can be rewritten as follows:

$$k_2^\gamma(x,y) = \prod_{i=1}^{t} e^{-\gamma \cdot |x^i - y^i|}$$

where $(e^{-\gamma \cdot |x^i - y^i|})_{i=1}^t$ is a sequence of Exponential 1D kernels, which are positive definite on $\mathbb{R}$ (**Property 2**). Thus, according to **Lemma 1**, $k_2^\gamma(x,y)$ is also positive definite. □

Corollary 1. *The Laplacian W1 kernel (see **Definition 5**) is positive definite.*

Proof. It is sufficient to notice that according the **Definition 2**, the Wasserstein-1 distance $d_{W1}(x,y)$ reads $\|F_x - F_y\|_{\ell_1}$, where F_x and F_y are the empirical cumulative functions, *i.e.* vectors $\in \mathbb{R}^t$. As the set of the empirical cumulative function $X_F = \{F \in \mathbb{R}^t \mid F^1 \leq, \cdots \leq F_t, \sum_{i=1}^t F^i = 1\}$ is a subset of $\mathbb{R}^t$, the positive definiteness of k_{LW1}^γ derives directly from the positive definiteness of k_2^γ (**Lemma 2**). □

References

[1] Roger A Horn and Charles R Johnson. *Matrix analysis.* Cambridge university press, 2012.

[2] Marc G Genton. Classes of kernels for machine learning: a statistics perspective. *Journal of machine learning research*, 2(Dec):299–312, 2001.

[3] Christian Berg, Jens Peter Reus Christensen, and Paul Ressel. *Harmonic analysis on semigroups: theory of positive definite and related functions*, volume 100. Springer, 1984.
